# NKG2D receptor ligands are cell surface biomarkers for injured murine and human nociceptive sensory neurons

**DOI:** 10.1186/s12974-025-03675-1

**Published:** 2025-12-29

**Authors:** Shuaiwei Wang, Allison M. Barry, Yoon Kyung Lee, Naomi Young, Sang Wook Shim, Xinying Wang, Hyeongcheol Kim, Laura Stirling-Barros, Rafael González-Cano, Michael Costigan, Georgios Baskozos, Simon Rinaldi, David LH Bennett, Seog Bae Oh, Alexander J. Davies

**Affiliations:** 1 Nuffield Department of Clinical Neurosciences, University of Oxford, John Radcliffe Hospital, Oxford, OX3 9DU United Kingdom; 2 Center for Advanced Pain Studies, School of Behavioral and Brain Sciences, Department of Neuroscience, University of Texas at Dallas, Richardson, TX 75080 USA; 3 Interdisciplinary Program in Neuroscience, Seoul National University, Seoul, 08826 Republic of Korea; 4 Department of Brain and Cognitive Sciences, Seoul National University, Seoul, 08826 Republic of Korea; 5 Medical Sciences Division, University of Oxford, John Radcliffe Hospital, Oxford, OX3 9DU United Kingdom; 6 Department of Pharmacology, Faculty of Medicine and Biomedical Research Center (Neurosciences Institute), Biosanitary Research Institute, University of Granada, Granada, 18100 Spain; 7 The Department of Anesthesiology, Critical Care and Pain Medicine Boston Children’s Hospital, Harvard Medical School, Boston, MA United States; 8 Department of Neurobiology and Physiology, School of Dentistry, and Dental Research Institute, Seoul National University, Seoul, 03080 Republic of Korea; 9 ADA Forsyth Institute, Somerville, MA USA; 10 InnoCORE PRISM-AI Research Group, Korea Advanced Institute of Science and Technology, Daejeon, Republic of Korea

**Keywords:** Dorsal root ganglia, Human induced pluripotent stem cells, Natural killer cells, Natural killer group 2D, Nerve injury, Neuro-immune, Neuropathic pain, Nociceptors, Non-peptidergic sensory neurons, Pain biomarkers, Retinoic acid early transcript, UL16-like binding protein

## Abstract

Nociceptors are primary afferent neurons that sense noxious stimuli. They can be activated by tissue injury as well as the accompanying local immune response. We have shown that following nerve injury in mice, cytotoxic Natural Killer (NK) cells infiltrate the peripheral nerve where they interact with stress-induced ligands of the activating receptor NKG2D (*Klrk1*). However, the diversity and specificity of NKG2D receptor ligands among sensory neuron subtypes, and translation of this mechanism to human cells, remains unknown. We used dorsal root ganglion (DRG) neurons cultured from C57BL/6J mice of both sexes with fluorescently labelled sensory neuron lines (*Scn10a*, *Mrgprd*,* Calca*,* Trpv1*, *Th*, *Thy1*), as well as human induced pluripotent stem cell-derived (hiPSCd)-sensory neurons after laser ablation, as in vitro models of axonal injury. We assessed the expression of NKG2D ligands by quantitative polymerase chain reaction (PCR) corroborated by publicly available RNA sequencing datasets and validated with single-cell PCR. Recombinant NKG2D receptor proteins were used in live cell-based assays to reveal the subcellular membrane localisation of NKG2D ligands with quantification by semi-automated image analysis. Functional interactions between human NK cells and sensory neurons were confirmed with co-cultures in microfluidic devices. We show that NKG2D ligands are expressed exclusively in unmyelinated DRG neurons after injury. Soluble mouse NKG2D receptors bound to puncta along distal neurites of injured axons enriched predominantly in *Mrgprd*-expressing non-peptidergic nociceptors. We observed low-level binding of soluble human NKG2D receptors to neurites of hiPSCd sensory neurons that increased after axonal laser ablation. Degeneration of hiPSCd sensory neurites by interleukin (IL-2) primed human NK cells was prevented by an NKG2D blocking antibody. The induction and enrichment of functional NKG2D receptor ligands selectively on pathological nerve fibres could aid the diagnosis of peripheral neuropathy in chronic pain conditions, and sheds new light on the potential role of nociceptive neurons in regulating the local tissue immune microenvironment.

**Supplementary Information:**

The online version contains supplementary material available at 10.1186/s12974-025-03675-1.

## Introduction

Nociceptors are a specialized subset of primary afferent somatosensory neurons that respond to noxious stimuli [1]. Housed in the dorsal root ganglia (DRG), these nerve cells are the first part of a neuronal pathway sending signals to brain that are perceived as pain. After injury or disease, peripheral nerves may undergo Wallerian degeneration followed by regeneration [2], as well as alterations to their biochemical and functional properties that are thought to contribute to peripheral sensitization and neuropathic pain [3]. Despite substantial evidence from preclinical studies, many novel neuronally targeted treatments for neuropathic pain have failed in clinical trials [4, 5]. The clinical situation is reinforced by a recent systematic review and meta-analysis of clinical trial data on pharmacotherapeutic interventions, which found a persistent reliance on existing drugs such as tricyclic anti-depressants, serotonin and norepinephrine reuptake inhibitors and α2δ-ligands as first list treatments for neuropathic pain [6]; guidance which has not changed significantly in the last 10 years [7]. Understanding the broader changes in nociceptor form and function after nerve injury is therefore crucial for uncovering the mechanisms of neuropathic pain and developing effective treatments [8].

Recent developments in single-cell sequencing technologies have deepened our understanding of sensory neuron heterogeneity [9], as well as key differences between species [10, 11]. However, our understanding of the pathological changes that occur to DRG neurons is hindered by the loss of transcriptional identity after nerve injury [12]. The resulting changes in expression of sensory neuron-specific drug targets, such as Na_v_1.8 [13], is likely to limit the re-purposing of promising pharmacological treatments for acute pain [14] to chronic neuropathic pain. Knowledge of an injury-specific biomarker, particularly one expressed at the cell surface, would greatly enhance our ability to identify and target pathological sensory nerves for potential therapeutic intervention.

In addition to intrinsic neuronal changes, the immune system has a critical role in peripheral sensitization and pain following nerve injury [15], with bidirectional neuro-immune crosstalk between nociceptors and immune cells influencing the outcomes of pain and inflammation [16, 17]. Our previous work has highlighted the role of cytotoxic natural killer (NK) cells in the immune response to nerve injury and neuropathic pain resolution [18], suggesting immunotherapies targeting innate cytotoxic cells could be a potential strategy for neuropathic pain treatment [19].

NK cells are a key component of the innate immune system and member of the innate lymphoid cell (ILC) family. They perform immuno-surveillance for transformed, virus-infected and stressed cells within the body, as well as regulate adaptive immune responses [20]. Among NK cells’ direct functions are the detection of stress-induced molecules, or ‘altered-self’, via activating receptors [21, 22]. The archetypal activating receptor Natural Killer group 2D (NKG2D), encoded by the gene *Klrk1* in mice (*KLRK1* in humans) [23], serves as an evolutionarily conserved ‘master switch’ regulating NK cell activation in both species [24, 25]. In mice, NKG2D ligands are encoded by the *Raet1* gene family consisting of five gene isoforms: α, β, γ, δ and ε [26, 27]. Other known NKG2D ligands include the multi-gene family histocompatibility 60 (*H60)* a-c [28] and murine UL16-binding protein-like transcript 1 *Mult1 (Ulbp1)* [29], which also contribute to NK cell-mediated cytotoxicity [30]. In humans, NKG2D ligands include MHC class I polypeptide-related sequence A (MICA) and MICB, and the UL16-binding protein family: ULBP1 (RAET1I), ULBP2 (RAET1H), ULBP3 (RAET1N), ULBP4 (RAET1E), ULBP5 (RAET1G), and ULBP6 (RAET1L) [31, 32]. Engagement of the NKG2D receptor with transmembrane or glycophosphatidylinositol (GPI)-linked membrane protein ligands expressed at the cell surface may render the target susceptible to cell-mediated cytotoxicity by NK or CD8+ T cells, and as well as subsets of NKT and γδ T cells [30, 33].

We have previously demonstrated that genes for the *Raet1* family of ligands are upregulated within the DRG following peripheral nerve injury in mice [18]. However, the diversity of NKG2D ligand expression across sensory neuron subtypes, and therefore the cellular specificity of targeting by NK cells after nerve injury, remains undefined. In this study, we aimed to identify the subpopulation of sensory neurons expressing activating ligands for NKG2D after injury, as well as examine the potential for translation of this neuro-immune interaction from mice to humans.

## Results

### DRG neuron culture replicates injury-induced regulation of NKG2D ligands

**Fig. 1:**
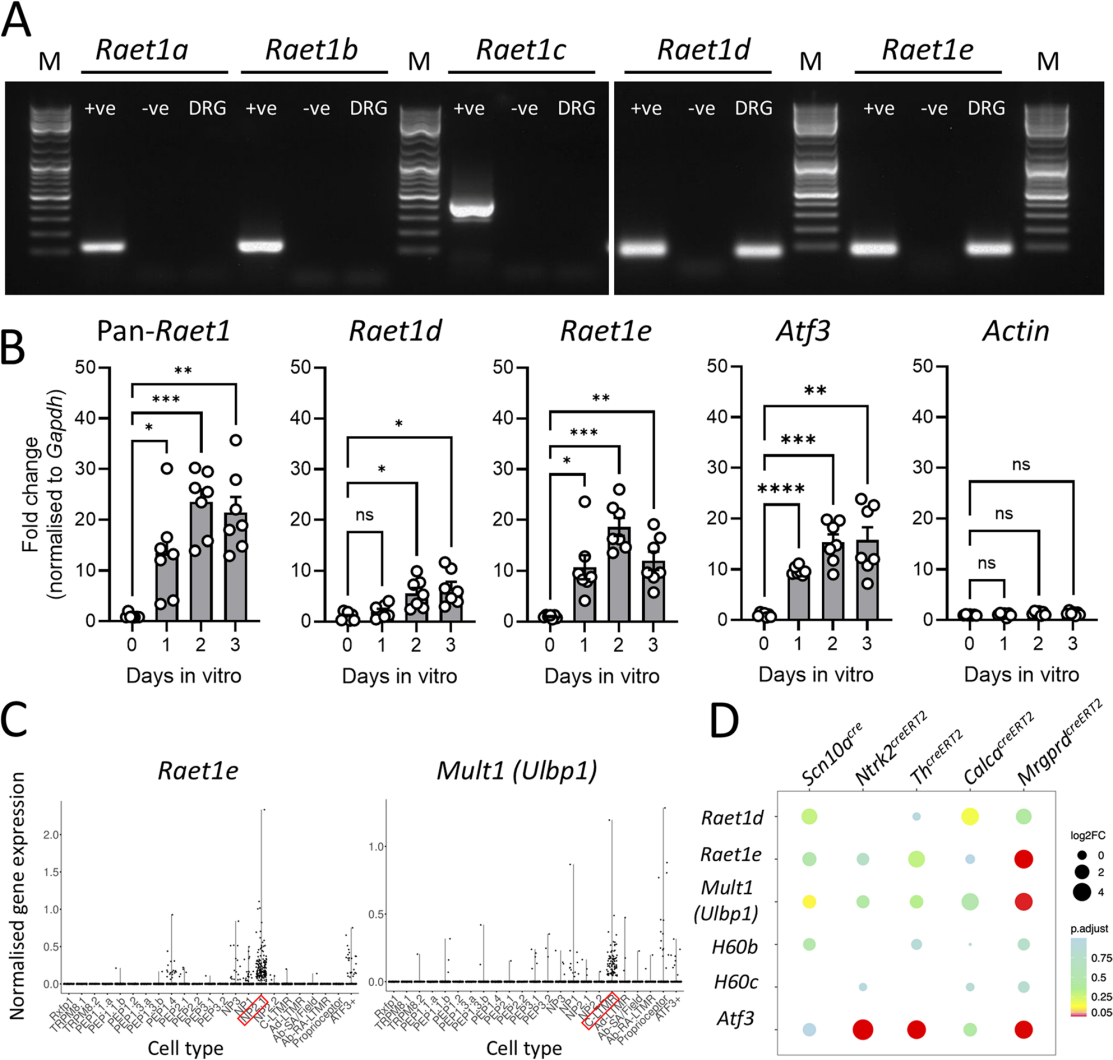
Detection of mRNA transcripts for *Raet1* gene family in murine DRG neurons. **A** PCR gel electrophoresis showing detection of *Raet1d* and *Raet1e* mRNA transcripts only in DRG from C57BL/6J mice. Predicted PCR product sizes for *Raet1a* (111 bp), *Raet1b* (119 bp), *Raet1c* (404 bp), *Raet1d* (67 bp), *Raet1e* (79 bp). +ve, universal RNA positive control; -ve, minus reverse transcription (RT) control reaction; DRG, lysate from DRG neurons after 3 day in vitro (female mouse); M, 100 bp DNA ladder marker. **B** qPCR for pan-*Raet1*,* Raet1d*, *Raet1e*,* Atf3 and Actin* mRNA transcripts in DRG cell cultures over 3 days in vitro. *Gapdh* was used a reference gene. *n* = 7 replicate DRG cultures from different mice (3 m, 4f). Repeat measures ANOVA versus Day 0: *pan-Raet1*, F_(2.547, 15.28)_ = 19.67, *P* < 0.0001; *Raet1d*, F_(1.864, 11.19)_ = 10.51, *P* = 0.0030; *Raet1e*, F_(1.696, 10.17)_ = 24.34, *P* = 0.0002; *Atf3*, F_(1.079, 6.472)_ = 31.19, *P* = 0.0010; *Actin*, F (2.361, 14.17) = 2.881, *P* = 0.0825. Dunnett’s multiple comparison (corrected p values): *p < 0.05, **p < 0.01, ***p < 0.001. **C** Violin plots showing normalised expression of *Raet1e* and *Mult1* (*Ulbp1*) enriched in NP2.1 and C-LTMR mouse DRG neuron subtypes, respectively (highlighted in red boxes). Data from integrated atlas curated by Krauter et al. [41]. **D** NKG2D ligand expression in each of five sensory neuron lineages after nerve injury. Gene expression levels presented as transformed transcript counts in ipsilateral and contralateral lumbar DRG neurons 3 days after spared nerve injury. Data from Barry et al., 2023 [42]

We first sought to identify the *Raet1* gene isoforms expressed by mouse DRG neurons. Transcripts for *Raet1*d and *Raet1*e [27, 34] but not *Raet1a*, *Raet1b* and *Raet1c* were detected in DRG tissue (Fig. 1A), consistent with previous findings in other tissues from C57BL/6J mice [33, 35]. Quantitative PCR of DRG cultures revealed an upregulation of *Raet1*e, and to a lesser extent, *Raet1*d, over several days in vitro, consistent with primers against all (pan-*Raet1*) isoforms (Fig. 1B) [18]. DRG neuron culture also led to an upregulation of the nerve injury-related activating transcription factor *3 (Atf3)* (Fig. 1B) suggesting that DRG neurons in vitro represent an injury-like state [12]. Cell culture had no effect on the ratio of house-keeping genes *Gapdh* and *Actin* (Fig. 1B, *right*). *H60a* is not detected in C57BL/6 mice [36] and, with the exception of the skin, transcripts for H60b and H60c are relatively low [37]. We therefore investigated the expression of the high affinity *Mult1* ligand after peripheral nerve injury. Interestingly, we observed an increase in *Mult1* expression in ipsilateral L5 DRG after spinal nerve transection (Supp. Figure 1A), similar to previous findings with *Raet1* [18]. By contrast, MHC class I-related *Qa1b* and adaptor molecule β2-microglobulin (*B2m*) showed only minor gene regulation in DRG 7 days after L5x compared to sham surgery (Supp. Figure 1B).

To delve deeper into the sensory neuron subsets expressing NKG2D ligand genes we explored published single-cell datasets. Consistent with our PCR results in C57BL/6 mice, transcripts for *Raet1d*, *Raet1e* and *Mult1* (*Ulbp1*), but not *Raet1a*, *Raet1b* and *Raet1c*, were among those detectable in DRG neurons from naïve mice [38–40]. A recently published integrated atlas of over 44,000 deeply sequenced mouse DRG neurons [41] showed *Raet1e* clustering within non-peptidergic DRG neurons, while *Ulbp1* (*Mult1*) was enriched predominantly in tyrosine hydroxylase (*Th*)+ C-low threshold mechanoreceptors (C-LTMRs) (Fig. 1C). In contrast, *Raet1d*,* H60b* and *H60c* were detected at very low levels (Supp. Figure 1C), while *Raet1a*, *Raet1b*, *Raet1c* and *H60a* were undetected (data not shown). To overcome the possible limitations in sequencing depth of single-cell datasets, we analysed a pseudo-bulk RNA sequencing dataset comparing gene expression from pooled single DRG neurons 3 days after spared nerve injury (SNI) [42]. Among the known NKG2D ligands detectable in the dataset of five different labelled sensory neuron lines, *Raet1e* and *Mult1* (*Ulbp1*) transcripts were most abundant and highly expressed in *Mrgprd*-expressing neurons ipsilateral to nerve injury (Fig. 1D and Supp. Figure 1D). Similarly, higher levels of *Raet1e* and *Ulbp1* were expressed by SNI-induced DRG neuronal clusters (SNIICs) in a replicate mouse DRG single-cell dataset (data not shown) [43].

Using cryopreserved DRG tissues from previous nerve injury studies in the lab [18, 42] we performed in situ hybridisation for *Raet1* mRNA using RNAscope. We observed significantly greater mRNA level after nerve crush injury in IB4+ neurons within ipsilateral L4 DRG compared to IB4-negative neurons of wild type mice (Supp. Figures 2A-F) as well as *Raet1* mRNA in *Mrgprd*-positive neurons within ipsilateral DRG (L3-L5) 3 weeks after SNI (Supp. Figure 2G-L), together supporting an enrichment of injury-induced *Raet1* gene expression in non-peptidergic nociceptive sensory neurons.

**Fig. 2:**
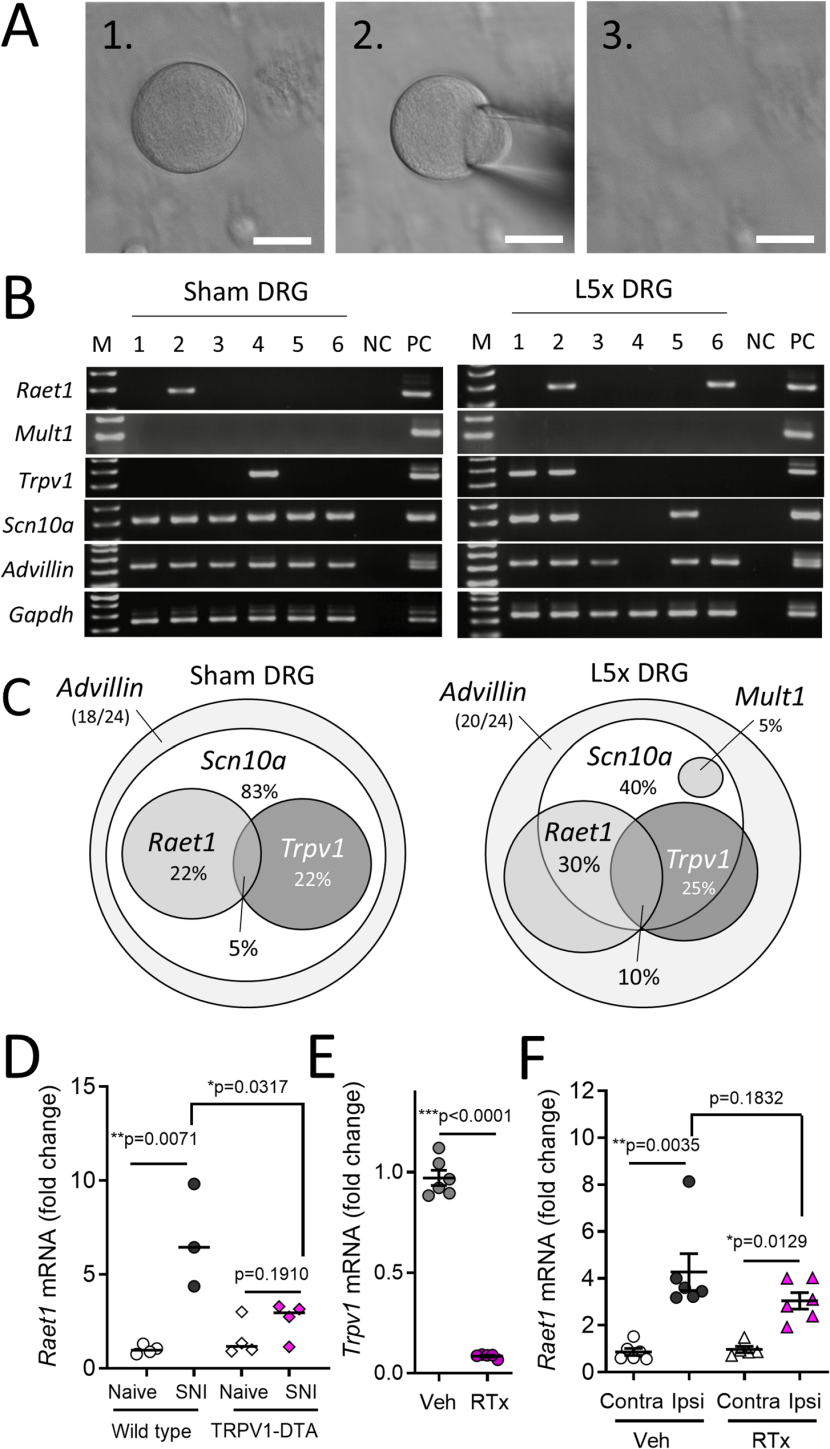
*Raet1* is expressed by individual nociceptive DRG neurons. **A** Consecutive images of single DRG neuron collection by glass micropipette (scale bars, 10 μm). **B** Single-cell nested PCR results for multiple mRNA transcripts detected in L5 DRG neurons isolated from adult male C57BL/6J mice 7 days sham (uninjured) (*left*) or lumbar L5 spinal nerve transection (L5x) surgery (*right*) < 24 h *in vitro.* PCR bands for six DRG neurons are shown with DNA ladder marker, M. NC, negative control (bath solution). PC, positive control (whole DRG tissue). **C** Venn diagrams showing the proportion of *Gapdh+ Advillin+* DRG neurons with transcripts detected for *Scn10a* (Nav1.8), *Raet1* and *Trpv1*, 7 days after sham surgery (*left*) or L5x-injury (*right*). Central overlapping region represents *Raet1* + *Trpv1 +* double-positive DRG neurons. **D** Increase in *Raet1* mRNA expression in ipsilateral L4-5 DRG 7 days after spared nerve injury (SNI) is attenuated in *Trpv1*^*cre/wt*^;*rosa26*^*dta/wt*^ mice. Student’s unpaired t test: SNI *versus* naïve wild type, t = 4.395, *p* = 0.0071; SNI *versus* naïve *Trpv1-dta*, t = 1.474, *p* = 0.1910. *n* = 3–4 mice per surgery per genotype. **E** qPCR showing the effect of neonatal resiniferatoxin (RTx) treatment on *Trpv1* mRNA expression in lumbar L3-5 DRG compared to vehicle. Student’s unpaired t test: t = 20.99, *p* < 0.0001. n=6 mice per group. **F** qPCR showing increase in *Raet1* mRNA expression in ipsilateral L3-5 DRG 7 days after SNI surgery both in vehicle and RTx-treated adult male C57BL/6J mice. Student’s paired t test: Vehicle, contra *versus* ipsi, t = 5.200, **p = 0.0035; RTx, contra *versus* ipsi, t = 4.278, *p = 0.0129; Ipsi, Veh *versus* RTx, t = 1.430, *p* = 0.1832. *n* = 6 mice per treatment

### NKG2D ligand genes are detectable at single DRG neuron resolution

To validate the expression of NKG2D ligands after injury at the single-cell level we analysed pan-*Raet1* and *Mult1* transcripts within individual DRG neurons collected from acute cultures of DRG neurons (< 24 h in vitro) from adult C57BL/6 mice 7 days after sham or L5-spinal nerve transection (L5x) surgery (See **Methods**). A total of 18 sham and 20 L5x cells out of 24 single cells collected per group were positive for *Gapdh* and *Advillin* transcripts indicating successful DRG soma collection (Figs. 2A, B and Supp. Figures 3A, B). Due to a limited amount of PCR product, we restricted our analysis to a small group of genes. Pan-*Raet1* transcripts were observed in 20–30% of identified neurons (Fig. 2B and Supp. Figures 3A, B), with no difference in frequency between cultures from sham and L5x injured mice (*p* > 0.999, Fisher’s exact test). Only a small number of neurons (5–10%) co-expressed transcripts for both *Raet1* and *Trpv1*. In sham DRG cultures all *Raet1* and/or *Trpv1*-expressing neurons were positive for *Scn10*a (Fig. 2C, *left*) encoding the voltage-gated Na^+^ channel Na_v_1.8. Fewer neurons from L5x DRG were positive for *Scn10*a (Fig. 2C, *right*) consistent with the downregulation of Na_v_1.8 after nerve injury [13]. Detection of *Mult1* mRNA transcripts in DRG neurons at the single cell level - either sham or L5x injured - was rare in our sample population (Fig. 2C and Supp. Figures 3A, B). To confirm the identity of small-sized DRG neurons in the nerve injury group we additionally tested primers for the transcription factor *Runx1* and nerve growth factor (NGF) receptor *Trka* [44]. All *Raet1* and *Trpv1* neurons were positive for *Runx1*, while only around 50% were positive for Trka (Supp. Figures 3C, D). The proportion of *Runx1* and *Trka* transcripts among *Raet1*-expressing neurons was consistent between sham (Supp. Figure 3C) and L5x DRG (Supp. Figure 3D). The transcriptional profile of *Raet1* neurons (expressing *Runx1* and partial overlap with *Trka* and *Trpv1*) was in keeping with the characterisation of *Trpv1*-*lineage* neurons [45]. To confirm this we crossed a *Trpv1*-driven Cre-recombinase expressing mouse (*Trpv1*^*cre*^) [46] with a diphtheria toxin subunit A (DTA) reporter mouse to ablate the *Trpv1*-*lineage* neurons, which significantly attenuated the upregulation of *Raet1* in DRG 7 days after peripheral nerve injury (Fig. 2D). On the other hand, chemical ablation of Trpv1-expressing cells by neonatal treatment with the potent agonist resiniferatoxin (RTx) (Fig. 2E and Supp. Figure 3E) did not prevent the nerve injury-induced expression of *Raet1* in ipsilateral DRG tissue (Fig. 2F), consistent with enrichment of *Raet1* in a non-peptidergic nociceptor population that downregulates *Trpv1* during development [46].

**Fig. 3:**
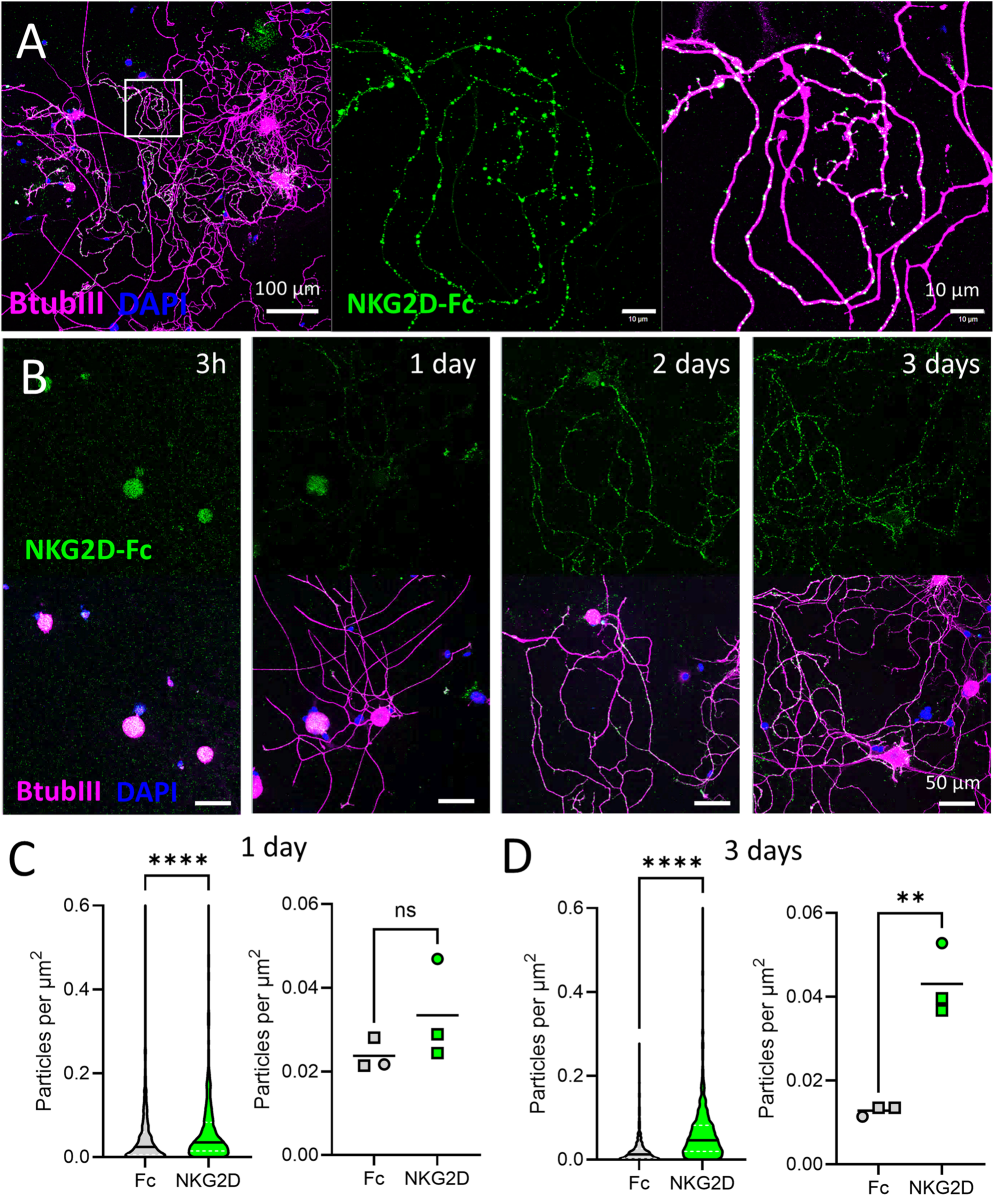
Soluble NKG2D receptor binding to DRG neurite membrane increases over time in culture. **A** *Left*) Low magnification confocal image of DRG neurons 3 days in vitro. *Right*) High magnification of inset. NKG2D-Fc chimeric protein labelling (*green*) of BtubIII+ neurites (*magenta*). DAPI, *blue*. **B** Representative images of NKG2D-Fc binding to DRG neurons over time in culture. Time points as indicated. Scale bars, 50 μm. **C** Quantification of NKG2D-Fc binding versus Fc-only control on day 1 in vitro. *Left*) Violin plot of particle density per µm^2^ of neurite area per image: Fc, *n* = 609 images; NKG2D-Fc, *n* = 661 images. ****p = < 0.0001, Kolmogorov-Smirnov test (D = 0.1389). *Right*) Median particle density per mouse; ns, *p* = 0.2512, t = 1.340, two-tailed unpaired t-test. **D** Quantification of NKG2D-Fc binding versus Fc-only control on day 3 *in vitro. Left*) Violin plot of particle density per µm^2^ of neurite area per image: Fc, *n* = 707 images; NKG2D-Fc, *n* = 769 images. ****p = < 0.0001, Kolmogorov-Smirnov test (D = 0.4296). *Right*) Median particle density per mouse; **p = 0.0038 (t = 6.042) two-tailed unpaired t-test. *n* = 1f (*circle*), *n* = 2 m (*squares*) mice per time point

In summary, *Raet1e* and *Mult1* (*Ulbp1)* were the two main NKG2D ligand transcripts enriched in unmyelinated sensory neurons and upregulated by peripheral nerve injury. *Raet1e* is predominantly restricted to a subpopulation of nociceptive DRG neurons characterised by the developmental expression of *Scn10a* and *Trpv1*; *Mult1* (*Ulbp1)* appears to have a more limited RNA expression, possibly in *Th* + C-LTMRs.

### NKG2D ligands are detectable in live cell-based assays

To demonstrate functional relevance of these findings, we next sought to validate the detection of NKG2D ligands at the protein level using a live cell-based assay approach [27]. We first validated a source of soluble recombinant NKG2D receptor protein chimerised to the Fc domain of human IgG1 (NKG2D-Fc) to recognise the high affinity ligand mouse Raet1e by heterologous expression in HEK293T cells (Supp. Figure 4A). Incubation of live cells with 2 µg/ml of NKG2D-Fc chimeric protein, but not an equivalent amount of Fc-only control protein, bound to the surface of cells transfected with *Raet1e* but not untransfected controls (Supp. Figure 4B).

Application of NKG2D-Fc to live DRG neurons (3 days in vitro) under the same conditions, revealed punctate labelling of some but not all BtubIII+ neurites (Fig. 3A). NKG2D receptor binding to DRG neurites increased over time (Fig. 3B), paralleling the upregulation of *Raet1e* transcript (Fig. 1B). Quantification of immunofluorescence signal density on neurites confirmed significantly greater detection of NKG2D-Fc receptor binding versus Fc control by day 3 in vitro (Fig. 3C, D). Mouse NKG2D-Fc chimeric protein, but not human NKG2D-Fc or Fc-only control, bound to cultured mouse DRG neurites, confirming the species specificity of the recombinant receptor proteins (Supp. Figures 4C-E).

### NKG2D receptors bind to specific sensory neuron subsets

**Fig. 4:**
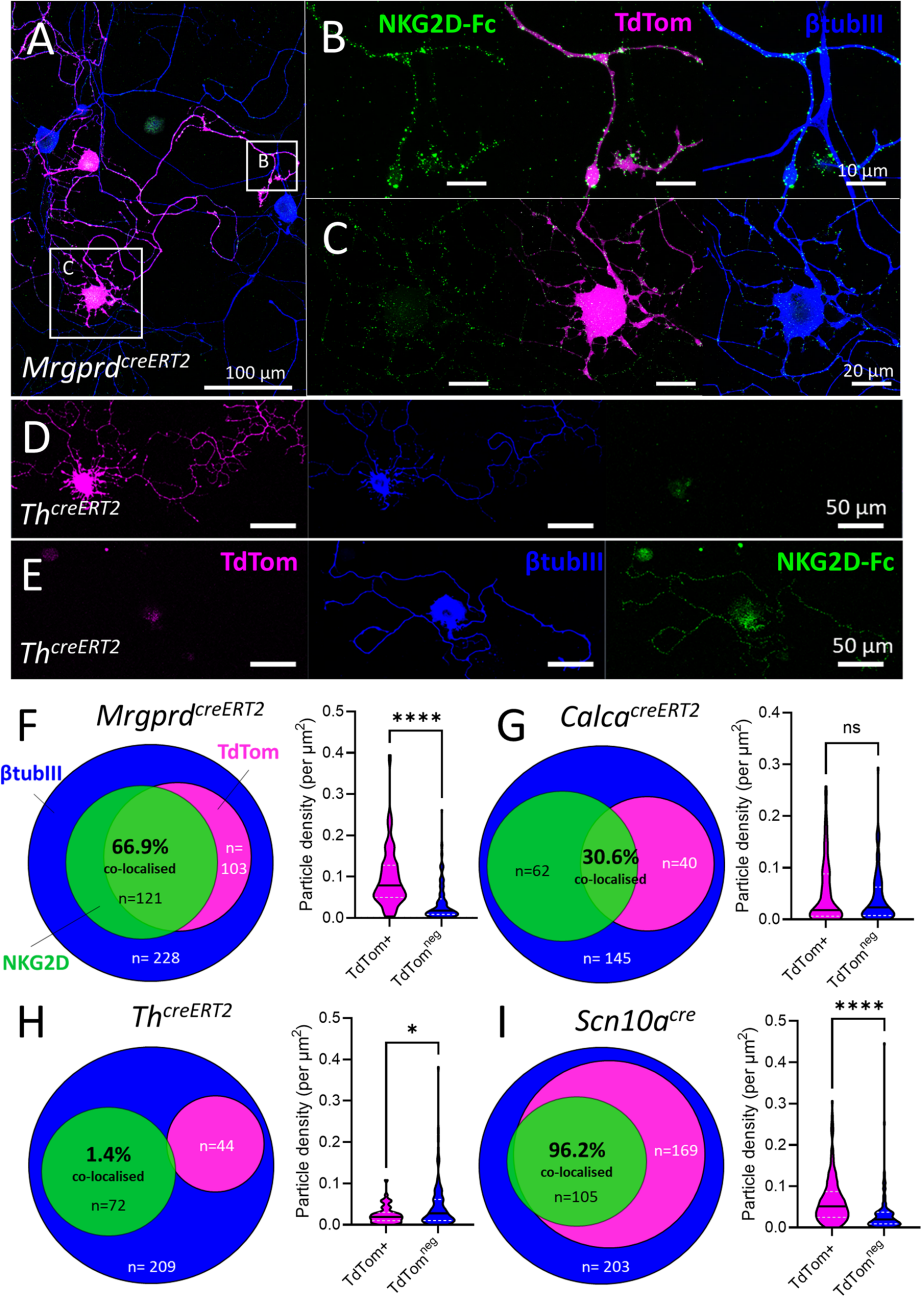
NKG2D receptor binding is enriched among non-peptidergic nociceptive neurons. DRG neurons were cultured for 3 days prior to live cell labelling with NKG2D-Fc. **A** Low magnification confocal image of DRG neurons from *Mrgprd-cre*^*ERT2*^;*TdTomato* mice 3 days in vitro. **B**,** C** High magnification images of insets in (A). Note density of NKG2D-Fc receptor binding to neurite terminals (B) compared to cell body (C) of TdTom+ neuron. **D**,** E** Low magnification confocal images of DRG neurons from *Th-cre* ^*ERT2*^;*TdTomato* mice 3 days in vitro. Note lack of NKG2D-Fc receptor binding to neurites of TdTom+ neuron (D) compared to TdTom^neg^ neuron (E). Scale bars as indicated. **F-I** Quantification of NKG2D receptor binding to the neurites of DRG neurons from four different genetic sensory neuron subsets. Venn diagrams illustrate the proportions of DRG neurons displaying NKG2D binding assessed by manual counting. Violin plots illustrate NKG2D receptor particle density per µm^2^ of neurite area per image. **F** *Mrgprd-cre*^*ERT2*^;*TdTomato* (non-peptidergic) sensory neurons. Manual counting: *n* = 228 neurons from *n* = 3 mice; 2 male, 1 female. Automated image analysis: TdTom^+^ neurites, *n* = 109 images; TdTom^neg^ neurites, *n* = 129 images; ****p = < 0.0001, Kolmogorov-Smirnov test (D = 0.5713). **G** *Calca*-*cre*^*ERT2*^;*TdTomato* (peptidergic) sensory neurons. Manual counting: *n* = 145 neurons from *n* = 5 mice; 1 male, 4 female. Automated image analysis: TdTom^+^ neurites, *n* = 67 images; TdTom^neg^ neurites, *n* = 95 images; ns, *p* = 0.5463, Kolmogorov-Smirnov test (D = 0.1274). **H** *Th*-*cre*^*ERT2*^;*TdTomato* (C-LTMR) sensory neurons. Manual counting: *n* = 209 neurons from *n* = 4 mice; 3 male, 1 female. Automated image analysis: TdTom^+^ neurites, *n* = 51 images; TdTom^neg^ neurites, *n* = 100 images; *p = 0.0472, Kolmogorov-Smirnov test (D = 0.2355). **I** *Scn10a-cre; TdTomato* (Nav1.8 nociceptive) sensory neurons. Manual counting: *n* = 203 neurons from *n* = 4 mice; 2 male, 2 female. Automated image analysis: TdTom^+^ neurites, *n* = 162 images; TdTom^neg^ neurites, *n* = 128 images; ****p = < 0.0001, Kolmogorov-Smirnov test (D = 0.4310). βtubIII counterstain, *blue*. Median and quartiles represented within violin plots as black and white dotted lines, respectively

We next asked whether NKG2D-Fc receptor binding preferentially targeted one or more sensory neuron subtypes, as suggested by our earlier transcriptomic analysis. Immunohistochemical markers for sensory neurons, such as calcitonin gene related peptide (CGRP) or isolectin B4 (IB4) for non-peptidergic nociceptors in rodents, are liable to change after nerve injury in culture [47, 48]. We therefore used a range of transgenic reporter mice in which TdTomato (TdTom) expression is driven by canonical marker genes in a cre-dependent, tamoxifen-inducible manner [42]. DRG neurons from *Mrgprd*^*creERT2*^ mice (a marker for putative mechanosensitive non-peptidergic sensory neurons) [49], showed a high degree of binding by NKG2D-Fc in TdTom+ neurons (Fig. 4A), with receptor density highest near the terminals of labelled neurites (Fig. 4B), compared to cell bodies (Fig. 4C). On the other hand, DRG neurons from *Th*^*creERT2*^ mice (labelling tyrosine hydroxylase expressing C-low threshold mechanoreceptors) [40, 50] showed little to no immunoreactivity in TdTom+ neurons (Fig. 4D) compared to TdTom-negative neurons (Fig. 4E).

Using both blinded manual and automated quantification of NKG2D-Fc binding of TdTom-positive and negative neurons from each genetic lineage, we observed the greatest enrichment in *Mrgprd*+ neurons (Fig. 4F), followed by *Calca*+ neurons (encoding peptidergic marker CGRP) (Fig. 4G), which showed a partial overlap between NKG2D-Fc binding and TdTom+ labelling (Supp. Figures 5A-H). Super-resolution confocal imaging revealed a high density of receptor-bound puncta along the surface of TdTom+ DRG neurites from *Mrgprd*^*creERT2*^ mice after 3 days in vitro (Supp. Video 1). No significant NKG2D-Fc labelling was observed in *Th*+ DRG (Fig. 4H). Almost all NKG2D-Fc labelled neurons could be identified by expression of *Scn10a* (Fig. 4I). Similarly, NKG2D-Fc binding was enriched on nociceptive *Trpv1*-lineage neurons (Supp. Figures 6A, C), but not *Thy1*-lineage neurons, which label predominantly medium and large diameter DRG neurons [51] (Supp. Figures 6B, D).

**Fig. 5:**
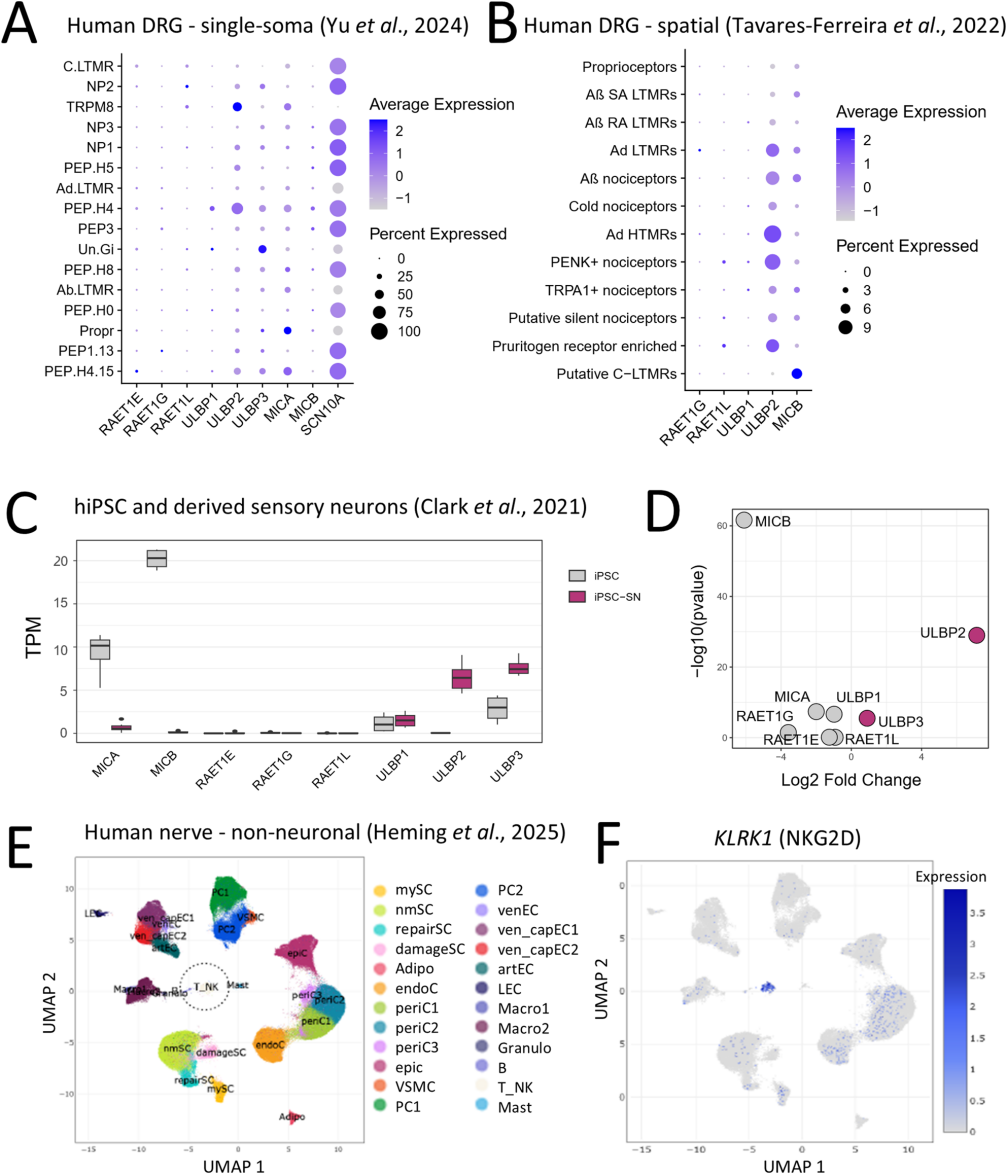
Expression of NKG2D ligands and receptor in human nerve tissues and iPSC models. **A** Single-soma RNA sequencing of six lower thoracic and lumbar DRG of three human donors. Data from Yu et al. [52]. **B** Spatial (‘Visium’) RNA sequencing of human lumbar DRG tissues collected from four male and four female organ donors. Data from Tavares-Ferreira et al. [53].* RAET1E*, *ULBP3* and *MICA* were not detected. **C** Bulk RNA sequencing of hiPSC and hiPSCd sensory neurons. Log-fold enrichment of NKG2D receptor ligand genes during differentiation of sensory neurons from hiPSC (including lines SFC-AD2-01 (synonym AD2-1, termed ‘AD2’ throughout the study) and SFC-840-03−03 (synonym AH017, termed ‘840’ throughout the study). **D** *ULBP2* and *ULBP3 *transcript levels show the greatest enrichment (transcripts per million, TPM) after sensory neuron differentiation. Data from Clark et al. [54]. **E**, **F** Identification of 23 cell populations including NK/T cells in human sural nerve by analysing published human sural nerve single nuclear RNA-sequencing data. Data from Heming et al. [55]. **F**) Enrichment of *KLRK1* in NK/T cell subset.

### NKG2D ligands are regulated in human sensory neurons

We next investigated whether our findings from mice may have any relevance to human sensory neurons by analysing the expression of known NKG2D ligand genes *MICA*, *MICB*, *ULBP1*, *ULBP2*, *ULBP3*, *ULBP4* (*RAET1E*), *ULBP5* (*RAET1G*), and *ULBP6* (*RAET1L*) [32] in published human tissue RNA sequencing datasets.

Findings from two independent bulk RNA sequencing datasets indicated *MICA* (encoding MHC class I polypeptide–related sequence A) as the highest enriched NKG2D ligand gene in primary human DRG tissue and after cell culture [56] (Supp. Figure 7A). There was no difference between *MICA* levels in donors with and without pain [57] (Supp. Figure 7B). A different pattern was observed in higher resolution single DRG soma [52] (Fig. 5A) and spatial (Visium) datasets [53] (Fig. 5B) with *ULBP2*, in particular, featuring predominantly among nociceptive neuron subsets. *MICA*, *MICB*, *ULBP1*, *ULBP3*, *RAET1E*, *REAT1G* and *RAET1L* were inconsistently detected across datasets, being expressed at low levels or were not detectable (Fig. 5A, B). Overall, these findings indicate that NKG2D ligands are expressed in human DRG, likely by nociceptive neurons.

We next compared expression of the same NKG2D ligand genes in human induced pluripotent stem cells (hiPSC) as an approximation of human sensory neurons in a dataset that included lines ‘840’ and ‘AD2’ [54]. Differential gene expression analysis between hiPSC and hiPSC-derived (hiPSCd) sensory neurons identified *ULBP2* as the most upregulated NKG2D ligand gene, while *MICA* and *MICB* were the genes most downregulated after differentiation to sensory neurons (Fig. 5C, D).

Finally, we reanalysed recently published datasets of human neural tissue to understand the potential interaction with NKG2D expressed by localised immune cells. By extracting and re-clustering immune cells present in human DRG tissue [11] (Supp. Figure 7C), we identified 13 immune subsets, including one (cluster 9) that expressed several of the 13-gene signature that is characteristic of human NK cells [58] (Supp. Figure 7D) and was enriched for *KLRK1*, the gene encoding NKG2D (Supp. Figures 7E, F). *KLRK1* was also expressed by an NK/T cell cluster detected in human sural nerve tissue isolated from patients with polyneuropathy [55] (Fig. 5E, F), placing the NKG2D receptor in the same tissue context as NKG2D ligands potentially expressed by pathological sensory neurons.

### Human NK cells functionally interact with human sensory neurons

**Fig. 6:**
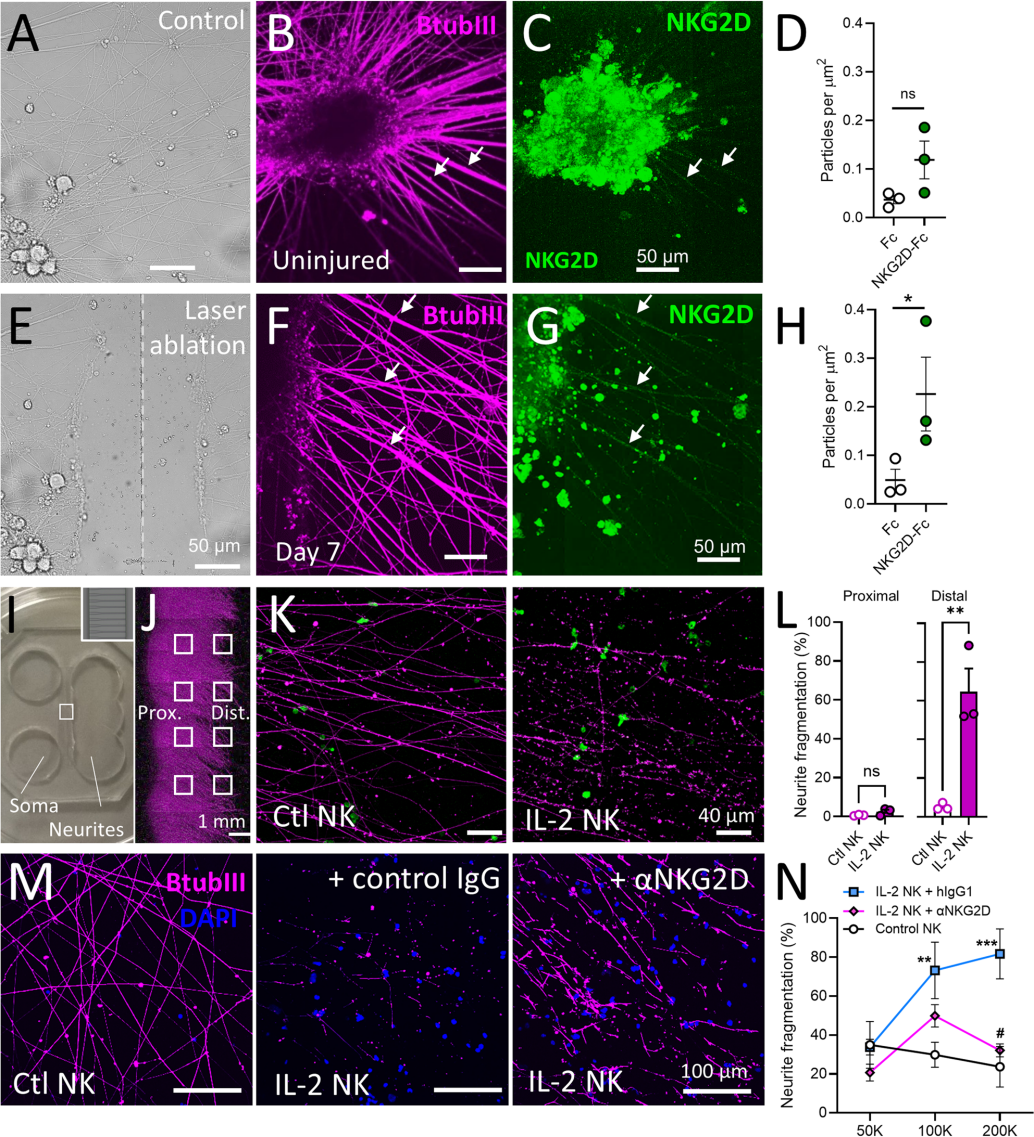
Human iPSC-derived sensory neurons upregulate NKG2D ligands in response to axonal injury and are targets for IL-2 primed human natural killer cells.** A** Bright-field image of uninjured iPSC-derived sensory neurons. **B** βtubIII (*magenta*) and **C** NKG2D-Fc (*green*) immunolabelling of iPSC sensory neurons (‘840’ line) without injury. **D** Quantification of NKG2D-Fc receptor particle density on βtubIII + axons compared to Fc-only controls (ns, *p* = 0.4024). **E** Bright-field image of iPSC-derived sensory neurons immediately after axonal injury by laser ablation. **F** βtubIII (*magenta*) and **G** NKG2D-Fc (*green*) immunolabelling of iPSC sensory neurons (‘840’ line) 7 days after laser ablation. Arrows highlight axons labelled with NKG2D. **H** Quantification of NKG2D-Fc receptor particle density on βtubIII + axons compared to Fc-only controls. *n* = 3 experimental repeats, 2 wells per group, per repeat. Two-way ANOVA, NKG2D versus Fc: F_(1, 8)_ = 8.542, *p* = 0.0192. Sidak’s multiple comparison test: control ns, *p* = 0.4024; ablation, *p = 0.0442. **I** Photo of microfluidic hiPSCd sensory neuron cultures. Sensory neuron precursors were seeded in left hand ‘Soma’ chamber. Axons grow through microfluidic channels into neurite chamber on right hand side. *Inset*) High magnification of microfluidic channels. **J** βtubIII immunolabelling of iPSC sensory axons in neurite compartment. Eight regions of interest (*white squares*) were sampled for proximal and distal axon/neurite quantification. **K** Example ROI of distal axons/neurites from microfluidic devices treated with freshy thawed (control, *left*) or IL-2 primed human NK cells (*right*). NK cells in *gre*en. **L** Quantification of axon/neurite fragmentation. *n* = 3 experimental repeats (n=6 microfluidic devices; *n* = 8 ROI per device) with NK cells from three different healthy donors; sensory neurons derived from iPSC donor ‘AD2’. *Proximal* axon fragmentation ns, *p* = 0.1610 (t = 1.717); *Distal* axon fragmentation **p = 0.0079 (t = 4.931), unpaired t test. **M **Example ROIs of distal axons/neurites from microfluidic devices treated with freshy thawed (unstimulated, *left*), IL-2 stimulated NK cells incubated with IgG1 (*middle*), or IL-2 stimulated NK cells pre-incubated with anti-NKG2D (*right*). NK cells in blue (DAPI), axons in magenta (BtubIII). Scale bars as indicated. **N** Effect of NKG2D blocking antibody on NK cell-mediated axon/neurite fragmentation. Microfluidic devices containing sensory neurons derived from iPSC donor ‘840’ were exposed to different numbers of NK cells (50 × 10^3^, 100 × 10^3^, 200 × 10^3^) from one of three different healthy donors. *n* = 3 experimental repeats (3 microfluidic devices; *n* = 10 ROI per device). Two-way ANOVA: Effect of treatment (F_(2,18)_ = 11.72, *p* = 0.0006); Effect of NK cell number (F_(2, 18)_ = 4.377, *p* = 0.0282). Šídák multiple comparisons: control NK v IL-2 NK (+ IgG) (**p = 0.0095, ***p = 0.0008, adjusted p values); IL-2 NK (+ IgG) v IL-2 NK (+ anti-NKG2D) (^#^*p* = 0.0033, adjusted p value)

To investigate the functional relevance of NKG2D ligand gene expression we differentiated hiPSC into ‘nociceptor-like’ sensory neurons using a well-characterised protocol [59–62]. When matured in vitro for over 40 days, hiPSCd sensory neurons displayed characteristic clusters of cell bodies (neuronal soma), with extending BtubIII+ axons/neurites (Fig. 6A, B). To investigate if human sensory neurons also regulate NKG2D ligands after injury, and to replicate the injury state of primary cultured mouse DRG, we induced axonal injury in hiPSCd sensory neurons in vitro using laser ablation (Fig. 6E). One week after axotomy, axons re-grew across the midline of the ablation site (Fig. 6F and Supp. Figure 8A). In cultures treated with recombinant human NKG2D-Fc receptors or Fc control proteins (2 µg/ml) we observed a non-significant trend for NKG2D-Fc to bind to uninjured hiPSCd sensory neuron axons compared to Fc only (Fig. 6C, D and Supp. Figure 8B). By 7 days after laser axonal ablation human NKG2D receptor binding to axons was significantly higher compared to Fc control (Fig. 6G, H and Supp. Figure 8C).

We next asked whether NKG2D receptor ligand expression on human sensory axons had any consequences for human NK cell interactions. To address this possibility, we adapted our previous murine NK-sensory neuron co-culture platform [63] to develop a humanised co-culture system combining human iPSC-derived sensory neurons (line ‘AD2’) grown in microfluidic devices (Fig. 6I and J) with human NK cells enriched from the blood of healthy donors by magnetic associated cell sorting (MACS) (Supp. Figures 9A, B). Flow cytometry of NK cells showed higher levels of NKG2D (Supp. Figures 9C-E), as well and perforin and granzyme B (Supp. Figures 9F-I), after priming with human interleukin-2 (IL-2) (1000 U/ml for 2 days) compared to untreated controls, confirming a cytotoxic gain-of-function. Addition of NK cells to the neurite compartment of microfluidic devices containing hiPSCd-sensory neurons resulted in fragmentation of distal axons by IL-2 primed NK cells (Fig. 6K, L), reminiscent of the effect seen in murine NK-DRG co-cultures [18, 63].

Our findings of possible low-level binding of NKG2D receptors in non-axotomised hiPSCd sensory neurons prompted us to investigated whether human NKG2D receptor-ligand interactions may play a role in this neurodegenerative interaction, this time using a different hiPSC cell line (‘840’) **(**Fig. 6M). Pre-treatment of the IL-2-primed human NK cells with an NKG2D blocking antibody led to a reduction in axon fragmentation compared to controls (Fig. 6N), suggesting that NKG2D receptor-ligand interactions may contribute to the degeneration of human sensory axons by primed NK cells.

## Discussion

In this study, we used a combination of transcriptomic and targeted protein analysis to identify surface expression of NKG2D ligands by injured nociceptive neurons in mice and humans. These findings have three important implications: First, they provide a molecular basis for understanding the potential neuropathic pain-resolving capacity of NK cells by targeting a subset of non-peptidergic nociceptive nerve fibres known to be crucial for the neuropathic phenotype in mice after traumatic nerve injury. Secondly, the specific ligands identified in both mice and humans in this study could serve not only as unique markers of pathological nerve injury but also targets for novel diagnostic methods and ultimately therapeutic intervention. Third, these findings reinforce the potential for pathological nociceptors to modulate the local immune microenvironment through the expression of ligands for potent immune receptors.

### Molecular identity of murine DRG neurons expressing NKG2D ligands

In healthy tissues, NKG2D ligands are expressed at low or non-detectable levels. We therefore adopted several complementary approaches to triangulate the genetic and functional identity of mouse sensory neurons expressing NKG2D ligands after nerve injury.

We show that *Raet1e* is most likely the predominant isoform expressed by injured nociceptive DRG neurons in C57BL/6 mice (Fig. 1). *Raet1e* displays 25-fold higher affinity for the NKG2D receptor than *Raet1d* [64], suggesting that the former is likely to be the more functionally relevant neuro-immune interaction in the context of peripheral nerve injury [29]. Although a number of NKG2D ligand genes appear in publicly available RNA sequencing datasets of mouse DRG, *Raet1e* was not reliably identified as differentially expressed after nerve injury [65, 66], or in any specific subset(s) of ‘healthy’ DRG neurons [38, 40, 67]. To overcome the potential limitations in sequencing depth in single cell datasets, we analysed transcripts from pooled DRG neurons enriched for neuronal subsets [42]. This approach not only confirmed regulation of individual NKG2D ligand genes *Raet1e* and *Mult1* (*Ulbp1*) by nerve injury but also provided clues to the subset involved (*Mrgprd*-expressing, non-peptidergic neurons) (Fig. 1).

Using highly sensitive, single cell nested PCR, we were able to confirm *Raet1* gene expression in individual DRG neurons. The ubiquitous expression of *Runx1* and high prevalence of *Scn10a* among single cells collected for PCR again reinforced their identity as either nociceptors (Fig. 2) [68] or C-LTMRs [41], while only around 50% were positive for Trka, a transcriptomic marker for peptidergic neurons [44]. Interestingly, there was no difference in the proportion of DRG neurons expressing *Raet1* after L5x injury or sham surgery, suggesting that the increase in transcript we observed by cell culture in vitro, or after injury in vivo, occurred within the same population of neurons. We also did not observe any noticeable sex-differences in our data suggesting that NKG2D ligand regulation is a likely feature of nerve injury in female as well as male mice.

### A live cell-based assay approach for the identification of DRG neurons targets

To overcome the potential discrepancy between transcript and protein levels, and to better understand the dynamics of cell surface expression, we adopted a live cell-based assay approach to identify NKG2D ligands on sensory neurons [27]. Live cell assays have proved to be exquisitely sensitive to identify surface binding to neuronal antigens in heterologous systems [69], as well as live neurons [70]. We revealed soluble NKG2D receptor binding to discrete puncta along the neurites of DRG (Fig. 3) that mirrored the timeline of transcriptional upregulation in culture (Fig. 1) and may reflect the clustering of NKG2D ligands in specific lipid microdomains [71]. The apparent enrichment of NKG2D receptor binding to distal neurites is reminiscent of Raet1 protein labelling in chronically injured sciatic nerve in vivo [18] and provides an anatomical framework for potential cytotoxic neuro-immune interactions at the distal nerve injury site [72]. Whether the peripheral compartmentalisation of receptor ligands in DRG neurons occurs due to axonal transport, or local RNA translation, remains to be investigated.

The process of culturing DRG neurons – effectively an axotomy – has long been known to affect their biophysical properties [48]. The parallel upregulation of *Raet1e* and *Atf3* emphasise that DRG neurons enter an injury-like state in vitro (Fig. 1B). The use of genetically-encoded fluorescent reporter mouse lines allowed us to overcome the potential loss of biochemical markers induced by the cell culture process. Consistent with our transcriptional analysis of NKG2D ligands, soluble NKG2D receptors bound exclusively to neurons of a nociceptive lineage identified by developmental expression of *Scn10a* (Fig. 4) and *Trpv1*. Conversely, the predominant lack of recognition of *Thy1*-lineage fibres suggests that large diameter fibres are not a major target for NKG2D recognition [51]. Among nociceptors, we observed preferential receptor binding to neurites of the *Mrgprd*-expressing (non-peptidergic) subpopulation of DRG neurons, with recognition of a smaller proportion of *Calca*-expressing (peptidergic) neurons (Fig. 4). This finding is consistent with the single-cell PCR result where around 50% of neurons expressing *Raet1* were also positive for peptidergic marker TrkA (Supp. Figure 3) and with data from RNA sequencing of DRG neurons from the same mouse lines after nerve injury (Fig. 1) [42].

Interestingly, *Th*+ low-threshold mechano-sensitive C-fibres were not among those DRG to bind soluble NKG2D receptors (Fig. 4). This finding stands in contrast to our transcriptomic analysis, which revealed the potential for *Mult1* (*Ulbp1*) expression by C-LTMRs [41]. There are several possible explanations for this discrepancy: (1) *Mult1* (*Ulbp1*) transcripts may not be functionally expressed as cell surface ligands. (2) Mult1 ligands may not be recognised by soluble NKG2D receptors. (3) Mult1 ligands may not be accessible to receptor binding at the cell surface of C-LTMRs, for which further investigation is required.

### NKG2D as a functional biomarker of nociceptive neuro-immune interaction after injury

High and low-threshold C-fibres are both functionally and transcriptionally distinct [40, 73]. Neuro-immune interactions between either of these two neuronal subsets are therefore likely to have differing pathophysiological consequences for somatosensation. Unmyelinated nociceptors, and the non-peptidergic subset in particular, have been implicated in neuropathic pain in mice after nerve injury or peripheral neuropathy [74–77]. A population of small diameter nociceptive neurons expressing *Mrgprd* (i.e. non-peptidergic) are thought to undergo cell death after traumatic nerve injury [78]. Whether the appearance of functional NKG2D ligands at the soma of these DRG neurons after injury renders them susceptible to immune-mediated killing remains an intriguing possibility.

Aberrant regeneration of unmyelinated sensory afferents after insult or injury has well-documented pathological consequences for somatosensation. Collateral sprouting of unmyelinated high-threshold nociceptors into Meissner corpuscles of denervated territories after nerve injury drives mechanical hypersensitivity [79]. Similarly, re-wiring of Merkel cells by non-peptidergic nociceptors is thought to underly chronic itch in a model of dry skin [80]. Conversely, sprouting is not observed by low threshold Aβ fibres or C fibres [81], which are thought to undergo gain-of-function within adjacent uninjured territories after nerve-injury.

Preferential expression of NKG2D ligands by putative pathological *Mrgprd*+ high-threshold mechanoreceptors [76, 77] as they reinnervate peripheral tissues may help explain the potential for NK cell stimulation to resolve neuropathic mechanical hypersensitivity after partial crush nerve injury [18]: a preclinical model of neuropathic pain in which axons are not restricted from regenerating [82]. Whether other sensory modalities, such as pathological itch, are affected by NK cell-sensory neurons interactions remains to be explored.

### Translation of NK-sensory neuron interactions to humans

We identified transcripts for a number of known NKG2D ligands in published human DRG and hiPSCd sensory neuron datasets. Among the most consistently identified transcript at near single-cell resolution was *ULBP2* (Fig. 5). We additionally identified that human NKG2D receptor-binding to nociceptor-like hiPSCd sensory neurons significantly increases after axon ablation (Fig. 6), indicating the upregulation of cell surface NKG2D ligands after nerve injury. These results echo earlier findings of ULBP expression by dermal nerve fibres in people with fibromyalgia [83], strengthening the potential functional role that NKG2D-ligand interactions may play between NK cells and human nociceptive neurons in peripheral nerve pathology. The identification of *KLRK1* expressed by non-neuronal cells with a gene profile consistent with cytotoxic NK and T lymphocytes within pathological human peripheral nervous tissues (Fig. 5) further reinforces the feasibility of NKG2D receptor-ligand interactions in a clinical context.

Compared to mice, the distinction between subtypes of human nociceptors is less clear. Human DRG show a greater overlap in expression of *CALCA*+ and *P2X3*+, which in mice are markers traditionally associated with peptidergic and non-peptidergic neurons, respectively [84]. There is also higher prevalence of human DRG neurons expressing classical peptidergic markers CGRP and TRPV1 [85]. While the human iPSC differentiation protocol we employed is well-validated [59] it must be emphasised these cells offer only an approximation of human nociceptive DRG neurons [86]. Alternative differentiation protocols, which result in a greater number of TRPV1 and CGRP-positive neurons [62] may provide more translatable findings.

The difference in findings from bulk RNA sequencing datasets from whole tissue and cultured DRG, which had a higher prevalence of *MICA* and *MICB* transcripts, could indicate ligand expression by non-neuronal cells, including lymphocytes and satellite glia, within human DRG [11, 87]. The interaction between NKG2D and its ligands expressed on non-neuronal cells in the context of peripheral nerve injury remains to be explored.

Consistent with previous findings in mice [18], degeneration of hiPSCd sensory axons by primary human NK cells was prevented by an NKG2D blocking antibody (Fig. 6). This effect was observed in the absence of prior axonal injury, which suggests hiPSCd sensory neurons may either retain developmental expression of NKG2D ligands from their hiPSC identity, or that the differentiated neurons exist in a basal ‘stress-like’ state. The enhanced binding of NKG2D we observed to hiPSCd sensory axons after laser axon ablation suggests that axotomy alone is sufficient to induce an injury-like state. Further work is required to validate the characteristics of hiPSCd sensory neurons as an in vitro model of injury-induced neuropathology, ideally in comparison to primary human DRG neurons.

There has been a recent focus on obtaining objective biomarkers to better understand the subjective experience of pain [88]. The search for biochemical biomarkers of nerve injury has typically focussed on easily accessible analytes in the peripheral blood, such as neurofilament light chain [89]. A cell surface-expressed protein biomarker that is found at low levels in ‘healthy’ individuals and upregulated by peripheral nerve injury - as identified here in the form of Raet1e (mouse) and ULBP2 (human) - could pave the way for the identification of nerve damage via non-invasive imaging or radiolabelling techniques.

### Limitations

This study was performed in mice of both sexes on a C57BL/6J background using animals sourced from suppliers at three geographical locations (UK, South Korea and US). While the sex and supplier variables enhance the external validity of our findings, the use of a single genetic background, which is known to express different NKG2D ligands compared to other strains such as Balb/c [33], is a significant limitation. An examination of NKG2D ligands after nerve injury in other strains of mice would strengthen the claims of the evolutionary conservation of this particular neuro-immune interaction.

The small sample size of the single cell PCR experiment (18–20 *Gapdh+ Advillin+* DRG neurons per group) limits interpretation of the minimal detection of *Mult1*. The high proportion of small diameter neurons among those collected also emphasises the sample bias inherent in DRG neuron culture [63].

We focussed our receptor binding analysis on the neurites of DRG neurons in vitro. The purpose of the soma extraction step in our analysis was to avoid the confound of dead/dying cells, which non-specifically attract recombinant protein and antibody binding. We also cannot exclude the possibility of cross-reactivity [90] between the human IgG1 Fc domain of the chimeric receptor protein and Fcγ receptors expressed by mouse DRG neurons [91]. Although we provide evidence for NKG2D ligand expression predominantly at distal neurites, there remains a potential for functional receptor expression at DRG soma. Further work investigating ligand dynamics will help shed light on their subcellar distribution, and whether cytotoxic neuroimmune interactions at the soma may have consequences for neuron viability after nerve injury [78].

## Conclusion

The de novo expression of NKG2D receptor ligands at the cell surface of unmyelinated nerve fibres represent an anatomically restricted marker of peripheral nerve injury. Functional display of ligands for receptors of immune surveillance on pathological nerve fibres is likely to result in reciprocal neuro-immune interactions that influence somatosensation and pain, as well as the local immune microenvironment in injury or disease.

## Methods (abbreviated)

Full methodological details, including genetically altered mouse lines, tamoxifen administration, TRPV1-expressing neuron ablation, microfluidic device preparation, plasmid amplification and purification, RNAscope and RNA sequencing dataset analysis, as well as catalogue numbers of reagents, are available in **Supplementary Material**.

### Ethical approvals

Human NK cells were isolated from the peripheral blood of healthy donors collected by the UK National Health Service (NHS) Blood and Transplant service and distributed by Non-Clinical Issue (NCI) with the approval of the University of Oxford Medical Sciences Interdivisional Research Ethics Committee (MS IDREC) (Reference: R70042/RE002) and stored under a Human Tissue Authority site licence (HTA_12217; Project 00122). Peripheral blood was also collected from healthy donor volunteers after informed consent with the approval of the South Central–Oxford A Research Ethics Committee (14/SC/0280). All nerve injury and capsaicin administration procedures were approved by the Institutional Animal Care and Use Committee (IACUC) at Seoul National University (SNU-121011-1) in Korea and Boston Children’s Hospital (15-04−2928R and 16-01−3080R) in the USA. Tamoxifen dosing in inducible *cre* lines was performed under a UK Home Office Project Licence (P1DBEBAB9). Animals were killed according to Schedule 1 of the UK Home Office (Scientific Procedures) Act (1986).

### Animals

This study is reported in accordance with the RIVER (Reporting In Vitro Experiments Responsibly) recommendations for in vitro experiments [92] and ARRIVE (Animal Research: Reporting of In Vivo Experiments) guidelines for animal experiments [93]. All mice were group-housed in individually ventilated cages with free access to food and water, in humidity and temperature-controlled rooms with a 12 h light-dark cycle (lights on 07.00am), in a pathogen free facility. Male and female C57BL/6J mice were purchased from the Envigo (Inotiv) in the UK, Dae Han Bio Link (Taconic) in Korea, or Jackson Laboratories (Jax) in the US, and were used 8–12 weeks of age. Genetically altered mouse lines were used aged 12–40 weeks of age.

### Peripheral nerve injury

For L5 spinal nerve transection (L5x) injury mice were placed under isoflurane anaesthesia by inhalation (3% induction, maintained 1–2% in 99% O_2_ at 1–2 L/min), the dorsal lumbar region was shaved, treated with an iodine solution (Potadine) and a unilateral incision made parallel to the L6 vertebrate. The L6 transverse process was cut and removed to reveal the L5 spinal nerve, which was cut with fine spring scissors; 1 mm of the nerve was removed to prevent nerve regeneration. For spared nerve injury (SNI), mice were anesthetized with isoflurane (2%–4%) at 9 weeks and SNI surgery performed; the tibial and common peroneal branches of the sciatic nerve were tightly ligated with a silk suture and transected distally, whereas the sural nerve was left intact. No post-surgical analgesia was provided.

### DRG neuron culture

Mouse DRG were rapidly dissected from the spinal column and placed in ice cold Ca^2+^ and Mg^2+^-free Hank’s Buffer Saline Solution supplemented with 20 mM HEPES. Individual DRG were dissected and trimmed of nerve roots and digested 60 min in collagenase A (1 mg/ml) and dispase II (2.4 U/ml) at 37 °C. Additional digestion was carried out for 5–7 min in trypsin (0.25%) before dissociation with fire-polished glassed pipette. Debris was removed by centrifugation for 10 min at 200 g on a layer of bovine serum albumin (BSA) diluted to 15% in DMEM. 10^3^ DRG neurons were plated on 13 mm diameter glass coverslips previously coated with poly-D-lysine (PDL) (10 µg/ml) and laminin (10 µg/ml). DRG were maintained in culture in neurobasal medium with B27 supplement, L-glutamine (1 mM), penicillin (100 U/ml) and streptomycin (100 U/ml) supplemented with nerve growth factor (NGF 2.5 S) at 50 ng/ml at 37 °C, 5% CO_2,_ for up to 3 days prior to immunolabeling.

### Human induced pluripotent stem cell derived (hiPSCd) sensory neuron cultures

hiPSCs from healthy control donors were obtained via the University of Oxford StemBANCC consortium. hiPSC lines were characterised by probe-based karyotyping and confirmed free from mycoplasma. Neurons were seeded either onto 13 mm diameter glass coverslips (approximately 20,000 cells per coverslip), 24-well glass bottom plates (60,000 cells per well), or microfluidic devices (50,000 cells per device) previously coated with poly-D-lysine (PDL) (10 µg/ml) followed by reduced growth-factor Matrigel or Geltrex (LDEV-Free, hESC-Qualified). Neurons were matured in N2 ‘complete’ media: neurobasal medium supplemented with N2, B-27 minus vitamin A, Glutamax and 1x antibiotic-antimycotic (Anti-anti) plus recombinant human β-NGF (rhNGF), NT3, GDNF, and BDNF at 25 ng/ml each in an incubator at 37 °C, 5% CO_2_ for at least 4 weeks (40 days after differentiation), with media changes twice per week.

### hiPSCd-sensory neuron axon ablation for NKG2D receptor labelling

Cryopreserved sensory neuron precursors were thawed and seeded (60,000 cells/well) onto 24-well glass bottom plates coated with PDL (10 µg/ml) and Geltrex and cultured in N2 ‘complete’ media. After 4 weeks the axons of hiPSCd-sensory neurons were ablated in a line in the centre of each well by a 355 nm UV laser at 3% laser power controlled by SysCon software under visual control via a spinning disc confocal microscope. Cultures were maintained up to one week to allow regenerated neurites to be assessed in subsequent experiments.

### NKG2D receptor binding and Immunolabelling

Recombinant murine and human NKG2D receptor proteins were diluted to 2 µg/ml in neurobasal media containing 1% BSA and applied to live DRG neurons on coverslips for 1 h at 37 °C. Cells were gently washed three times with PBS and fixed with 4% PFA in PBS for 30 min at room temperature (RT) before washing with PBS followed by two washed with DMEM containing 20 mM HEPES (DMEM/HEPES). To immunolabel Fc-conjugated receptor proteins, coverslips were treated with either Alexa 488-conjugated goat anti-human IgG (1:750) (RRID: AB_2534080) or Cy3-conjugated goat anti-human IgG (1:750) (RRID: AB_2810895) in DMEM/HEPES and 1% BSA for 1 h at RT.

### Heterologous expression of mouse Raet1e in HEK293T cells

HEK293T cells were transfected with plasmid DNA using polyethylenimine (PEI). Tranfected cells were treated with recombinant human NKG2D-Fc chimeric receptor or Fc-control (1 µg/ml) in DMEM including 1% BSA, for 1 h at 37 °C, 5% CO2. Cells were then washed 3x PBS and fixed with PFA (4% diluted in 0.01 M PBS) for 20–30 min before immunolabelling for human IgG.

### Confocal imaging

Immuno-labelled cultures were imaged on a laser scanning confocal microscope (LSM700, Zeiss) fitted with 3 laser lines (405, 488, and 546 nm). Individual neurons were first identified in the 405 nm laser channels corresponding to B-tubulin immunolabelling. For high-throughput quantification of NKG2D immunolabelling in sensory neuron cultures over time, coverslips were imaged on a spinning disc confocal microscope (iXplore SpinSR10, Olympus) with 4 Laser lines (405, 488, 561 and 640 nm) fitted to an inverted microscope (IX83, Olympus) using stage navigator with Z-Drift Compensation for automated, systematic sampling. Identical laser and acquisition settings were maintained throughout.

### Confocal image analysis

For manual counting of the number of NKG2D-binding neurons in the different subpopulations, individual β-tubIII + neurons in a given field of view were manually assigned as either NKG2D-positive or negative while blinded to the expression of TdTomato. Images where neuronal densities were too high for identification of individual neurons (as assessed by the observer) were excluded from analysis. For automated quantification of NKG2D labelling of murine DRG neurites, a bespoke analysis pipeline was executed using Fiji, in combination with a method for soma detection (Directional Ratio) run via a script in MatLab [94]. For automated quantification of NKG2D/Fc binding to human iPSC-derived sensory neurons, maximum intensity projection (MIP) images of BtubIII+ channel and the respective neurite-segmented images were analysed. The fragmentation of human iPSC-sensory neuron was quantified using semi-automated method. The fragmentation (%) = Area 1/Area 2 × 100. A description of the full analysis pipeline and links to Macro scripts held on GitHub can be found in **Supplementary Material.**

### Human natural killer (NK) cell isolation and stimulation

Human NK cells were isolated from the peripheral blood of three healthy volunteers, as well as leukocyte cones from three anonymous volunteer blood donors supplied by UK National Health Service Blood and Transplant (NHSBT) Non-Clinical Issue (NCI) service.

Peripheral blood mononuclear cells (PBMC) were separated by lympholyte Human Cell Separation Media in 50 ml SepMate tubes and centrifuged 22 min at 800 g at room temperature (RT) with no brake.

100 × 10^6^ PBMCs were resuspended in 2 ml of MACS buffer. NK cells were enriched by negative selection using EasySep^™^ Human NK Cell Isolation Kit, according to the manufacturer’s instructions. The isolated NK cells were suspended in cryopreservation media (50% RPMI, 40% FBS, 10% DMSO) and frozen using a controlled-rate alcohol-free cell freezing container (CoolCell, Corning) at −80 °C before being transferred to vapour phase nitrogen for long-term cryostorage.

For NK cell stimulation, vials of cryopreserved NK cells were rapidly thawed in a water bath at 37 °C and washed in RPMI including 10% FBS supplemented with DNase I (125 U/ml) followed by centrifugation at 400 g, 10 min, RT. Cells were counted and seeded at 2 × 10^6^ cells per ml in RPMI plus 10% FBS in 96 well U-bottom plates supplemented with recombinant human IL-2 (10^3^ U/ml; 100 ng/ml equivalent) and cultured for 2 days at 37 °C, 5% CO_2_.

### Flow cytometry

Whole PBMC, NK-depleted fraction and purified human NK cells (5 × 10^5^ cells per 100 µl) were suspended in FACS buffer (PBS + 2% FBS) and blocked with normal human serum (NHS, 10%) for 15 min on ice. Cells were treated with fluorescently conjugated antibodies and incubated 40 min at 4 °C protected from light. Cells were washed 2x FACS buffer with centrifugation 500 g, 5 min. The fluorescent DNA intercalator 7-aminoactinomycin D (7-AAD) (1:100) was added to all samples (except single stain controls) prior to cytometry. Samples underwent flow cytometry on an LSRII Special Order Research Product (SORP) digital cell analyser equipped with a Violet (405 nm, 100mW), Blue (adjustable 488 nm, 80mW), Green (532 nm, 150mW) and Red (642 nm, 40mW) lasers.

NKG2D receptor expression levels, as well as production of perforin and granzyme B by NK cells were analysed by full spectrum flow cytometry (Aurora 5 laser Spectral Flow Cytometer, Cytek) equipped with UV (355 nm, 20mW), violet (405 nm, 100mW), blue (488 nm, 50mW), 50mW yellow-green & red (638 nm, 80mW) lasers. Flow cytometry data are available from Zenodo [95].

### NKG2D blocking assay

For antibody blockade of human NKG2D function in vitro, NK cells were incubated with 50 µg/ml of LEAF purified anti-human CD314 (NKG2D) (clone 1D11) (RRID: AB_2561488) or LEAF purified human IgG1 isotype control (Clone QA16A12) (RRID: AB_2927629) for 30 min at room temperature (2.5 × 10^6^ NK cells per ml) before addition to target cells (hiPSCd sensory neurons) in the neurite compartment of microfluidic devices 4 weeks after hiPSCd neuronal precursor seeding. NK cells were seeded at different densities (50 × 10^3^, 100 × 10^3^, 200 × 10^3^) and cultured for 4 h.

Cells were then gently washed three times by HBSS, followed by fixation by 4% PFA for 30 min. Fixed cells were washed three times by HBSS and blocked and permeabilized by PBS containing 5% normal serum and 0.1% Triton-X100 for 1 h at room temperature. Then cells were incubated with primary antibody diluted in PBS containing 0.5% normal serum and 0.01% Triton-X100 overnight at 4 °C. Cells were washed three times by PBS and incubated with DAPI and secondary antibody diluted in PBS containing 0.5% normal serum and 0.01% Triton-X100 for 1 h at room temperature. Then cells were washed three times by PBS and the reservoirs of microfluidics were filled with PBS and covered with a coverslip.

### RNA extraction from mouse DRG cultures

After various time points in culture (day 1, 2, and 3), coverslips were gently washed with PBS before lysis with a phenol-containing lysis buffer (Tripure, Roche). Lysates were transferred to 1.5 mL samples tubes, snap-frozen on dry ice, and stored at −80 °C until RNA extraction using filter column purification.

### Reverse transcription

Reverse transcription for quantitative PCR was performed using Moloney Murine Leukemia Virus (M-MLV) reverse transcriptase kit with oligo(dT) primers.

### Quantitative polymerase chain reaction (qPCR)

Quantitative gene expression in DRG cell cultures was performed on cDNA (0.33 ul per tube per sample from original reverse transcription) using a SYBR Green PCR Master Mix (Roche) and pairs of target-specific primers (500 nM each) in a 10 µl sample volume on white skirted 384-well PCR plate on a LightCycler 480 (Roche). ΔΔCt values were normalised to day 0 DRG to present as fold change in transcripts at each time point for each culture, or to contralateral DRG for nerve injury experiments.

### Gel electrophoresis

qPCR reaction products (10 µL) were run on 1.5% agarose gel stained with GelRed at 100 V for 30 min with 100 bp ladder. The gels were imaged using a UV illuminator (NuGenius, Syngene).

### Single cell DRG collection, reverse transcription and nested PCR

Adult male C57BL/6 mouse DRG neurons were cultured overnight in Neurobasal media supplemented with NGF (50 ng/ml) on coverslips previously coated with poly-D-lysine (10 µg/ml) and laminin (10 µg/ml). Single DRG neurons were collected into borosilicate glass micropipettes containing a reverse transcription buffer by applying gentle negative pressure under visual control and were immediately ejected into a collection buffer containing dNTP, oligo(dT) and random hexamers. PCR was performed with nested primers using Platinum *Taq* DNA polymerase (Invitrogen) according to the manufacturer’s instructions. All procedures were carried out in a strict nuclease-free environment.

### Study design and statistics

For in vivo experiments, the biological unit of interest is the animal (i.e. number of mice). For murine cell culture experiments the biological unit of interest is either the animal (i.e. number of cultures for RNA analysis) or the cell (i.e. number of DRG neurons for immunohistochemical analysis), unless otherwise defined; where individual neurons were not defined (i.e. during automated image analysis), the experimental unit was a region of interest. Mice of both sexes were used unless otherwise indicated. For human cell culture experiments, the biological unit of interest is the individual donor. Experimental units are defined in figure legends. Microfluidic devices without NK cells were excluded from data acquisition because of the low density of axons. Where multiple observations were made of RNA levels or immunohistochemical signals in vitro, the various treatments (including controls) were applied to replicate experimental units (i.e. cell culture well) derived from each biological unit.

Each replicate cell culture contained the full suite of experimental and control samples. Assignment of cell culture wells to different time points or treatment with different reagents (i.e. recombinant proteins or cells) was not randomised. Counting of neurons was performed on images offline after manual image acquisition. DRG neuron culture from a single male mouse for resulted in low levels of extracted RNA (< 5 µg/ml) at days 1, 2 and 3 in vitro (Fig. 1B), therefore data from this animal was excluded from the figure as well as any further analysis.

For in vivo experiments, sample sizes (i.e. number of animals) were based on previous publication [18]. In situ hybridization (RNA scope) experiments were performed based on availability of mouse tissues cryopreserved as a legacy of previous studies in the lab. In vitro experiments were exploratory and therefore sample size was not calculated *a prior*. All the graphs, calculations and statistical analyses were performed using GraphPad Prism software 10.0. Data points represent mean values of replicate measurements with standard error of the mean, unless otherwise stated. Pair-wise comparisons of normally distributed data were analysed using a two-tailed Student’s t test. Pair-wise comparisons of non-normally distributed data were analysed using a two-tailed Mann-Whitney U test. Where the experimental unit is a single image (i.e. when calculating receptor particle density) data were compared as cumulative distributions using the Kolmogorov-Smirnov test and presented as violin plots showing median and quartile ranges. Alpha = 0.05. P-values were corrected for multiple comparisons. All data points were included in the data analysis except for the excluded data points described above.

## Supplementary materials

Supplementary material 1: Supplementary Video 1.

Supplementary material 2: Supplementary Methods, Figures, Tables and References.

Supplementary material 3: ARRIVE Guidelines 2.0: Author Checklist.

## Data Availability

All published datasets used in this study are cited in the results and figure legends. Flow cytometry data are available from Zenodo. Other summary data are available upon reasonable request to the Corresponding authors.

## Abbreviations

MRGPRD: Mas-related G-protein coupled receptor member D
ATF3: Activating Transcription Factor 3
B2m: Beta-2 microglobulin
C-LTMRs: C-low threshold mechanoreceptors
CGRP: calcitonin gene related peptide
DRG: Dorsal root ganglion
H60: Histocompatibility 60
hiPSCd: Human induced pluripotent stem cell-derived
IB4: Isolectin B4
IL-2: Interleukin-2
L5x: 5th lumbar spinal nerve transection
MICA/B: MHC class I polypeptide-related sequence
MULT1: Murine UL16-binding protein-like transcript 1
NGF: Nerve growth factor
NK: Natural killer
NKG2D: Natural killer group 2D
RAET1: Retinoic acid early transcript 1
RTx: Resiniferatoxin
SNI: Spared nerve injury
TH: Tyrosine hydroxylase
TRKA (NRTK1): Neurotrophic Receptor Tyrosine Kinase 1
TRPV1: Transient Receptor Potential Vanilloid 1
ULBP: UL16-binding protein
