# Supplementary figures and images for: NKG2D receptor ligands are cell surface biomarkers for injured murine and human nociceptive sensory neurons
Source: J Neuroinflammation. 2025 Dec 29;23:42. doi: 10.1186/s12974-025-03675-1 (PMC12853809; doi:10.1186/s12974-025-03675-1)

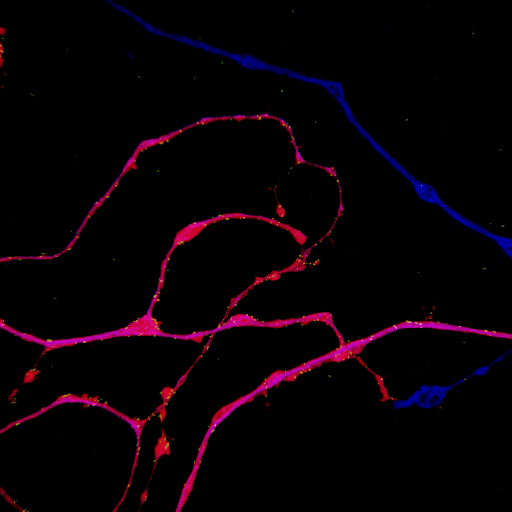

Supplement: Supplementary file 1 — Supplementary material 1: Supplementary Video 1. [file 12974_2025_3675_MOESM1_ESM.gif]
