# Supplementary material for: NKG2D receptor ligands are cell surface biomarkers for injured murine and human nociceptive sensory neurons
Source: J Neuroinflammation. 2025 Dec 29;23:42. doi: 10.1186/s12974-025-03675-1 (PMC12853809; doi:10.1186/s12974-025-03675-1)
Supplement: Supplementary file 2 — Supplementary material 2: Supplementary Methods, Figures, Tables and References. [file 12974_2025_3675_MOESM2_ESM.docx]

# Title: NKG2D receptor ligands are cell surface biomarkers for injured murine and human nociceptive sensory neurons

# Authors

Shuaiwei Wang^1^, Allison M Barry^1,2^, Yoon Kyung Lee^3^, Naomi Young^1^, Sang Wook Shim^4^, Xinying Wang^1^, Hyeongcheol Kim^5,11^, Laura Stirling-Barros^6^, Rafael González-Cano^7,8^, Michael Costigan^8^, Georgios Baskozos^1^, Simon Rinaldi^1^, David LH Bennett^1^, Seog Bae Oh^3,9,10#^, Alexander J Davies^1#^

**Affiliations**

1. Nuffield Department of Clinical Neurosciences, University of Oxford, John Radcliffe Hospital, Oxford, OX3 9DU, United Kingdom
2. Center for Advanced Pain Studies, School of Behavioral and Brain Sciences, Department of Neuroscience, University of Texas at Dallas, Richardson, TX 75080, USA
3. Interdisciplinary Program in Neuroscience, Seoul National University, Seoul 08826, Republic of Korea
4. Department of Brain and Cognitive Sciences, Seoul National University, Seoul 08826, Republic of Korea
5. Synteraction Lab, National University of Singapore, School of Computing, COM1, 13, Computing Dr, Singapore, 117417, Singapore
6. Medical Sciences Division, University of Oxford, John Radcliffe Hospital, Oxford, OX3 9DU, United Kingdom
7. Department of Pharmacology, Faculty of Medicine and Biomedical Research Center (Neurosciences Institute), Biosanitary Research Institute, University of Granada, Granada, Armilla, 18100, Spain.
8. The Department of Anesthesiology, Critical Care and Pain Medicine, Boston Children's Hospital, Harvard Medical School, Boston, MA, United States.
9. Department of Neurobiology and Physiology, School of Dentistry, and Dental Research Institute, Seoul National University, Seoul 03080, Republic of Korea
10. ADA Forsyth Institute, Somerville, MA, USA
11. InnoCORE PRISM-AI Research Group, Korea Advanced Institute of Science and Technology, Daejeon, Republic of Korea

^#^Corresponding authors:

[alexander.davies@ndcn.ox.ac.uk](mailto:alexander.davies@ndcn.ox.ac.uk)

[odolbae@snu.ac.kr](mailto:odolbae@snu.ac.kr)

Table of Contents

[Supplementary Methods 4](#_Toc217385506)

[Ethical approvals 4](#_Toc217385507)

[Animals 4](#_Toc217385508)

[Tamoxifen administration 5](#_Toc217385509)

[Peripheral nerve injury 5](#_Toc217385510)

[TRPV1-expressing neuron ablation 5](#_Toc217385511)

[DRG neuron culture 6](#_Toc217385512)

[Microfluidic device preparation 6](#_Toc217385513)

[Human induced pluripotent stem cell derived (hiPSCd) sensory neuron cultures 7](#_Toc217385514)

[hiPSCd-sensory neuron axon ablation for NKG2D receptor labelling 8](#_Toc217385515)

[NKG2D receptor binding and immunolabelling 8](#_Toc217385516)

[Plasmid amplification and purification 9](#_Toc217385517)

[Heterologous expression of mouse Raet1e in HEK293T cells 10](#_Toc217385518)

[Confocal imaging 10](#_Toc217385519)

[Confocal image analysis 11](#_Toc217385520)

[Human natural killer (NK) cell isolation and stimulation 12](#_Toc217385521)

[Flow cytometry 13](#_Toc217385522)

[NKG2D blocking assay 14](#_Toc217385523)

[Quantitative PCR 15](#_Toc217385524)

[RNA extraction from mouse DRG cultures 15](#_Toc217385525)

[RNA extraction from mouse DRG tissues 15](#_Toc217385526)

[Reverse transcription 16](#_Toc217385527)

[Quantitative polymerase chain reaction (qPCR) 16](#_Toc217385528)

[Gel electrophoresis 17](#_Toc217385529)

[Single cell DRG collection, reverse transcription and nested PCR 17](#_Toc217385530)

[In situ hybridization (RNAscope) 18](#_Toc217385531)

[RNA sequencing dataset analysis 18](#_Toc217385532)

[Study design and statistics 20](#_Toc217385533)

[Supplementary Figures 21](#_Toc217385534)

[Supplementary Figure 1. Regulation of NK cell stimulatory and inhibitory ligands in DRG by L5x injury. 22](#_Toc217385535)

[Supplementary Figure 2. In situ hybridisation (RNAscope) for *Raet1* mRNA in lumbar DRG of mice after nerve injury. 23](#_Toc217385536)

[Supplementary Figure 3. Single cell reverse-transcription PCR of mRNA transcripts in acutely isolated adult mouse DRG. 25](#_Toc217385537)

[Supplementary Figure 4. Development of a live cell-based assay for mouse NKG2D ligands. 26](#_Toc217385538)

[Supplementary Figure 5. Examples of NKG2D labelling in sensory neurons from Calca-TdTom reporter line. 27](#_Toc217385539)

[Supplementary Figure 6. NKG2D labelling in sensory neurons from Thy1-YFP and TRPV1- TdTom reporter line. 28](#_Toc217385540)

[Supplementary Figure 7. Analysis of human DRG bulk and single-cell RNA sequencing datasets. 29](#_Toc217385541)

[Supplementary Figure 8. Systematic analysis of receptor binding to hiPSCd sensory neuron axons after laser ablation. 30](#_Toc217385542)

[Supplementary Figure 9. Purity and stimulation of human NK cells. 32](#_Toc217385543)

[Supplementary Video 1. Super-resolution image of NKG2D receptors bound to DRG neurites in vitro. 33](#_Toc217385544)

[Supplementary Tables 34](#_Toc217385545)

[Supplementary Table 1. Genetically altered mouse lines. 34](#_Toc217385546)

[Supplementary Table 2. iPSC donor lines 35](#_Toc217385547)

[Supplementary Table 3. Antibodies for immunofluorescence staining 36](#_Toc217385548)

[Supplementary Table 4. Plasmid details 37](#_Toc217385549)

[Supplementary Table 5. Antibodies for flow cytometry 38](#_Toc217385550)

[Supplementary Table 6. Primer sequences for qPCR 39](#_Toc217385551)

[Supplementary Table 7. Primer sequences for single cell nested PCR 40](#_Toc217385552)

[Supplementary References 41](#_Toc217385553)

# Supplementary Methods

## Ethical approvals

Human NK cells were isolated from the peripheral blood of healthy donors collected by the UK National Health Service (NHS) Blood and Transplant service and distributed by Non-Clinical Issue (NCI) with the approval of the University of Oxford Medical Sciences Interdivisional Research Ethics Committee (MS IDREC) (Reference: R70042/RE002) and stored under a Human Tissue Authority site licence (HTA_12217; Project 00122). Peripheral blood was also collected from healthy donor volunteers after informed consent with the approval of the South Central–Oxford A Research Ethics Committee (14/SC/0280). All nerve injury and capsaicin administration procedures were approved by the Institutional Animal Care and Use Committee (IACUC) at Seoul National University (SNU-121011-1) in Korea and Boston Children's Hospital (15-04-2928R and 16-01-3080R) in the USA. Tamoxifen dosing in inducible *cre* lines was performed under a UK Home Office Project Licence (P1DBEBAB9). Animals were killed according to Schedule 1 of the UK Home Office (Scientific Procedures) Act (1986).

## Animals

All mice were group-housed in individually ventilated cages with free access to food and water, in humidity and temperature-controlled rooms with a 12hr light-dark cycle (lights on 07.00am), in a pathogen free facility. For collecting DRG tissues, all the mice were culled using Schedule 1 by exposure to a rising concentration of carbon dioxide followed by cervical dislocation. *Nav1.8^cre^* (*Scn10a^tm2(cre)Jnw^*) mice [1] were kindly gifted by Prof. John Wood, *Calca^creERT2^* (*Calca^tm1.1(cre/ERT2)Ptch^*) [2] were kindly gifted by Prof. Pao-Tien Chuang. *Mrgprd^creERT2^* mice (*Mrgprd*^tm1.1(cre/ERT2)Wql/J^) [3] (RRID:IMSR_JAX:031286) and *Th^creERT2^* mice (*Th*^tm1.1(cre/ERT2)Ddg/J^) [4] (RRID:IMSR_JAX:025614) were kindly gifted by Prof. David Ginty, Thy-1 YFP-16 (*B6;Cg-Tg(Thy1)-^(YFP)16Jrs/J^*) mice [5] (RRID:IMSR_JAX:003709) were kindly gifted by Prof. Pilhan Kim. *Trpv1^cre^* (*Trpv1^tm1(cre)Bbm^/J*) [6] (RRID: IMSR_JAX:017769), *R26R^DTA^* (*B6;129-Gt(ROSA)26Sor^tm1(DTA)Mrc^/J*) [7] (RRID:IMSR_JAX:010527) and Ai14 (*B6.Cg-Gt(ROSA)26Sor^tm14(CAG-tdTomato)Hze^/J*) [8] (RRID:IMSR_JAX:007914) mice were purchased from Jackson Labs (**Supp. Table 1**). Cre-dependent reporter lines were maintained with heterozygote inbreeding. Heterozygote cre-dependent reporter mice were crossed with *DTA* or Ai14 ‘TdTomato’ reporter mice generate conditionally ablate or fluorescently label of selected sensory neuronal lines, respectively. Double-heterozygous mice were used for experiments. Male and female C57BL/6J mice were purchased from the Envigo (Inotiv) in the UK, Dae Han Bio Link (Taconic) in Korea, or Jackson Laboratories (Jax) in the US, and were used 8-12 weeks of age. Transgenic mice were used aged 12-40 weeks of age. Owing to unpredictable breeding patterns, and to maximise the use of tissues, *Mrgprd^creERT2^* mice used in this study had received spared nerve injury to the left sciatic nerve 30 days prior to cell culture for other experiments; bilateral L3-5 DRG were therefore excluded from the cell culture used for receptor-binding analysis.

## Tamoxifen administration

# All cre/ERT2 expressing mice were dosed 5x (daily) with 75 mg/kg Tamoxifen (Sigma-Aldrich) in adulthood (8+ weeks), as reported previously [9]. Tamoxifen stocks were freshly prepared by dissolving at 20 mg/ml in corn oil via sonification. All animals were dosed i.p. and health statuses were monitored daily for the duration of the dosing regimen.

## Peripheral nerve injury

For L5 spinal nerve transection (L5x) injury mice were placed under isoflurane anaesthesia by inhalation (3% induction, maintained 1-2% in 99% O_2_ at 1-2 L/min), the dorsal lumbar region was shaved, treated with an iodine solution (Potadine) and a unilateral incision made parallel to the L6 vertebrate. Under a x20 dissection microscope illuminated by a cold light source the musculature was parted by blunt forceps dissection to reveal the L6 transverse process, which was then cut and removed. The L5 spinal nerve, which runs immediately below the L6 process, was carefully freed of connective tissue and cut with fine spring scissors; 1 mm of the nerve was removed to prevent nerve regeneration. The wound as irrigated with sterile saline and closed in two layers with 6-0 silk sutures (Ailee, Korea) and 9mm skin clips (MikRon Precision, CA, USA) [10]. For spared nerve injury (SNI), mice were anesthetized with isoflurane (2%–4%) at 9 weeks and SNI surgery performed; the tibial and common peroneal branches of the sciatic nerve were tightly ligated with a silk suture and transected distally, whereas the sural nerve was left intact [11]. Mice were placed in a warm, darkened cage to recover from surgery and monitored daily for signs of malaise, piloerection, weight loss or autotomy. No post-surgical analgesia was provided.

## TRPV1-expressing neuron ablation

TRPV1-expressing neurons were ablated with resiniferatoxin (RTx) as previously described [12]. Briefly, under isoflurane anaesthesia 3-4 week old male mice were injected i.p. with RTx at 50 µg/kg and 150 µg/kg or equivalent volume of vehicle (10% ethanol, 10% tween-80 in sterile saline) over two consecutive days. Mice were maintained under anaesthesia (0.5% isoflurane in 100% O_2_ at 1-2 L/min) for 2 h on a warm-pad before recovery in a warmed cage. Four weeks after RTx or vehicle treatment mice were tested for TRPV1+ neuron depletion by applying 10 µl capsaicin (0.01% in sterile saline) to one eye and counting the number of unilateral eye wipes in a 1 min period. Video analysis was performed by an observer blinded to the treatment of the mice.

## DRG neuron culture

Dorsal root ganglion (DRG) neurons were cultured as previously described [10,13]. Mice were killed by rising concentration of carbon dioxide and death confirmed by cervical dislocation. The spinal column was rapidly dissected and placed in ice cold Ca^2+^ and Mg^2+^-free Hank’s Buffer Saline Solution (Gibco, 14065-049) supplemented with 20mM HEPES. Individual DRG were dissected and trimmed of nerve roots and digested 60 min in collagenase A (1 mg/ml) (Cat. 10103578001, Roche) and dispase II (2.4U/ml) (Cat. 04942078001, Roche) at 37°C. Additional digestion was carried out for 5-7 min in trypsin (0.25%) in Dubellco’s Modified Eagle Medium (DMEM) (Cat. 41965-039, Gibco) and stopped with a trypsin inhibitor (2.5 mg/ml) (Cat. T9003, Sigma) in PBS followed by washing in DMEM containing 10% foetal bovine serum (FBS) (Life Technologies). DRG were dissociated by trituration with a fire-polished glassed pipette in DMEM containing DNase I (125 U/ml) (Cat. 58409700, Roche) and centrifuged 10 min at 200 g on a layer of bovine serum albumin (BSA) (Cat. A7248, Sigma) diluted to 15% in DMEM, before re-suspension in neurobasal medium (Cat. 12348017, Life Technologies) with B27 supplement (Cat. 17504-004, Life Technologies), L-glutamine (1 mM), penicillin (100 U/ml) and streptomycin (100 U/ml) (Cat. 15140-122, Life Technologies) supplemented with nerve growth factor (NGF 2.5S) at 50 ng/ml. 10^3^ DRG neurons were plated on 13 mm diameter glass coverslips previously coated with poly-D-lysine (PDL) (10 μg/ml) (Cat. P6406, Sigma) and laminin (10 μg/ml) (Cat. L2020, Sigma) and maintained in culture at 37°C, 5% CO_2,_ for up to 3 days prior to immunolabeling.

## Microfluidic device preparation

Microfluidic devices created from polydimethylsiloxane (PDMS) (Cat. 63416.5S, Silicone Sylgard 184, Scientific Laboratory Supplies Limited). Slygard 184 silicon base (density approximately 1g/ml) and curing agent were mixed in a 10:1 ratio. The mixture was rested for 15 min to remove bubbles before pouring into resin master moulds, which were placed in oven for at least one hour at 60-65°C to set. After removing from the resin master moulds, PDMS devices were trimmed and four reservoirs were cut using an 8 mm diameter skin punch biopsy tool (Cat. BP-80F, Selles Medical Ltd). Two of the reservoirs on the neurite side were enlarged to make a single continuous reservoir. The length of microfluidic channels was 150 µm. The trimmed PDMS devices were adhered to low profile glass-bottom dishes (Cat. HBST-5040, Willco wells) by pre-exposure in a low-pressure plasma cleaner (Femto, Diener Electronic GmbH) 2x 30 s at 35% power. Approximately 150 µl sterile distilled water was immediately added in one of the smaller reservoirs to maintain patency of the microfluidic channels, and devices were kept at 4 °C before use.

## Human induced pluripotent stem cell derived (hiPSCd) sensory neuron cultures

hiPSCs from healthy control donors were obtained via the University of Oxford StemBANCC consortium (**Supp. Table 2**). hiPSC lines were characterised by probe-based karyotyping (Karyostat Assay, Applied Biosystems) and confirmed free from mycoplasma (MycoStrip, InvivoGen). hiPSC were maintained in 6-well culture plates coated with hESC-qualified LDEV-free Matrigel (Cat. 354277, Corning) with daily changes of mTeSR1 media (Cat. 85850, StemCell Technologies). Cells were routinely passaged by EDTA dissociation (500 µM in PBS, 5 min) when cells became 70-80% confluent. hiPSCs were differentiated into sensory neurons in 6 well plates using a combination of small-molecule dual SMAD (mothers against decapentaplegic family transcription factor) inhibitors SB431542 (10 µM) (Cat. 1614, Tocris) and LDN-193189 (100 nM) (Cat. 6053, Tocris) and Wnt pathway activators CHIR99021 (3 µM) (Cat. 4423, Tocris), SU5402 (10 µM) (Cat. SML0443, Sigma), and DAPT (10 µM) (Cat. 2634, Tocris) [14,15]. Briefly, hiPSC media was changed to Mouse Fibroblast-conditioned media (MEF) (Cat. AR005, R&D Systems) supplemented with Fibroblast growth factor (FGF)-2 (10 ng/ml) for one day (Day -1). The next day (Day 0) differentiation began with the addition of a series of small molecule inhibitors added to Knock-out Serum Replacement (KSR) media: Knockout DMEM (Cat. 10829018, life Technologies) containing 15% knockout-serum replacement (Cat 10828010, Life Technologies), 1% Glutamax, 1% non-essential amino acids, 1% antibiotic/antimycotic, and β-mercaptoethanol (100 μM). Media was changed daily (2 ml per well), during which time KSR media was gradually replaced with N2 ‘complete’ media (neurobasal medium supplemented with N2 (Cat. 17502-048), B-27 minus vitamin A (Cat. 12587-010, Gibco), Glutamax (Cat. 35050-038, Gibco) and 1x antibiotic-antimycotic (Anti-anti) (Cat. 15240-062, Gibco) plus recombinant human β-NGF (rhNGF) (Cat. 450-01, Peprotech), NT3 (Cat. 450-03, Peprotech), GDNF (Cat. 450-10, Peprotech), and BDNF (Cat. PHC7074, Life Technologies) at 25 ng/ml each), according to published protocols [15,16]. On day 11 or 12 of differentiation, cells were washed with 1 ml per well of PBS, followed by knockout DMEM containing DNase I (100 U/ml) and incubated 5 min at 37°C, 5% CO_2_. The knockout DMEM was then replaced with 1 ml TrypLE dissociation reagent and incubated for a further 5 min at 37°C, 5% CO_2_ before dissociation with fire-polished glass pipette. Cells were transferred to PBS in 15 ml tubes and centrifuged 5 min, 500 g to pellet. Supernatant was removed and cells were resuspended in N2 ‘complete’ media supplemented with CHIR99021 (3 µM) and Rho kinase inhibitor, Y-27632 (10 µM) (Cat. 1254, Tocris). Cells were counted, suspension at 4x10^6^ cells per ml in N2 completed neuronal media (above), diluted 1:1 with chilled 2X freeze media (20% DMSO, 80% charcoal-stripped FBS) and immediately transfer cell suspension to cryovials (2x10^6^ cells per ml per vial).

Thawed precursor neurons were seeded either onto 13 mm diameter glass coverslips (approximately 20,000 cells per coverslip), 24-well glass bottom plates (60,000 cells per well) (Cat. 662892, Greiner-One Bio), or microfluidic devices (50,000 cells per device) previously coated with poly-D-lysine (PDL) (10 μg/ml) followed by reduced growth-factor Matrigel (Cat. 354277, Corning) or Geltrex (LDEV-Free, hESC-Qualified) (Cat. A1413302, ThermoFisher). Sensory neuron precursors were maintained in N2 ‘complete’ media supplemented with CHIR99021 (3 µM) and Rho kinase inhibitor, Y-27632 (10 µM) for the first two days after seeding, after which the neuronal media was supplemented with cytosine arabinoside (Ara-C) (0.5-1 µM) (Cat. C1768, Sigma) to remove proliferating, non-neuronal cells. Neurons were matured in N2 ‘complete’ media in an incubator at 37°C, 5% CO_2_ for at least 4 weeks (40 days after differentiation), with media changes twice per week.

## hiPSCd-sensory neuron axon ablation for NKG2D receptor labelling

Cryopreserved sensory neuron precursors were thawed and seeded (60,000 cells/well) onto 24-well glass bottom plates (Cat. 662892, Greiner Bio-One Ltd) coated with PDL (10 µg/ml) and Geltrex and cultured in N2 'complete' media. After 4 weeks the axons of hiPSCd-sensory neurons were ablated in a line in the centre of each well by a 355 nm UV laser (Rapp OptoElectronic, model: DPSL-355/42/CLS2) 3% laser power controlled by SysCon software under visual control via a spinning disc confocal microscope (iXplore SpinSR10, Olympus). Cultures were maintained up to one week to allow regenerated neurites to be assessed in subsequent experiments.

## NKG2D receptor binding and immunolabelling

Recombinant murine NKG2D-Fc chimeric receptor (Cat. 139-NK, R&D Systems; lot numbers: FRP0218081, FRP0222021, FRP0223061, FRP0224041), recombinant human NKG2D-Fc chimeric receptor (Cat: 1299-NK, R&D Systems; lot number: FVV0618021) and recombinant human IgG1 Fc control (Cat. 110-HG, R&D Systems, lot number: EAX0619041) were prepared by suspension in distilled water at 1 mg/ml for 15 min at room temperature (RT) and further diluted to 100 µg/ml in phosphate buffered saline (PBS) containing 0.1% BSA for storage at -80°C. Some lot-lot variation was observed with murine NKG2D-Fc proteins; lot number FRP0220051 was found in trials to bind poorly to DRG neuron cultures and was therefore not used for experiments. Receptor proteins were diluted to 2 µg/ml in neurobasal media containing 1% BSA and applied to live DRG neurons on coverslips for 1h at 37°C. Cells were gently washed three times with PBS and fixed with 4% PFA in PBS for 30 min at room temperature (RT) before washing with PBS followed by two washed with DMEM containing 20 mM HEPES (DMEM/HEPES). To immunolabel Fc-conjugated receptor proteins, coverslips were treated with either Alexa 488-conjugated goat anti-human IgG (1:750) (Cat. A-11013, Thermo Fisher Scientific, RRID: AB_2534080) or Cy3-conjugated goat anti-human IgG (1:750) (Cat. 109-165-170, RRID: AB_2810895) in DMEM/HEPES and 1% BSA for 1h at RT. Coverslips were washed with DMEM/HEPES followed by PBS. Cells were blocked and permeabilised by incubation with 5% normal goat serum (NGS) and 0.1% triton-X in PBS for 1h at RT and incubated with rabbit anti-β-tubulin III (1:2000) (Cat. T2200, Sigma-Aldrich; RRID: AB_262133) overnight at 4°C. The next day, after washing in PBS coverslips were treated with either Alexa-546 conjugated goat anti-rabbit IgG (1:1000) (Cat. A-11035, Thermo Fisher Scientific, RRID: AB_2534093), Pacific Blue-conjugated goat anti-rabbit IgG (1:1000) (Cat. P-10994, Thermo Fisher Scientific, RRID: AB_2539814), Alexa Fluor 647 conjugated donkey anti-rabbit IgG (1:1000) (Cat. A-31573, Thermo Fisher Scientific, RRID: AB_2536183) or Alexa Fluor 647 conjugated goat anti-rabbit IgG (1:1000) (Cat. A-21244, Thermo Fisher Scientific, RRID: AB_2535812) for 1h at RT (see **Supp. Table 3**). Coverslips were washed in PBS, mounted on glass slides with antifade mounting medium (Vectorshield Plus, Cat. H-1000, Vector Laboratories) and stored at -20°C until imaging; microfluidic devices were flooded with PBS and stored at 4°C.

## Plasmid amplification and purification

Commercial plasmids containing gene inserts encoding murine RAE-1-ε (untagged) or green fluorescent protein (GFP) control under the control of the CMV promoter (see **Supp. Table 4**) were transferred to competent *E. coli* (NEB® Stable, Cat. c3040i, New England Biolabs) by heat-shock treatment. Briefly, 100 ng/μl plasmid DNA was added to 15 ul *E. coli, i*ncubated on ice for 30 minutes, followed by heat-shock at 42°C for 30 s, and immediately returned to ice for a further 5 min. SOC outgrowth media (250 μl) (Cat. B9020S, New England Biolabs) was added to the plasmid/*E. coli* mixture and incubated at 37°C with shaking (250 rpm) for 1 h. 10 to 20 μl of the starter culture was then spread onto plates prepared from LB agar (3.2%) (Cat. L3027, Sigma-Aldrich) containing ampicillin (100 µg/ml) (Cat. A5354, Sigma-Aldrich) and incubated overnight at 37°C. The next day a single colony was picked from each plate using a pipette tip and added to 5 ml of broth with ampicillin (100 µg/ml), followed by incubation for 4h in a shaking incubator (250 rpm) at 37°C to form a starter culture. Subsequently, 1 ml of the starter culture was transferred to 250 ml of LB broth (2.0%) (Cat. L3522, Sigma-Aldrich) with ampicillin (100 µg/ml) in sterile a 1-liter flask and incubated overnight at 37°C in a shaking incubator (250 rpm).

Plasmid DNA was purified using a PureLink™ HiPure Maxiprep kit (Cat. K210006, ThermoFisher Scientific). Briefly, cell cultures were pelleted by centrifugation (4600 g, 15 min at RT) to remove supernatant. Pellets were resuspended in suspension buffer, followed by an equal volume of lysis buffer, mixed by inversion and incubated 3 min at RT. Lysates were transferred to a filter column to remove cell debris. The lysate was clarified with binding buffer (10 ml) and passed through a purification column by centrifugation (500g, 2 min) followed by two rounds of wash buffer (800 µl, 17,000g, 1 min). DNA was eluted with 100 µl elution buffer; concentration and purity were confirmed on a spectrophotometer (1-3 ng/µl; 260/230 nm ratio >2.0) (Nanodrop). DNA inserts were confirmed by long-read sequencing (Nanopore-30, Source Bioscience).

## Heterologous expression of mouse Raet1e in HEK293T cells

HEK293T cells were seeded on coverslips previously coated with PDL (10 µg/ml) in 500 μl DMEM (Cat. 41965-039, Gibco) including 10% heat-inactivated FBS (Cat. 16140071, Life Technologies) in a 24-well plate (5x10^4^ cells per well) and incubated overnight at 37°C, 5% CO_2_. After approximately 48h (2 days) in culture, HEK cells were transfected with plasmid DNA using polyethylenimine (PEI). PEI (2.8 μg/well) was added dropwise to DNA (1 μg/well) in 150 mM NaCl and vortexed before adding the PEI/DNA mixture dropwise to the cell culture media. Cells were incubated 4 h at 37°C, 5% CO2, washed with 300 μl of fresh DMEM (including 10% HI-FBS) and maintained for a further two days. On the fourth day, cells were treated with recombinant human NKG2D-Fc chimeric receptor or Fc-control (1 μg/ml) in DMEM including 1% BSA, for 1h at 37°C, 5% CO2. Cells were then washed 3x PBS and fixed with PFA (4% diluted in 0.01M PBS) for 20-30 min before immunolabelling for human IgG (see NKG2D immunolabelling above).

## Confocal imaging

Immuno-labelled cultures were imaged on a laser scanning confocal microscope (LSM700, Zeiss) fitted with 3 laser lines (405, 488, and 546 nm). Individual neurons were first identified in the 405 nm laser channels corresponding to B-tubulin immunolabelling. Z-stack images (3-4 x 1 µm) of 405 nm, 488 nm and 546 nm channels were acquired at 1024 x 1024 resolution (12 bit) with a x20 air objective. 488 nm (Fc receptor) acquisition settings (i.e. gain, laser power, pin hole size) remained constant throughout all experiments.

For high-throughput quantification of NKG2D immunolabelling in sensory neuron cultures over time, coverslips were imaged on a spinning disc confocal microscope (iXplore SpinSR10, Olympus) with 4 Laser lines (405, 488, 561 and 640nm) fitted to an inverted microscope (IX83, Olympus) using stage navigator with Z-Drift Compensation for automated, systematic sampling. For mouse DRG neurons 100 regions of interest (ROI) were systematically sampled in a 10x10 grid aligned with the centre of the coverslip. For hiPSCd sensory neurons, 10 ROI were systematically sampled along the axis of laser ablation (see **Supp. Figure 6**). Z-stack images (3 x 2 µm) of 405 nm, 488 nm and 546 nm channels were acquired at 1156 x 1156 resolution (16 bit) with a x40 air objective. Identical laser and acquisition settings were maintained throughout.

An imaging pattern was designed for evenly taking ten images of both proximal and distal axons automatically by Spinning Disc confocal microscopy, which was applied to all the microfluidics. The distance between each ROI of proximal axions and microfluidics channel as well as between each ROI of proximal axons and the horizontally parallel ROI of distal axons was same. Each image containing 5 Z-slices with 2 µm step-size per slice was taken with a 40X objective. Zero-Drift Compensation (ZDC) was applied for maintaining the distance between objective and each ROI.

Super-resolution images of recombinant NKG2D receptor protein bound to *Mrgprd*+ DRG neurites were acquired on a Yokogawa CSU-W1SoRa Spinning Disc confocal microscope fitted with 4 Laser lines (405, 488, 561 and 640nm). 3D rendering and animation was performed using Zen 2012 software (Zeiss).

## Confocal image analysis

Analysis of NKG2D labelling of DRG neurons was performed using manual and automated methods. For manual counting, the number of NKG2D-binding neurons in the different subpopulations, individual β-tubIII+ neurons in a given field of view were manually assigned as either NKG2D-positive or negative while blinded to the expression of TdTomato. Images where neuronal densities were too high for identification of individual neurons (as assessed by the observer) were excluded from analysis.

For automated quantification of NKG2D labelling of murine DRG neurites, a bespoke analysis pipeline was executed using Fiji [17], in combination with a method for soma detection [18] and run via a script in MatLab with minor changes from the original publication to allow for batch processing of multiple images. The full script, including instructions for its use, can be downloaded from GitHub [19]. In brief, raw image files were imported to Fiji (ImageJ 2) by running Macro A, splitting into channels representing BtubIII (*blue*), NKG2D/Fc (*green*) and TdTomato (*red*) and generating pictures with a thresholded mask of the BtubIII channel representing total neuronal area. The newly generated image with a single channel BtubIII was imported to Matlab, where a soma mask array was generated via the Dimensionality Ratio method using the SomaExtraction package [18]. The mask array was imported back to Fiji to generate soma mask files by running Macro B. The soma mask was used to segment DRG neuron soma from neurites. Area selections (Regions of Interest, ROI) for βtubIII+ and TdTomato+ neurites were enlarged by 0.5 and transferred to the pre-thresholded NKG2D/Fc (*green*) channel for receptor quantification using the Particle Analysis function (size=0.1-400 µm^2^; circularity=0-1.00). Receptor density (particles per µm^2^) was calculated according to the original neurite area ROI for both βtubIII+ and TdTomato+ channels. A cut off of 200 µm^2^ was set for the minimum neurite area per ROI for receptor particle quantification. In experiments involving Thy1-YFP mice, the NKG2D and tdTomato fluorescence channels were reversed for quantitative analysis.

For automated quantification of NKG2D/Fc binding to human iPSC-derived sensory neurons, maximum intensity projection (MIP) images of BtubIII+ channel and the respective segmented images were generated by running Macro A. Debris with large size was identified by running SomaExtraction package on Metlab and Macro B on Fiji. The generated soma (debris) mask images were used for removing the noise from unspecific binding of NKG2D/Fc to debris generated by laser ablation. Area selections (Regions of Interest, ROI) for βtubIII+ were enlarged by 0.5 and transferred to the pre-thresholded NKG2D/Fc (*green*) channel for receptor quantification using the Particle Analysis function (size=0.1-400 µm^2^; circularity=0-1.00) by running Macro C. Receptor density (particles per µm2) was calculated according to the original neurite area ROI for BtubIII+ channel. The analysis pipeline can be found on GitHub [20].

The fragmentation of human iPSC-sensory neuron was quantified using semi-automated method [13]. The full Macro script can be found on GitHub [21]. Briefly, the area of axons (Area 1) and the area of BtubIII-positive particles (Area 2) in a same image were automatically measured by Macro script in Fiji. The fragmentation (%) = Area 1/Area 2 × 100.

## Human natural killer (NK) cell isolation and stimulation

Human NK cells were isolated from the peripheral blood of three healthy volunteers, as well as leukocyte cones from three volunteer blood donors supplied by UK National Health Service Blood and Transplant (NHSBT) Non-Clinical Issue (NCI) service.

Peripheral blood was collected by venepuncture in sodium-heparin tubes. Whole blood was then diluted 1:1 in serum-free RPMI 1640 media (Cat. 21875034, Life Technologies) and suspended on 15 ml Lympholyte Human Cell Separation Media (Cat. CL5020, CedarLane) in 50 ml SepMate tubes (Cat. 85450, Stem Cell Technologies) and centrifuged 22 min at 800g at room temperature (RT) with no brake to separate peripheral blood mononuclear cells (PBMC). Plasma containing platelets was removed and the leukocyte layer transferred to fresh RPMI containing 10% foetal bovine serum (heat-inactivated) (Cat. F9665-500ML, Sigma-Merck) and washed by pellet centrifugation at 500g, 10 min, RT. Live PBMC were counted by trypan blue exclusion and 10^8^ cells per sample were transferred to magnetic cell sorting (MACS) buffer (0.01M phosphate buffered saline (PBS) plus 2mM EDTA and 2% FBS). NK cells were enriched by MACS negative selection using a human NK Cell Isolation Kit (Cat. 130-092-657, Miltenyi Biotech) with LD columns (Cat. 130-042-901, Miltenyi-Biotech), according to the manufacturer’s instructions. Unlabelled NK cells were eluted into MACS buffer and counted with trypan blue exclusion for downstream applications. 4.65 – 6.75 x 10^6^ live NK cells were isolated per 10^8^ PBMC per donor. Approximately 10^6^ purified NK cells were sampled from each donor for purity check by flow cytometry analysis. Remaining NK cells were suspended at 10^6^ cells/ml in cryopreservation media (50% RPMI, 40% FBS, 10% DMSO) and frozen using a controlled-rate alcohol-free cell freezing container (CoolCell, Corning) before transfer to vapour phase nitrogen for long-term cryostorage.

Cone blood was diluted 1:1 in PBS containing 1% BSA and suspended on 15ml of Lympholyte Human Cell Separation Media (Cat. CL5020, CedarLane) in 50 ml Falcon tube and centrifuged 22 min at 800g (slow acceleration [3] and deceleration [0]) at room temperature (RT) to separate peripheral blood mononuclear cells (PBMCs). PBMCs located in the interphase (‘buffy coat’) between Lympholyte and plasma layers were carefully collected into a new 50ml Falcon tube and then washed by MACS buffer by pellet centrifugation 10 min at 400g at room temperature. 100x10^6^ PBMCs were resuspended in 2ml of MACS buffer. NK cells were enriched by negative selection using EasySep^TM^ Human NK Cell Isolation Kit (Stem Cell, Catalog #17955), according to the manufacturer's instructions. The isolated NK cells were suspended in cryopreservation media (50% RPMI, 40% FBS, 10% DMSO) and frozen using a controlled-rate alcohol-free cell freezing container (CoolCell, Corning) in -80°C before being transferred to vapour phase nitrogen for long-term cryostorage.

For NK cell stimulation, vials of cryopreserved NK cells were rapidly thawed in a water bath at 37°C and washed in RPMI including 10% FBS supplemented with DNase I (125 U/ml) followed by centrifugation at 400g, 10 min, RT. Cells were counted and seeded at 2x10^6^ cells per ml in RPMI plus 10% FBS in 96 well U-bottom plates (Nunclon Delta, Cat. 10344311, FisherScientific) supplemented with recombinant human IL-2 (10^3^ U/ml; 100ng/ml equivalent) (Cat. 200-02, Peprotech) and cultured for 2 days at 37°C, 5% CO_2_.

## Flow cytometry

Whole PBMC, NK-depleted fraction and purified human NK cells (5x10^5^ cells per 100 µl) were suspended in FACS buffer (PBS +2% FBS) and blocked with normal human serum (NHS, 10%) for 15 min on ice. Cells were treated with fluorescently conjugated antibodies (see **Supp. Table 5**) and incubated 40 min at 4°C protected from light. Cells were washed 2x FACS buffer with centrifugation 500g, 5 min. The fluorescent DNA intercalator 7-aminoactinomycin D (7-AAD) (1:100) was added to all samples (except single stain controls) prior to cytometry. Samples underwent flow cytometry on an LSRII Special Order Research Product (SORP) digital cell analyser equipped with a Violet (405nm, 100mW), Blue (adjustable 488nm, 80mW), Green (532nm, 150mW) and Red (642nm, 40mW) lasers. Prior to data acquisition the cytometer was calibrated using CS&T Beads. PMT gain and compensation settings were established using single and unstained control PBMC samples. Due to limited spill-over between fluorophores, fluorescence-minus-one controls were not employed. Approximately 100,000 events were run per sample. Data were exported as .fcs files and analysed in FlowJo. Lymphocytes were gated based on characteristic forward and side scatter. Marker gates were set based on single cell controls. The gating strategy was as follows: Lymphocytes -> 7AAD^neg^ -> singlets -> CD19^neg^CD3^neg^ -> CD56^dim^CD16^+^ (cytotoxic NK) versus CD56^bright^CD16^neg^ (regulatory NK).

NKG2D receptor expression levels, as well as production of perforin and granzyme B by NK cells were analysed by full spectrum flow cytometry (Aurora 5 laser Spectral Flow Cytometer, Cytek) equipped with UV (355nm, 20mW), violet (405nm, 100mW), blue (488nm, 50mW), 50mW yellow-green & red (638nm, 80mW) lasers. Quality control (QC) was performed by running SpectroFlo QC beads before acquiring samples, automatically optimizing laser and the gains (voltages) for every detector. Then FSC (Forward Scatter) and SSC (Side Scatter) were adjusted to ensure that cells were not off scale. Reference controls without staining or single staining only were acquired for spectral unmixing and FMO (fluorescence minus one) controls were prepared for defining the boundaries of positive and negative cell populations. Raw data were unmixed by SpectroFlo and the unmixed data were analysed by FlowJo (version: 10.10.0). 1x10^6^ cells were resuspended in 100 µl FACS buffer (PBS containing 2% FBS) were incubated with LIVE/DEAD Fixable Blue (Cat. L34961, Biolegend) 20 min on ice protected from light. Cells were washed twice with FACS buffer. Cells were incubated with 10% normal mouse serum on ice for 15 min and then incubated with anti-CD45 (Cat. 563792, BD Biosciences, RRID: AB_2869519), anti-CD3 (Cat. 612941, BD Biosciences, RRID: AB_2916883), anti-CD14 (Cat. 301831, Biolegend, RRID: AB_2563629), anti-CD11c (Cat. 612967, BD Biosciences, RRID: AB_2870241), anti-CD56 (Cat. 318333, Biolegend, RRID: AB_2561912), anti-CD16 (Cat. 741449, BD Biosciences, RRID: AB_2870923), anti-NKG2D (Cat. 320818, Biolegend, RRID: AB_2562792) for 45 min on ice protected from light (see **Supp. Table 5**). Cells were washed twice with FACS buffer and then fixed with Fixation and Permeabilization Solution (Cat. 554714, BD Biosciences) on ice for 20 min. Cells were washed twice with Perm/Wash Buffer (BD Biosciences) and incubated with anti-perforin (Cat. 48-9994-42, Life Technologies, RRID: AB_2574145) and anti-granzyme B (Cat. 58-8896-42, Life Technologies, RRID: AB_2724390). Cells were washed twice with Perm/Wash Buffer (BD Biosciences) and resuspended in 100 µl FACS buffer for full spectrum flow cytometer analysis.

## NKG2D blocking assay

For antibody blockade of human NKG2D function *in vitro*, NK cells were incubated with 50 μg/ml of LEAF purified anti-human CD314 (NKG2D) (clone 1D11) (Cat. 320813, Biolegend; RRID: AB_2561488) or LEAF purified human IgG1 isotype control (Clone QA16A12) (Cat. 403501, Biolegend; RRID: AB_2927629) for 30 min at room temperature (2.5x10^6^ NK cells per ml) before addition to target cells (hiPSCd sensory neurons) in neurite compartment of microfluidic devices. Cells were seeded at different densities (50x10^3^, 100x10^3^, 200x10^3^) for 4 h.

NK cells were added to the neurite compartment of microfluidic devices approximately 4 weeks after hiPSCd-sensory neuron seeding and co-cultured for 4 h. Then cells were washed three times by HBSS, followed by fixation by 4% PFA for 30 min. Fixed cells were washed three times by HBSS and blocked and permeabilized by PBS containing 5% normal serum and 0.1% Triton-X100 for 1 h at room temperature. Then cells were incubated with primary antibody diluted in PBS containing 0.5% normal serum and 0.01% Triton-X100 overnight at 4°C. Cells were washed three times by PBS and incubated with DAPI and secondary antibody diluted in PBS containing 0.5% normal serum and 0.01% Triton-X100 for 1 h at room temperature. Then cells were washed three times by PBS and the reservoirs of microfluidics were filled up with PBS and covered with coverslip.

## Quantitative PCR

### RNA extraction from mouse DRG cultures

After various time points in culture (day 1, 2, and 3), coverslips were gently washed with PBS before lysis with a phenol-containing lysis buffer (Tripure, Roche). Lysates were transferred to 1.5 mL samples tubes, snap-frozen on dry ice, and stored at -80°C until RNA extraction using filter column purification (High Pure RNA Tissue Kit, Cat. 12033674001, Roche). Samples from the same batch of cultures were processed simultaneously by thawing at room temperature (RT) for 5 min. 100 µL of chloroform was added to each sample, samples were shaken for 15 s, then incubated at room temperature for 15 min. Samples were centrifuged at 12,000G for 15 min at 4°C. 200 µL of the upper aqueous phase was added to 200 µL of 70% ethanol in a filter column, which was centrifuged at 13,000G for 30 s. DNase solution was added, and the column incubated at RT for 15 min. RNA was purified by a series of wash steps according to the manufacturer’s instructions followed by elution buffer and centrifugation at 8,000G for 1 min. RNA eluates were checked for concentration and purity on a NanoDrop Microvolume Spectrophotometer and stored at -80°C until reverse transcription.

### RNA extraction from mouse DRG tissues

Total RNA was extracted from bilateral L3-L5 DRG after spared nerve injury (one mouse per sample). Tissues were disrupted in RTL Plus lysis buffer (QIAGEN) including 1% β-mercaptoethanol with a mini glass mortar and pestle on ice and further homogenized using a Minilys bead homogenizer (Precellys, Bertin, France); DRG cultures were washed once in warm HBSS prior to lysis by pipetting; frozen cell pellets were vortexed in lysis buffer. RNA was purified from lysed samples, including genomic DNA elimination, by on-column extraction (RNeasy Plus, QIAGEN) according to the manufacturer’s instructions. RNA was eluted in RNase-free water and analyzed for purity (260/280 nm ratios of approximately 2.0 were considered acceptable) and nucleotide content on a spectrophotometer.

### Reverse transcription

Reverse transcription for quantitative PCR was performed using Moloney Murine Leukemia Virus (M-MLV) reverse transcriptase kit (Cat. 28025-013) with oligo(dT) primers (Cat. 58862) (Invitrogen, Thermofisher). Briefly, equal amounts of RNA (50 ng for cultured DRG, 150-250 ng for DRG tissues) was added to 1 µL Oligo(dT)12-18 (500 µg/mL), and 1 µL 10mM dNTP in PCR-grade water in a nuclease-free reaction tube (total volume 12 µL). The mixture was heated to 65°C for 5 min then quick-chilled on ice. To each tube was added: 4 µL 5X First-Strand Buffer, 2 µL 100mM DTT, and 1 µL RNase OUT (40 units/ml) (Cat. 100000840) and mixed gently. Samples were incubated at 37°C for 2 min before adding 1 µL (200 units) of M-MLV RT to each tube, mixing by gentle pipetting and incubation at 37°C for 50 minutes, before heating at 70°C for 15 min. Complimentary DNA (cDNA) products were stored at -20°C until use in PCR experiments. Universal Mouse Reference RNA (Cat. QS0640, Life Technologies) and DRG-extracted RNA with omission of reverse transcriptase were used as positive and negative controls, respectively.

### Quantitative polymerase chain reaction (qPCR)

Quantitative gene expression in DRG cell cultures was performed on cDNA (0.33 ul per tube per sample from original reverse transcription) using a SYBR Green PCR Master Mix (Roche) and pairs of target-specific primers (500 nM each) in a 10 µl sample volume on white skirted 384-well PCR plate (Cat. E1042-9909, StarLab) on a LightCycler 480 (Roche). The PCR conditions were: 95°C (5 min) and cycled 45 times at 95°C (30 s) to 60°C (2 min) followed by melt curve for PCR product confirmation: 65°C (1 min), ramped to 97°C (0.11 °C/s).

Quantitative gene expression was analysis performed on cDNA from DRG tissues using a Power SYBR Green PCR Master Mix (Applied Biosystems) and pairs of target-specific primers (500 nM) in MicroAmp optical tubes (20µl reaction volume) on a 7500 Real-Time PCR system (Applied Biosystems). The PCR conditions were 50°C (2 min), 95°C (10 min), and cycled 40 times at 95°C (15 s) to 60°C (1 min). Data were analysed using the built-in 7500 software (v2.0.4, Life Technologies).

All samples were run in triplicate. Expression was determined relative to a reference gene (*Gapdh*) using the comparative Ct method [22]: 2^-ΔΔCt^ = 2^-(Ct of Gapdh – Ct of target gene^). ΔΔCt values were normalised to day 0 DRG to present as fold change in transcripts at each time point for each culture, or to contralateral or naïve DRG for nerve injury experiments. Primers were designed using PrimerBLAST [23] or obtained from previous literature (See **Supp. Table 6**). Primers had GC content between 40% and 60%. Where possible, primers were designed to overlap the exon-exon boundary, and 3’ ends were G or C Annealing temperatures of primer pairs were within 5°C of each other. OligoCalc [24] was used to select primers with minimal potential for hairpin formation, 3’ complementarity and self-annealing, and were validated by a single peak in the dissociation curve or single band PCR product via gel electrophoresis.

### Gel electrophoresis

qPCR reaction products (10 µL) were run on 1.5% agarose gel stained with GelRed (Cat. SCT123, Merck Millipore) at 100V for 30 min with 100 bp ladder (Cat. N3231S, New England Biolabs). The gels were imaged using a UV illuminator (NuGenius, Syngene).

## Single cell DRG collection, reverse transcription and nested PCR

Adult male C57BL/6 mouse DRG neurons were cultured as above overnight in Neurobasal media supplemented with NGF (50 ng/ml) on coverslips previously coated with poly-D-lysine (10 µg/ml) and laminin (10 µg/ml). Coverslips were transferred to a patch-clamp recording chamber and perfused with a 2Ca^2+^/Na^+^ buffer containing (in mM): 140 NaCl, 10 HEPES, 2 CaCl_2_, 1 MgCl_2_, 10 glucose, 5 KCl, pH 7.2 in DEPC-treated water. Micropipettes for cell collection with a tip diameter of approximately 10 µm were prepared from borosilicate glass previously washed with DEPC-treated water and autoclaved to eradicate contaminating RNase activity. Single DRG neurons were collected into the pipette containing a reverse transcription buffer by applying gentle negative pressure under visual control and were immediately ejected into a collection buffer containing dNTP, oligo(dT) and random hexamers. Negative controls were provided by collection of the perfusion solution only. Samples were heated to 65°C for 5 min and cooled on ice before the addition of reaction buffer containing reverse-transcriptase (Superscript III, Invitrogen, Life Technologies). cDNA was synthesised on a thermo-cycler (Biorad T100) (25°C, 5 min; 50°C, 100 min; 85°C, 5 min) and finally 37°C for 20 min in the presence of RNase H. cDNA was stored at -20°C. PCR was performed with nested primers using Platinum *Taq* DNA polymerase (Invitrogen) according to the manufacturer’s instructions. The first round of PCR was performed with 1-1.5 µl single cell cDNA and ‘outer’ primer pairs (95°C, 5 min followed by melting at 94°C for 40 s, annealing at 52-60°C for 40 s and elongation at 72°C for 40 s, cycled 35 times). The second round of PCR was performed with 2 µl of the first round PCR product, ‘inner’ primer pairs and cycled 20 times. Second round PCR products were visualised by gel electrophoresis stained with SafePinky (GenDepot). Nested primer pairs were synthesised by a commercial supplier (Bioneer, Korea) (see **Supp. Table 7**).

## In situ hybridization (RNAscope)

In situ hybridization was performed according to the manufacturer’s instructions for the RNAscope^TM^ Multiplex Fluorescent Reagent Kit v2 Kit (Advanced Cell Diagnostics, Cat. 323100). In brief, the fixed-frozen mouse DRG sections mounted on Superfrost slides were allowed to reach room temperature and then washed twice to remove OCT compound followed by ethanol (100%) treatment for 2 min and hydrogen peroxide for 10 min. After antigen retrieval by submerging in the boiling retrieval buffer for 2min and proteases III treatment at 40°C for 10 min, the sections were then incubated with probe Raet1 (ACD, Cat. 448121, targeting all Raet1 isoforms) at 40°C for 2 h. The sections were then incubated with three rounds of amplification reagents followed by signal development with TSA Vivid Fluorophore 520 (ACD, Cat. 323271). To combine with immunofluorescence staining, the sections were blocked with 10% normal goat or donkey serum, 0.3% Triton-X 100 in 0.01M PBS at RT for 1h followed by anti-NeuN (Merck Millipore, Abn91, RRID: AB_11205760; 1:400) and IB4 (Biotin-conjugated, Sigma, L2140, RRID: AB_2313663; 1:200) or anti-NeuN (1:400) and anti-mCherry (OriGene, AB0040, RRID: AB_2333093; 1:400) overnight at 4°C. The sections were then incubated with goat anti-chicken 405 (Invitrogen, A48260, RRID: AB_2890271; 1:1000) and Alexa Fluor 647-conjugated streptavidin (Invitrogen, S21374, RRID: AB_2336066; 1:200) or biotinylated goat anti-chicken antibody (Vector, BA-9010, RRID: AB_2336114; 1:200), pacific blue-conjugated streptavidin (Invitrogen, S11222, RRID: Not registered; 1:200) and donkey anti-goat 546 (Invitrogen, A11056,  RRID: AB_142628; 1:1000) at RT for 1h. For DRG tissues taken 6 days after partial crush nerve injury [10], 3 sections per L4 DRG per side (ipsi/contra) from each mouse were imaged (n=3 mice). Totally n= 1914 neurons from contralateral DRG and n=1914 neurons from ipsilateral neurons were analyzed. For DRG tissues taken 3 weeks after spared nerve injury [9], 4 sections per DRG (L3-5) per side (ipsi/contra) from a single mouse were imaged (n=1 mouse). In total, n=1196 neurons from contralateral DRG and n=2135 neurons from ipsilateral DRG were analyzed.

## RNA sequencing dataset analysis

Human DRG (hDRG) sequencing data were downloaded as transcript-per-million (TPM) counts for Wangzhou et al (2020) [25] and quantile normalized TPM (qnTPM) from Ray et al., 2023. [26]. In line with published their published reports, only hDRG with clear neuronal enrichment were used from Ray et al., 2023. Ensembl IDs were mapped to gene symbols using biomaRt in R.

Visium data were accessed as a Seurat Object from Tavares-Ferreira et al. (2022) [27]. Single soma hDRG count data were downloaded from GEO (GSE249746) [28]. iPSC (AD2, AD3 and 840 lines) and iPSC-derived sensory neuron data were downloaded from GEO (GSE144208) [29].

Non-neuronal cells from a cross-species harmonized DRG atlas were downloaded from the ‘[painseq](https://painseq.shinyapps.io/harmonized_painseq_v1/)’ dataset [30] as a Seurat object. Human barcodes were extracted, and the object was subset for the “Immune” cluster. The extracted RNA counts were used to create a fresh object prior to downstream processing in Seurat. Data were normalized (`NormalizeData `), and variable features extracted ` FindVariableFeatures (seu.immune, selection.method = "vst", nfeatures = 2000)`. After scaling, and elbow plot was used to determine dimensions (here, 10). Clustering was performed as `FindClusters (seu.immune, resolution = 1)`. The original published object was mapped to mouse gene names, thus this naming was maintained for plotting.

Human sural nerve data was accessed from the ‘osmzhlab’ dataset [31]. Mouse DRG neuron data was accessed from the ‘ernforslab’ dataset [32]. Plots were downloaded as png files from the corresponding Shiny application.

Data were processed in line with their publication. Briefly, Seurat objects were created for individual donors (n=6) in R, and normalized (`NormalizeData (seurat_obj)`). Variable features were calculated on the top 4500 features (`FindVariableFeatures(seurat_obj, nfeatures = 4500)`). Integration anchors were calculated using FindIntegrationAnchors(object.list = seurat_objects, scale=TRUE, normalization.method="LogNormalize", reduction="cca", l2.norm = TRUE, dims=1:30, k.anchor=5, k.filter=200, k.score=30, max.features=200, nn.method="annoy", n.trees=50, eps=0). Objects were then integrated using IntegrateData(ss.data, normalization.method="LogNormalize", features=NULL, features.to.integrate=NULL, dims=1:30, k.weight=80, weight.reduction=NULL, sd.weight=1, sample.tree=NULL, preserve.order=FALSE, eps=0). Data were then scaled (`ScaleData `) prior to dimensionality reduction (RunPCA(ss.data, npcs=50), RunUMAP(ss.data, reduction="pca", dims=1:25)). Clusters were then calculated using ` FindNeighbors(ss.data, reduction="pca", dims=1:25)` and `FindClusters(ss.data, resolution=3.4)` in line with published methods from Yu et al. (2024). Metadata were extracted from the project-associated github (taimeimiaole/NN_hDRG-neuron-sequencing/Source_code_2/human_meta_final_cluster.Rdata), with `cl.conserv_final` set as Idents for plotting.

All plots were generated in R, using ggplot2 and/or Seurat, with code available at https://github.com/aliibarry/NK.

## Study design and statistics

For *in vivo* experiments, biological unit of interest is the animal (i.e. number of mice). For murine cell culture experiments the biological unit of interest is either the animal (i.e. number of cultures for RNA analysis) or the cell (i.e. number of DRG neurons for immunohistochemical analysis), unless otherwise defined; where individual neurons were not defined (i.e. during automated image analysis), the biological unit was a region of interest. Mice of both sexes were used unless otherwise indicated. For human cell culture experiments, the biological unit of interest is the individual donor. Experimental units are defined in figure legends. Microfluidic devices without NK cells were excluded from data analysis because of the low density of axons. Where multiple observations were made of RNA levels or immunohistochemical signals *in vitro*, the various treatments (including controls) were applied to replicate experimental units (i.e. cell culture well) derived from each biological unit.

Each replicate cell culture contained the full suite of experimental and control samples. Assignment of cell culture wells to different time points or treatment with different reagents (i.e. recombinant proteins or cells), or assignment of animals to treatment groups, was not randomised. Counting of neurons was performed on images offline after manual image acquisition. DRG neuron culture from a single male mouse for resulted in low levels of extracted RNA (<5 µg/ml) at days 1, 2 and 3 *in vitro* Fig. **2B**), therefore data from this animal was excluded from analysis.

For *in vivo* experiments, sample sizes (i.e. number of animals) were based on previous publication [10]. In situ hybridization (RNAscope) experiments were performed based on availability of mouse tissues cryopreserved as a legacy of previous studies in the lab. *In vitro* experiments were exploratory and therefore sample size was not calculated *a prior*. All the graphs, calculations and statistical analyses were performed using GraphPad Prism software 10.0. Data points represent mean values of replicate measurements with standard error of the mean, unless otherwise stated. Pair-wise comparisons of normally distributed data were analysed using a two-tailed Student’s t test. Pair-wise comparisons of non-normally distributed data were analysed using a two-tailed Mann-Whitney U test. Where the experimental unit is a single image (i.e. when calculating receptor particle density) data were compared as cumulative distributions using the Kolmogorov-Smirnov test and presented as violin plots showing median and quartile ranges. Alpha=0.05.

All data points were included in the data analysis except for the excluded data points described above.

# Supplementary Figures


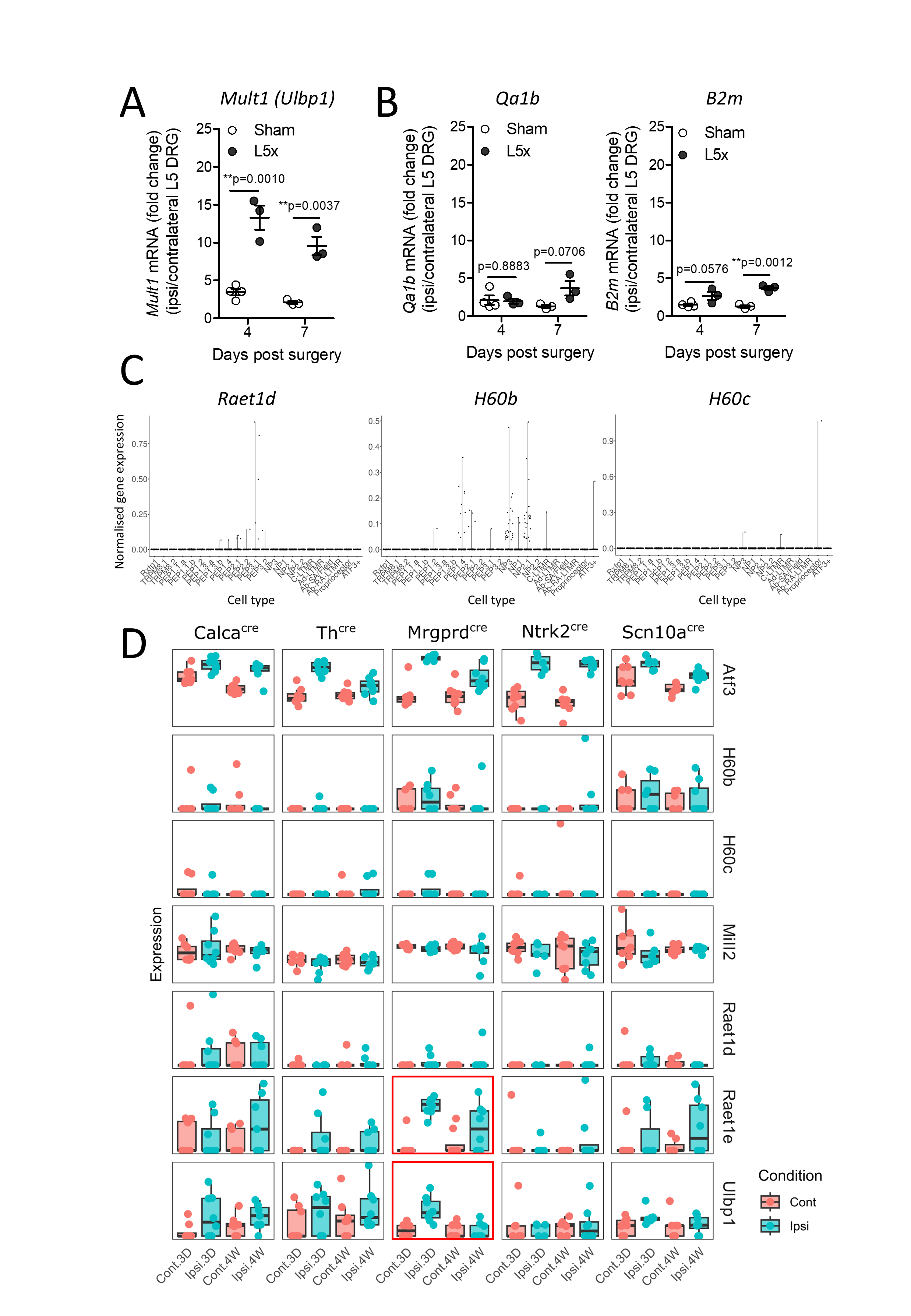


Supplementary Figure 1. Regulation of NK cell stimulatory and inhibitory ligands in DRG by L5x injury. **A**) qPCR showing the effect of L5x or sham injury on *Mult1 (Ulbp1)* mRNA transcript levels in L5 DRG 4 days and 7 days after surgery*.* Students unpaired t test: 4d sham *versus* L5x, t=6.804, p=0.0010; 7d sham *versus* L5x, t=6.099, p=0.0037. **B**) *Qa1b.* Students unpaired t test: 4d sham *versus* L5x, t=0.1478, p=0.8883; 7d sham *versus* L5x, t=2.448, p=0.0706. And *B2m.* Students unpaired t test: 4d sham *versus* L5x, t=2.427, p=0.0596; 7d sham *versus* L5x, t=8.184, p=0.0012. n=3m mice per time point per group. **C**) Violin plots showing normalised expression of *Raet1d*, *H60b* and *H60c* among mouse DRG neuronal subtypes. Data from integrated atlas curated by Krauter et al., 2025 [33]. **D**) Box plots of NKG2D ligand expression in each of five sensory neuron lineages after nerve injury. Gene expression levels in individual animals are presented as VST (variance stabilizing transformation) transformed counts in ipsilateral and contralateral lumbar DRG neurons 3 days and 4 weeks after spared nerve injury (SNI). Regulation of *Raet1e* and *Ulbp1* in *Mrgprd*+ neuron population highlighted in red. Injury marker gene *Atf3* shown as positive control. Data from Barry et al., 2023 [9].


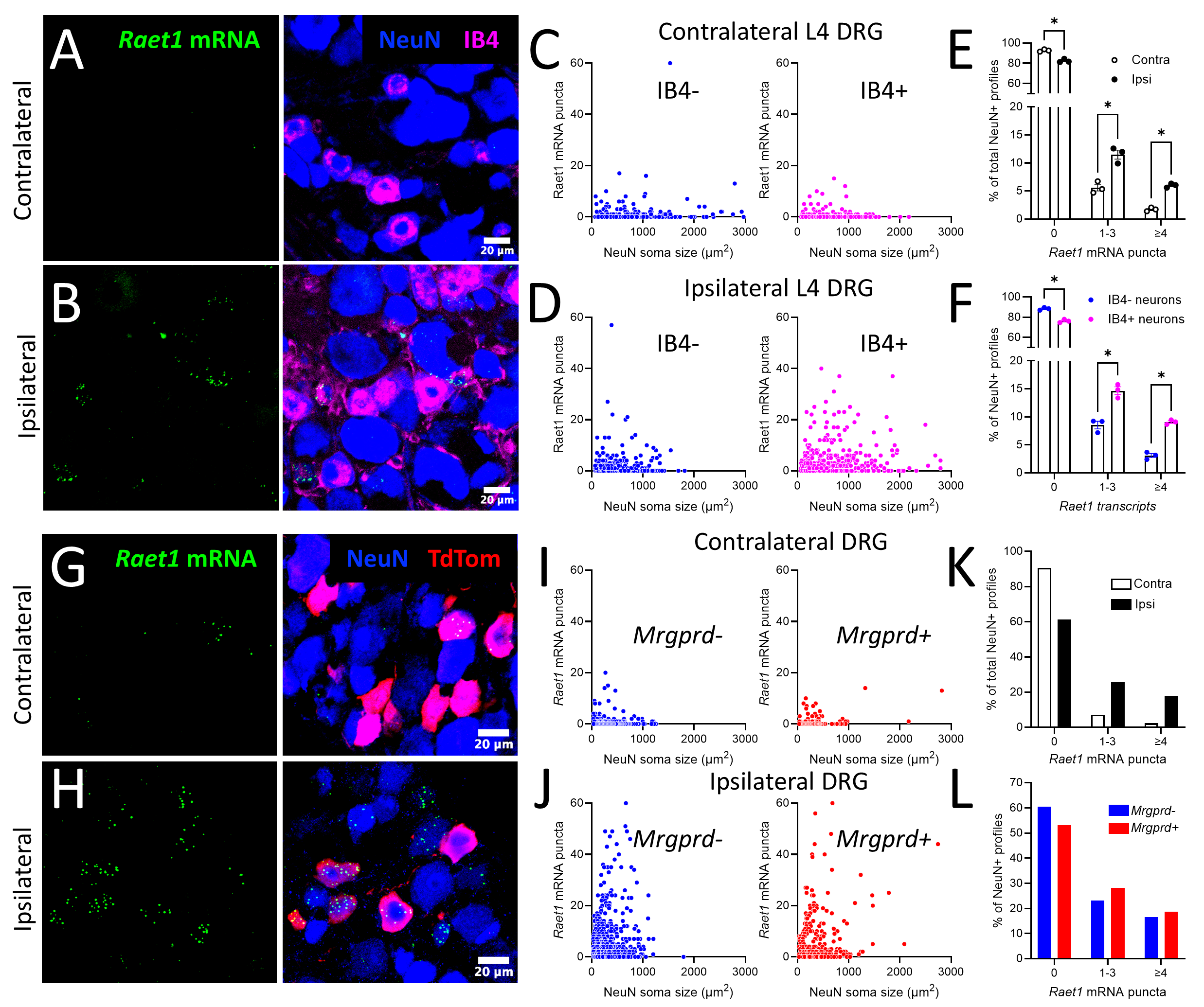


Supplementary Figure 2. In situ hybridisation (RNAscope) for *Raet1* mRNA in lumbar DRG of mice after nerve injury. **A**) *Raet1* mRNA (green) with NeuN (blue) and IB4 (magenta) colocalization in contralateral and **B**) ipsilateral L4 DRG 6 days after partial crush nerve injury in wild type mice. Scale bars, 20 µm. **C**) Dot plots showing number of *Raet1* mRNA puncta within individual NeuN+ neuron profiles in contralateral L4 DRG according to cell size (µm^2^). *Left*) IB4-negative neuron profiles (1160 neurons total); *Right*) IB4 positive neuron profiles (754 neurons total). **D**) Dot plots showing number of *Raet1* mRNA puncta within individual NeuN+ neuron profiles in ipsilateral L4 DRG according to cell size (µm^2^). *Left*) IB4-negative neuron profiles (1468 neurons total); *Right*) IB4 positive neuron profiles (1303 neurons total). **E**) Percentage of ipsilateral and contralateral DRG neurons with *Raet1* mRNA puncta. (n=3 mice, unpaired t test with Welch correction, Sidak-Bonferroni method *p<0.05). **F**) Percentage of IB4 positive and negative neurons with *Raet1* mRNA puncta in ipsilateral DRG (n=3 mice, unpaired t test with Welch correction, Sidak-Bonferroni method *p<0.05). **G**) *Raet1* mRNA (green) with NeuN (blue) and TdTomato (red) colocalization in contralateral and **H**) ipsilateral L3-5 DRG from *Mrgprd*-cre;TdTom mice 3 weeks after spared nerve injury (SNI). Scale bars, 20 µm. **I**) Dot plots showing number of *Raet1* mRNA puncta within individual NeuN+ neuron profiles in contralateral L4 DRG according to cell soma size (µm^2^). *Left*) Mrgprd-negative neuron profiles (1160 neurons total); Right) Mrgprd-positive neuron profiles (754 neurons total). **J**) Dot plots showing number of *Raet1* mRNA puncta within individual NeuN+ neuron profiles in ipsilateral L4 DRG according to cell soma size (µm^2^). *Left*) Mrgprd-negative neuron profiles (1468 neurons total); *Right*) Mrgprd-positive neuron profiles (595 neurons total). **K**) Percentage of ipsilateral and contralateral DRG neurons with *Raet1* mRNA puncta (n=1 mouse). **L**) Percentage of Mrgprd positive and negative neurons with *Raet1* mRNA puncta in ipsilateral DRG (n=1 mouse).

**
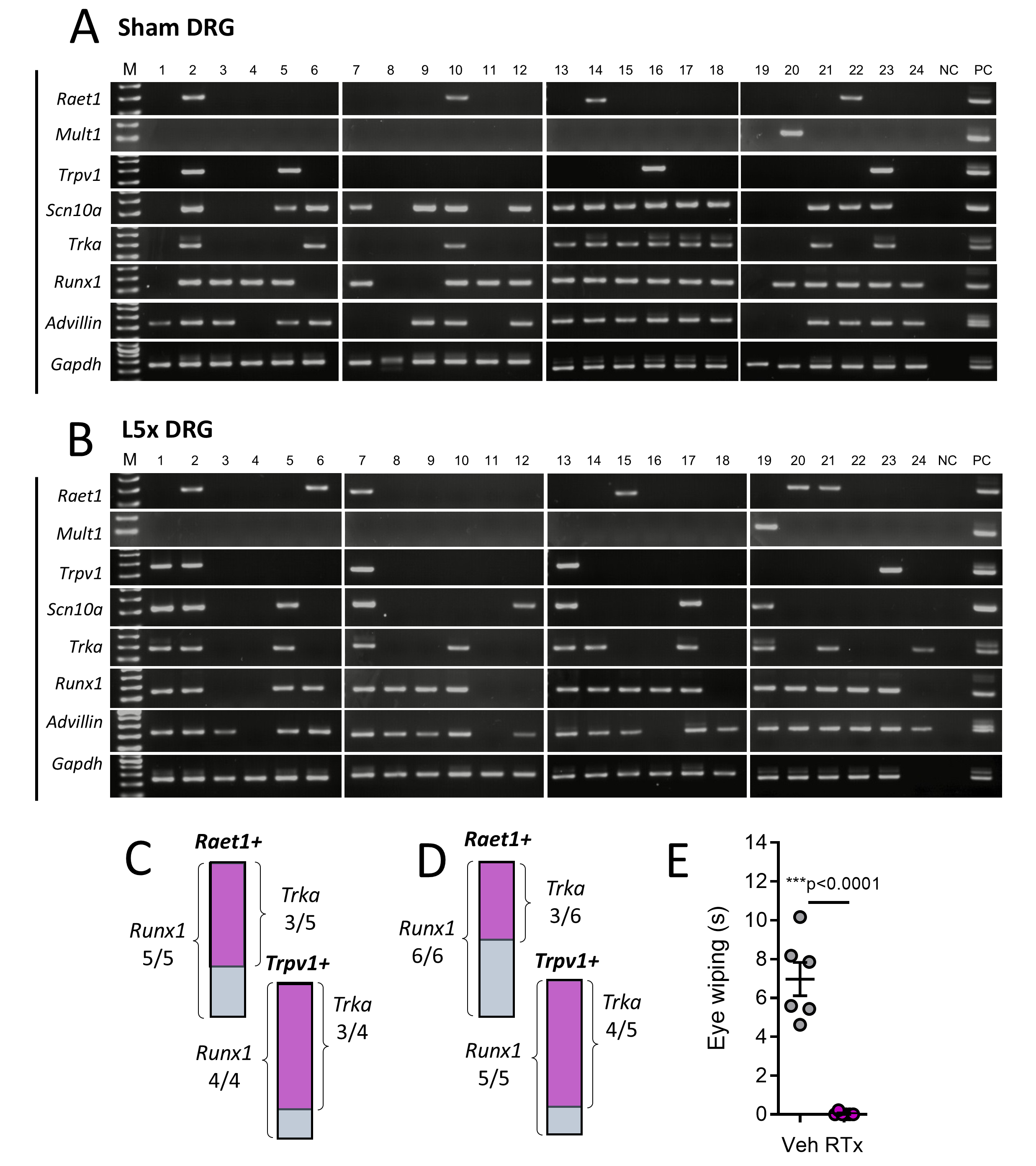
**

Supplementary Figure 3. Single cell reverse-transcription PCR of mRNA transcripts in acutely isolated adult mouse DRG. Full results of single cell nested PCR for multiple mRNA transcripts detected in adult DRG neurons isolated from either mice 7 days after **A**) sham, or **B**) L5 spinal nerve transection (L5x) surgery and maintained for <24h *in vitro.* PCR bands for 24 DRG neurons collected and lysed are shown with DNA ladder marker, M. NC, negative control (bath solution). PC, positive control (whole DRG tissue). **C**) The proportion of *Raet1*+ and *Trpv1+* sham DRG neurons also expressing the transcription factors *Runx1* and *TrkA*. **D**) The proportion of *Raet1*+ and *Trpv1+* L5x DRG neurons also expressing the transcription factors *Runx1* and *TrkA*. **E**) RTx-treated mice did not respond to corneal application of capsaicin (0.01%, 10 µl). Student’s unpaired t test: t=8.055, p<0.0001, n=6 mice per group.

**
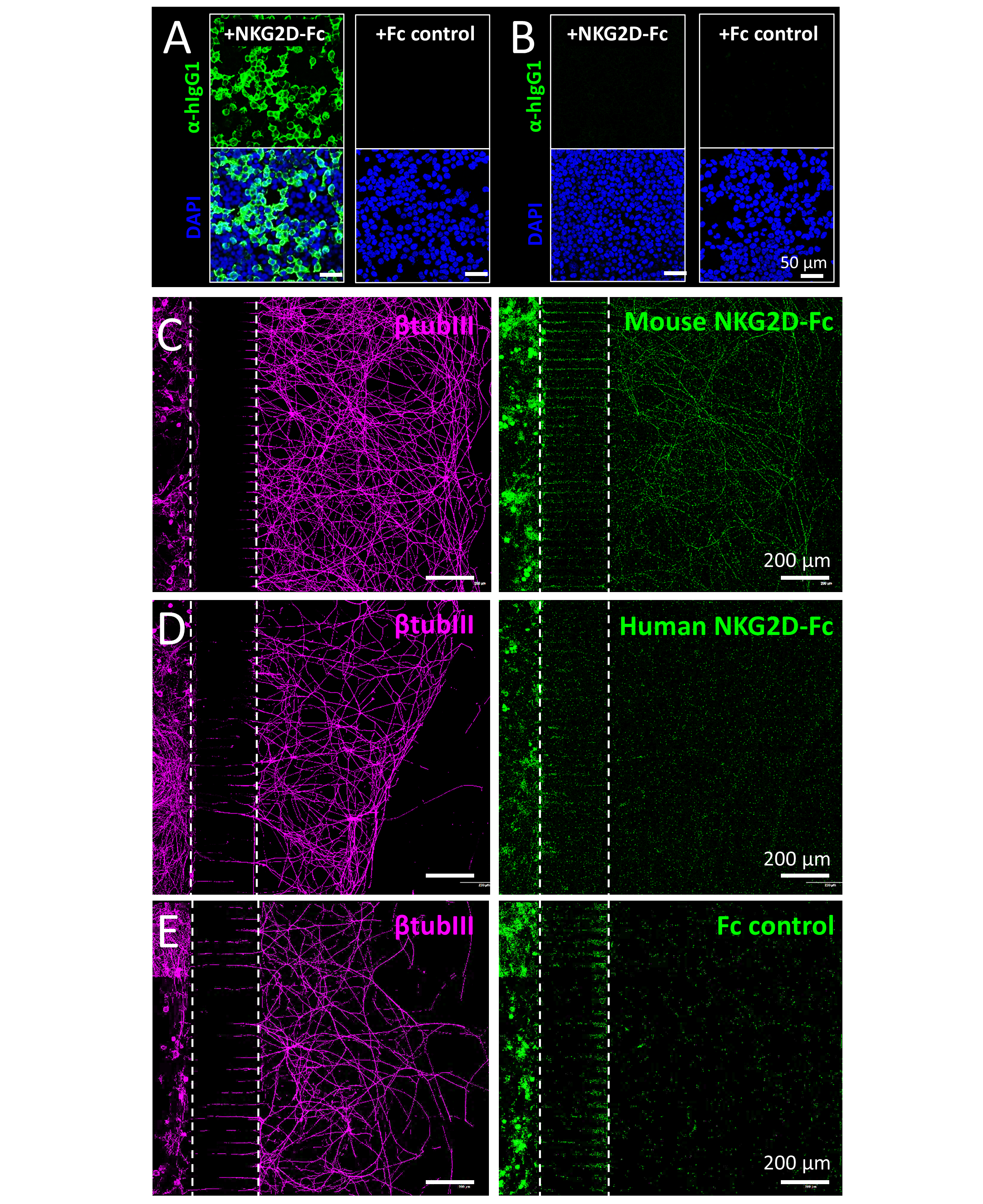
**

Supplementary Figure 4. Development of a live cell-based assay for mouse NKG2D ligands. *Above*) HEK293T cells transfected with **A**) mouse *Raet1e* or **B**) untransfected controls treated with mouse NKG2D-Fc or Fc only control protein (2 µg/ml). Human IgG1-Fc detection (*green*) and DAPI (*blue*). Scale bars, 50 µm. *Below*) Mouse DRG neurons grown 5 days in microfluidic devices were treated live with **A**) mouse NKG2D-Fc, **B**) human NKG2D-Fc or **C**) Fc fragment recombinant proteins (5 µg/ml) for 1h at 37°C before fixation and immunolabelling. Btubulin III (magenta), anti-human IgG1 (green). Note labelling of mouse DRG neurites with mouse NKG2D-Fc protein only. Scale bars, 200 µm.

**
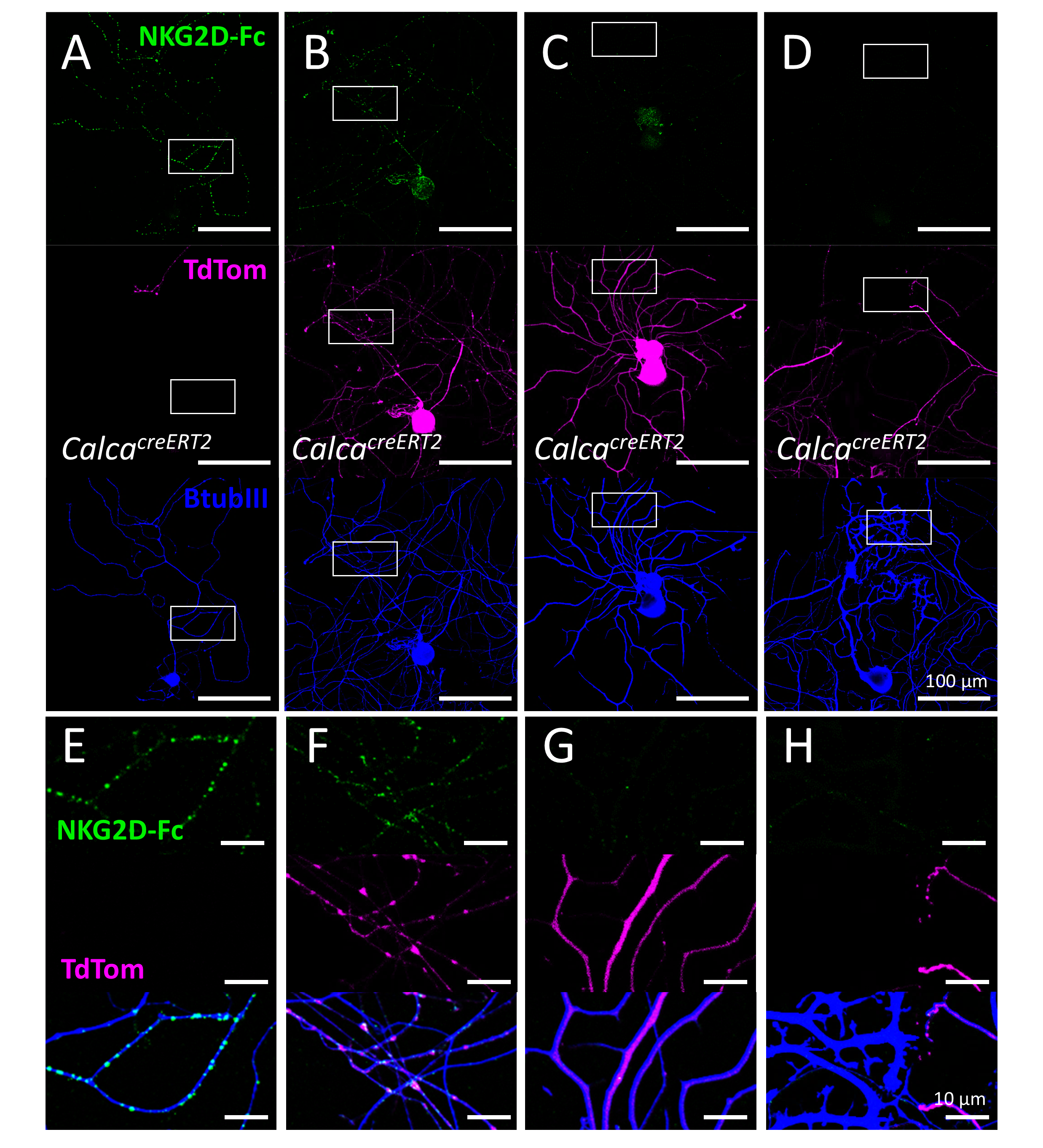
**

Supplementary Figure 5. Examples of NKG2D labelling in sensory neurons from Calca-TdTom reporter line. **A**) TdTom^neg^ neuron (*magenta*) with high density of NKG2D-Fc receptor particle binding (*green*). **B**) TdTom^+^ neuron (*magenta*) with high density of NKG2D-Fc receptor particle binding (*green*). **C**) TdTom^+^ neuron (*magenta*) lacking NKG2D-Fc receptor particle binding. **D**) TdTom^neg^ neuron (*magenta*) lacking NKG2D-Fc receptor particle binding. BtubIII counterstain, *blue*. **E-H)** High magnification of insets above in A-D. Scale bars A-D, 100 µm; scale bars E-H, 10 µm.


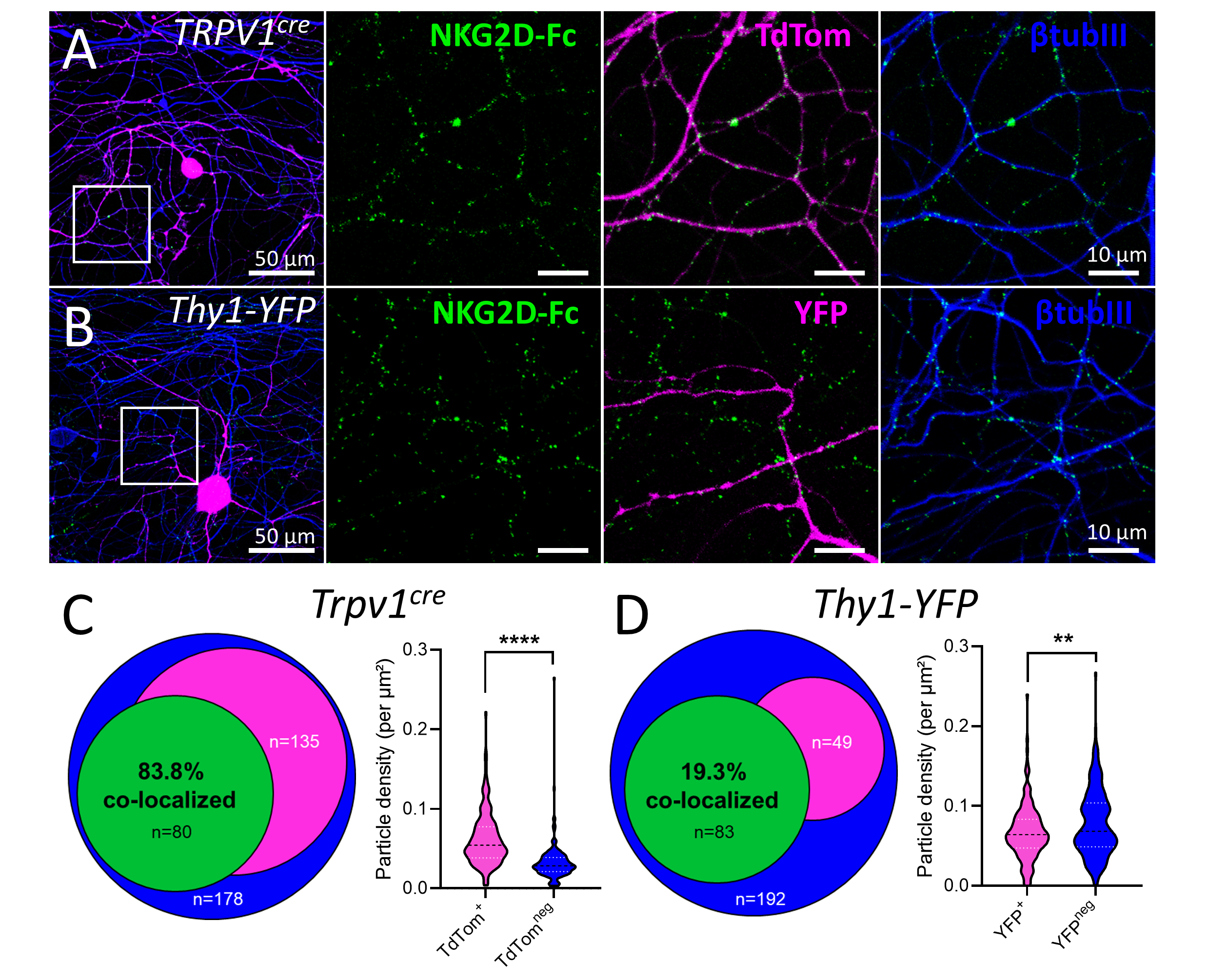


Supplementary Figure 6. NKG2D labelling in sensory neurons from Thy1-YFP and TRPV1- TdTom reporter line. DRG neuron were cultured for 3 days prior to live cell labelling with NKG2D-Fc. Low magnification confocal image of DRG neurons from A) Trpv1-cre;TdTomato and B) Thy1-YFP mice 3 days in vitro (left), with high magnification images of inset (right). Scale bars as indicated. C, D) Quantification of NKG2D receptor binding to the neurites of DRG neurons from the different genetic sensory neuron lineages. Venn diagrams illustrate the proportions of DRG neurons lineages displaying NKG2D binding assessed by manual counting. ‘n’ represents numbers of neurons manually counted. Violin plots illustrate NKG2D receptor particle density per µm2 of neurite area per image (images pooled from multiple coverslips from multiple cultures (i.e. mice). C) Trpv1-lineage sensory neurons. Manual counting: n=178 neurons from n=3 mice; 2 male, 1 female. Automated image analysis: TdTom+, n=169 images; TdTomneg, n=168 images; ****P<0.0001 Kolmogorov-Smirnov test (D=0.5731). D) Thy1-lineage sensory neurons. Manual counting: n=192 neurons from n=3 mice; 2 male, 1 female. Automated image analysis: YFP+, n=168 images; YFPneg, n=201 images; Kolmogorov-Smirnov test, **P=0.0012 (D= 0.2015). Median and quartiles represented within violin plots as black and white dotted lines, respectively. Note: YFP fluorescence in Thy1 line has been re-coloured to magenta for continuity of presentation.


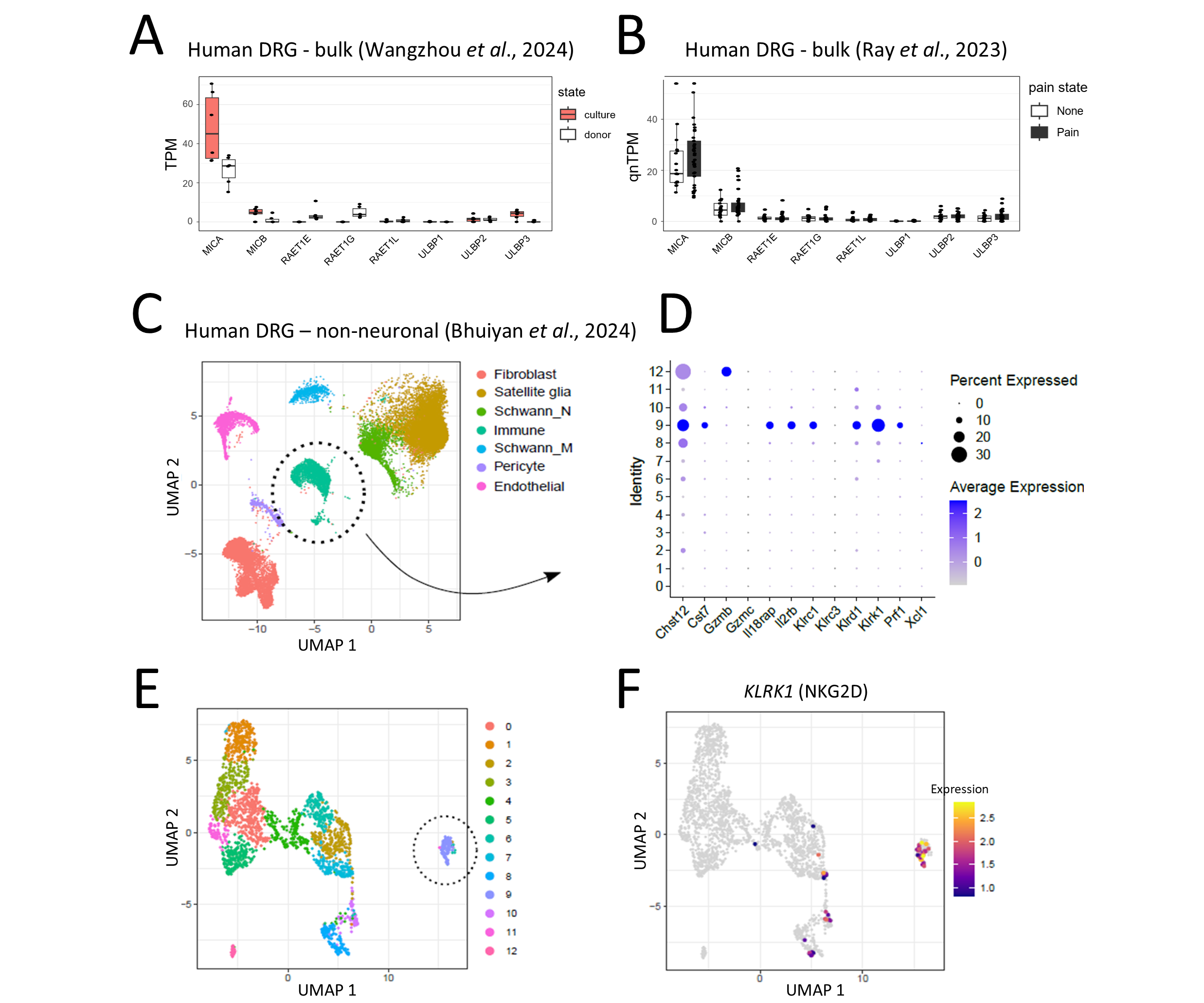


Supplementary Figure 7. Analysis of human DRG bulk and single-cell RNA sequencing datasets. **A**) Relative NKG2D ligand gene transcript levels in primary human DRG (bulk RNA sequencing): native tissue from frozen ganglia versus 4 days *in vitro* culture. Data from Wangzhou et al., 2020 [25]. **B**) Relative NKG2D ligand gene transcript levels in whole primary human DRG from thoracic vertebrectomy (bulk RNA sequencing): pain versus non-pain donor. Data from Ray et al., 2023 [26]. **C**) Identification of 7 core cell populations including peripheral immune cells in human DRG tissues by analysing the published harmonized somatosensory non-neuronal cell atlases. Data from Bhuiyan et al., 2024 [34]. **D**) Clustering of 13 immune subsets by analysing the RNA counts extracted from 'Immune' cell population showing enrichment of NK cell-related gene expression. **E**) UMAP of immune subsets in human DRG. **F**) Enrichment of *KLRK1* in cluster 9 expressing NK/T hall markers.


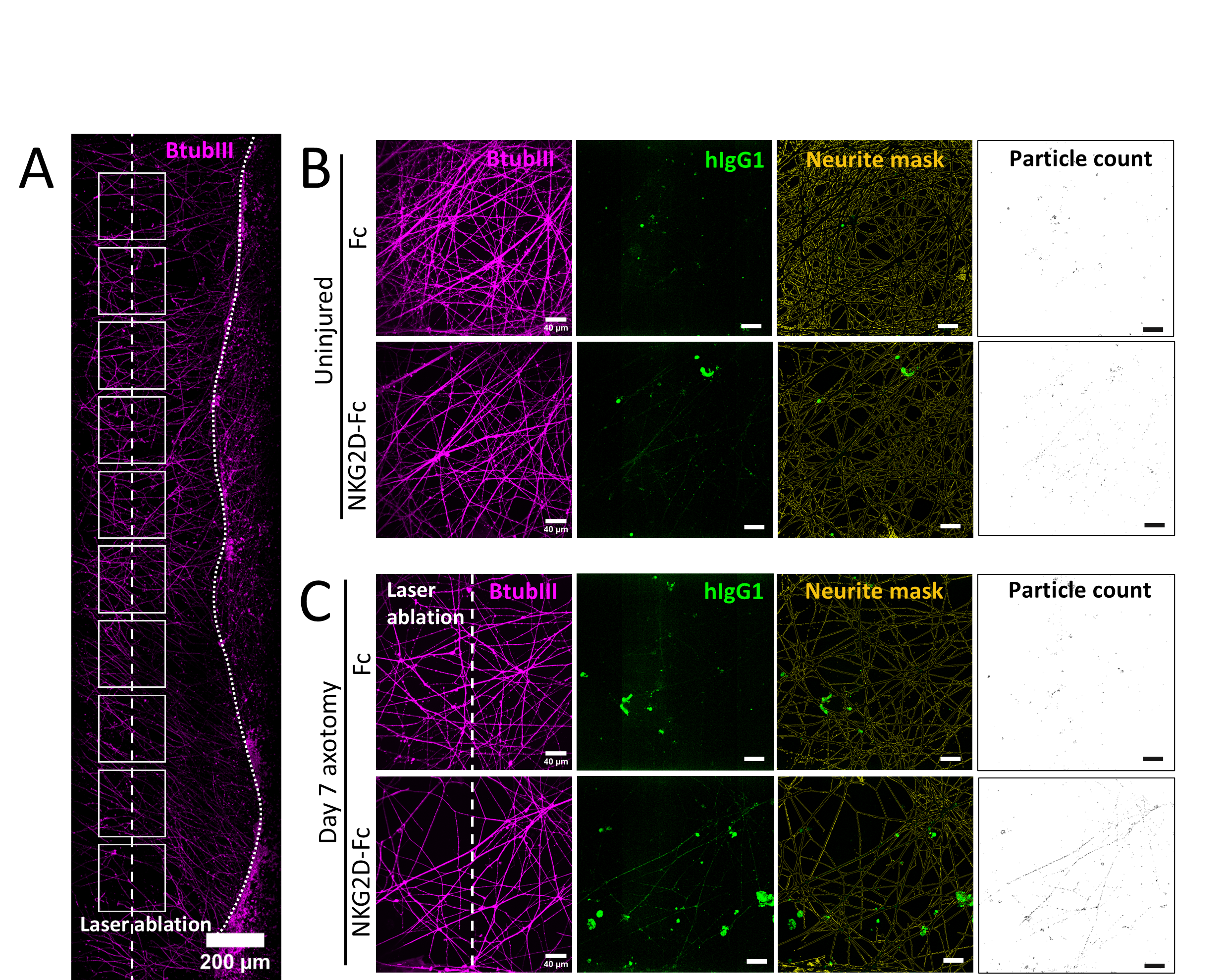


Supplementary Figure 8. Systematic analysis of receptor binding to hiPSCd sensory neuron axons after laser ablation. **A**) Low magnification image and representation of a pre-programmed image sampling strategy for the systematic imaging of injured axons. Images were acquired automatically within wells of 24-well plates by spinning disc confocal microscopy using the Navigation function in conjunction with Z-drift compensation (ZDC). Vertical dashes indicate original line of laser ablation. Dotted line indicates retraction of cells after axotomy. White squares indicate 10x ROIs along line of axotomy. **B**, **C**) Representative images of uninjured and injured (day 7) axons labelled with βtubIII (*magenta*) and Fc control or NKG2D-Fc (*green*), which were analysed by a Macro script in Fiji. ‘Selection’ of BtubIII+ axons was restored to the corresponding Fc/NKG2D-Fc binding images respectively, generating ‘Neurite mask’ (*yellow*), within which ‘Particle count’ (black dots) was analysed.

**
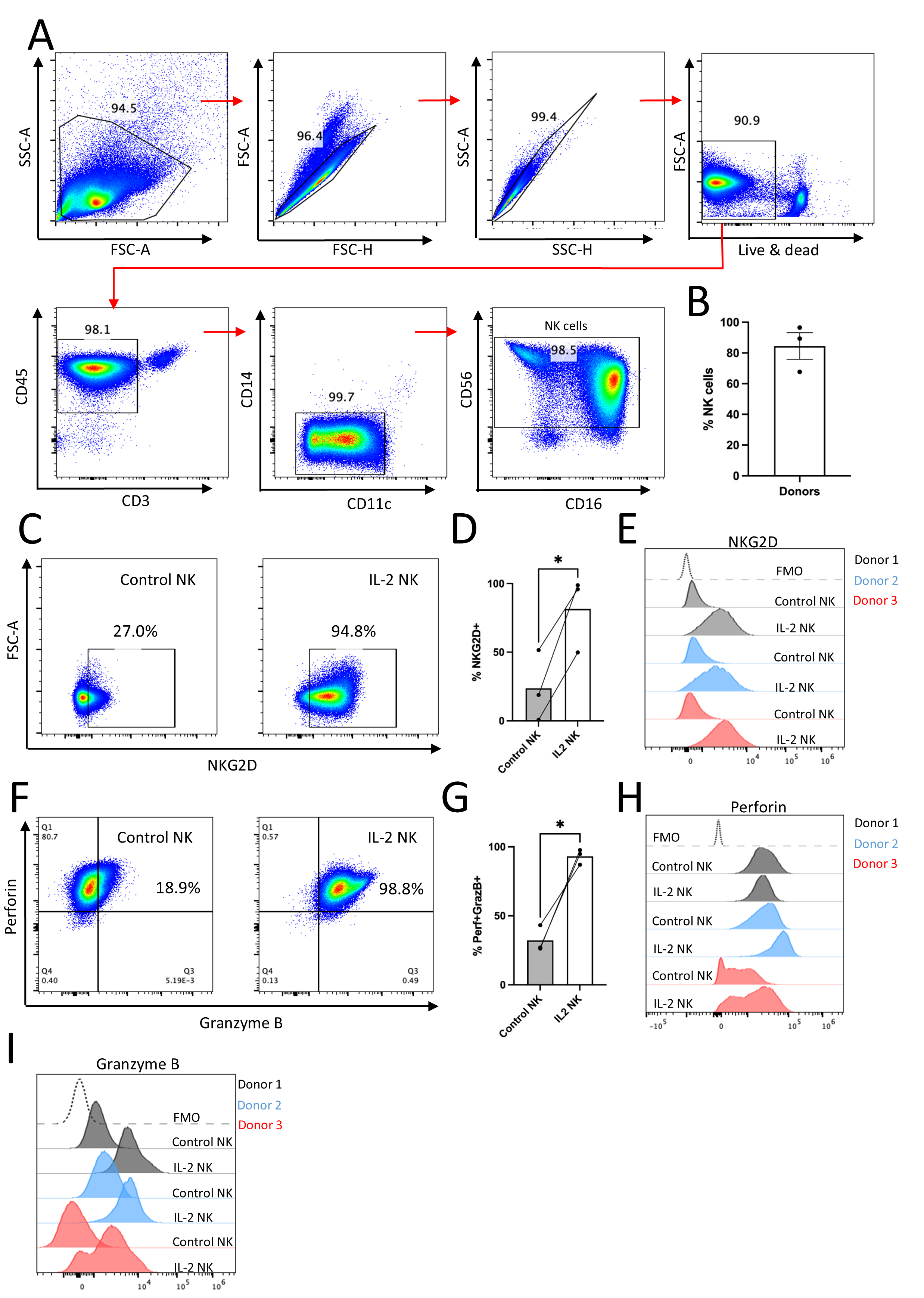
**

Supplementary Figure 9. Purity and stimulation of human NK cells. **A)** Flow cytometry for analysing the purity of human NK cells enriched from PBMCs. There were almost no T cells (CD45^+^CD3^+^), monocytes (CD14^+^CD11c^+^) and dendritic cells (CD14^-^CD11c^+^). The majority (98.5%) of CD45^+^CD3^-^CD14^-^CD11c^-^ are NK cells including CD56^hi^CD16^-^ and CD56^dim^CD16^+^ subsets. **B)** Purity of NK cells isolated from three healthy donors. **C-F**) Human NK cells from each donor were stimulated by IL-2 (1000 U/ml) for 2 days before being culture with hiPSCd sensory neurons. **C**) Flow cytometry plots of NKG2D gated on NK cells (CD45^+^CD3^-^CD14^-^CD11c^-^CD56^hi^CD16^-^ and CD45^+^CD3^-^CD14^-^CD11c^-^CD16^dim^CD16^+^). **D**) Quantification of NKG2D^+^ in purified NK cells after IL-2 stimulation (IL-2 NK) compared to unstimulated (freshly thawed) cells (control NK) *p=0.0351 (t=5.194), paired Student’s t test, n=3 donors per group. **E**) Histograms of the fluorescence intensity of NKG2D expressed by unstimulated (Control NK) or IL-2 stimulated NK (IL2 NK) cells from three healthy donors. **F**) Flow cytometry plots perforin and granzyme B gated on NK cells (CD45^+^CD3^-^CD14^-^CD11c^-^CD56^hi^CD16^-^ and CD45^+^CD3^-^CD14^-^CD11c^-^CD16^dim^CD16^+^). **G**) Quantification of perforin^+^granzyme B^+^ double-positive NK cells. *p=0.0195 (t=7.054), paired Student’s t test, n=3 donors per group. **H**) Histograms of perforin fluorescence intensity on unstimulated (Control NK) or IL-2 stimulated NK (IL2 NK) cells from three healthy donors. **I**) Histograms of granzyme B fluorescence intensity on unstimulated (Control NK) or IL-2 stimulated NK (IL2 NK) cells from three healthy donors. FMO, ‘fluorescence minus one’ control.

**
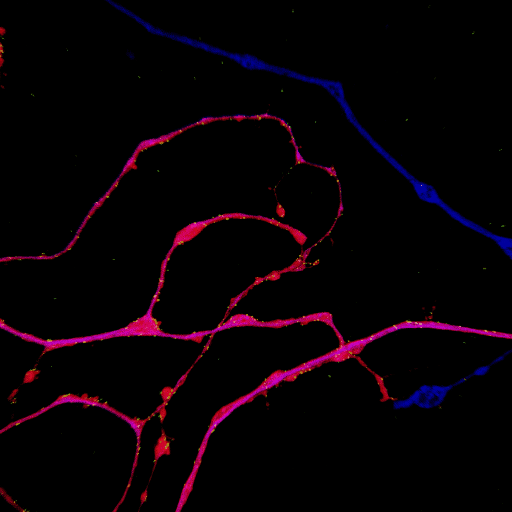
**

Supplementary Video 1. Super-resolution image of NKG2D receptors bound to DRG neurites in vitro. 3D rendering of *Mrgprd*+TdTomato-expressing neurites (*magenta*) labelled with rec recombinant NKG2D receptor labelling (*green*) imaged at super-resolution on a spinning disc confocal microscope. DRG neurons treated with soluble NKG2D receptors at 3 days *in vitro* and co-labelled with B-tubulin III (*blue*).

# Supplementary Tables

## Supplementary Table 1. Genetically altered mouse lines.

| **Mouse line** | **Abbreviation** | **Supplier** | **Cat. No.** | **RRID** | **Gifted by** |
| --- | --- | --- | --- | --- | --- |
| *B6.129(Cg)-Scn10a^tm2(cre)Jwo^ [1]* | *Nav1.8^cre^* | The Jackson Laboratories | 036564 | IMSR_JAX:036564 | Prof. John Wood |
| *Calca^tm1.1(cre/ERT2)Ptch^ [2]* | *Calca^creERT2^* | N/A | N/A | N/A | Prof. Pao-Tien Chuang |
| *Mrgprd*^tm1.1(cre/ERT2)Wql^/J [3] | *Mrgprd^creERT2^* | The Jackson Laboratories | 031286 | IMSR_JAX:031286 | Prof. David Ginty |
| *Th*^tm1.1(cre/ERT2)Ddg^/J [4] | *Th^creERT2^* | The Jackson Laboratories | 025614 | IMSR_JAX:025614 | Prof. David Ginty |
| *B6;Cg-Tg(Thy1)-^(YFP)16Jrs^/J [5]* | Thy-1 YFP-16 | The Jackson Laboratories | 003709 | IMSR_JAX:003709 | Prof. Pilhan Kim |
| *B6.129-Trpv1^tm1(cre)Bbm^/J [6]* | *Trpv1^cre^* | The Jackson Laboratories | 017769 | IMSR_JAX:017769 | N/A |
| *B6;129-Gt(ROSA)26Sor^tm1(DTA)Mrc^/J [7]* | *R26R^DTA^* | The Jackson Laboratories | 010527 | IMSR_JAX:010527 | N/A |
| *B6.Cg-Gt(ROSA)26Sor^tm14(CAG-tdTomato)Hze^/J [8]* | Ai14 | The Jackson Laboratories | 007914 | IMSR_JAX:007914 | N/A |

## Supplementary Table 2. iPSC donor lines

| **iPSC line**​ | **Sex**​ | **Age**​ | **ID**​ | **RRID**​ | **Allosomes**​ | **Aberrations​** | **Copy**  **number**​ | **Size**  **(kbp)**​ | **Gene**  **count**​ |
| --- | --- | --- | --- | --- | --- | --- | --- | --- | --- |
| SFC-**AD2**-01 | Male​ | 51​ | STBCi321-A​ | CVCL_ZX54​ | XY​ | None​ | 2.0​ | 0​ | 0​ |
| SFC-**840**-03-03​ | Female​ | 67​ | STBCi026-A​ | CVCL_RB85​ | XX​ | None​ | 2.0​ | 0​ | 0​ |

## Supplementary Table 3. Antibodies for immunofluorescence staining

| **Antibody/protein** | **Clone** | **Cat. No.** | **Supplier** | **RRID** | **Dilution factor** |
| --- | --- | --- | --- | --- | --- |
| Rabbit anti-BtubIII | Polyclonal | T2200 | Sigma Aldrich | AB_262133 | 1:2000 |
| Chicken anti-NeuN | Polyclonal | Abn91 | Merck Millipore | AB_11205760 | 1:400 |
| IB4-biotin conjugate | NA | L2140 | Sigma | AB_2313663 | 1:200 |
| Goat anti-mCherry | Polyclonal | AB0040 | OriGene | AB_2333093 | 1:400 |
| Goat anti-rabbit IgG Alexa Fluor 546 | Polyclonal | A-11035 | Invitrogen | AB_2534093 | 1:1000 |
| Goat anti-rabbit IgG Pacific blue | Polyclonal | P-10994 | Invitrogen | AB_2539814 | 1:1000 |
| Donkey anti-rabbit IgG Alexa Fluor 647 | Polyclonal | A-31573 | Invitrogen | AB_2536183 | 1:1000 |
| Goat anti-human IgG Alexa Fluor 488 | Polyclonal | A-11013 | Invitrogen | AB_2534080 | 1:750 |
| Goat anti-chicken Alexa Fluor 405 | Polyclonal | A48260 | Invitrogen | AB_2890271 | 1:1000 |
| Streptavidin Alexa Fluor 647 | NA | S21374 | Invitrogen | AB_2336066 | 1:200 |
| Biotinylated goat anti-chicken | Polyclonal | BA-9010 | Vector | AB_2336114 | 1:200 |
| Streptavidin Pacific Blue | NA | S11222 | Invitrogen | NA | 1:200 |
| Donkey anti-goat 546 | Polyclonal | A11056 | Invitrogen | AB_142628 | 1:1000 |

## Supplementary Table 4. Plasmid details

| **Plasmid** | **Gene**  **(RefSeq)** | **Promoter** | **Tag / Reporter** | **Bacterial selection marker** | **Vector Name** | **Catalog#** | **Supplier** |
| --- | --- | --- | --- | --- | --- | --- | --- |
| GFP-Control | (Empty control vector) | CMV | C-eGFP | Ampicillin | pReceiver-M03 | GEN-EX-NEG-M03 | Genepoeia Inc |
| RAE-1-ε-untag | *Raet1*ε  (NM_198193.2) | CMV | - | Ampicillin | pReceiver-M02 | EX-Mm18607-M02 | Genepoeia Inc |
| RAE-1-ε-eGFP | *Raet1*ε  (NM_198193.2) | CMV | C-eGFP | Ampicillin | pReceiver-M03 | EX-Mm18607-M03 | Genepoeia Inc |

## Supplementary Table 5. Antibodies for flow cytometry

| **Antigen** | **Clone** | **Fluorophore** | **Supplier** | **Catalogue number** | **RRID** | **Dilution factor** |
| --- | --- | --- | --- | --- | --- | --- |
| CD56 | CMSSB | APC | eBioscience | 17-0567-41 | AB_10596498 | 1:200 |
| CD16 | B73.1 | PE | eBioscience | 12-0167-41 | AB_1603210 | 1:400 |
| CD3 | OKT3 | FITC | eBioscience | 11-0037-41 | AB_2016601 | 1:1000 |
| CD19 | HIB1 | APC-eFluor 780 | eBioscience | 47-0199-41 | AB_1582231 | 1:1000 |
| LIVE/DEAD Fixable | N/A | UV excitation | Invitrogen | L34961 | N/A | 1:500 |
| CD45 | HI30 | BUV395 | BD Biosciences | 563792 | AB_2869519 | 1:100 |
| CD3 | UCHT1 | BUV496 | BD Bioscience | 612941 | AB_2916883 | 1:100 |
| CD14 | M5E2 | BV570 | Biolegend Inc | 301832 | AB_2563629 | 1:50 |
| CD11c | B-ly6 | BUV661 | BD Biosciences | 612967 | AB_2870241 | 1:50 |
| CD56 | HCD56 | BV605 | Biolegend Inc | 318334 | AB_2561912 | 1:50 |
| CD16 | B73.1 | BUV563 | BD Biosciences | 741449 | AB_2870923 | 1:100 |
| NKG2D | 1D11 | PerCP/Cyanine5.5 | Biolegend Inc | 320818 | AB_2562792 | 1:50 |
| Perforin | dG9 | eFluor 450 | Invitrogen | 48-9994-42 | AB_2574145 | 1:50 |
| Granzyme B | N4TL33 | Alexa Fluor 532 | Invitrogen | 58-8896-42 | AB_2724390 | 1:100 |

## **Supplementary Table 6.** Primer sequences for qPCR

| Target gene | GenBank reference | Forward sequence | Reverse sequence | Product size (bp) |
| --- | --- | --- | --- | --- |
| *Actin* | [NM_007393.5](https://www.ncbi.nlm.nih.gov/nuccore/NM_007393.5) | GGCTGTATTCCCCTCCATCG | CCAGTTGGTAACAATGCCATGT | 154 |
| *Atf3* ^[35]^ | [NM_007498.3](https://www.ncbi.nlm.nih.gov/nuccore/NM_007498.3) | GTGCCTGCAGAAAGAGTCAGA | GAGGTTCCTCTCGTCTTCCG | 172 |
| *B2m* | NM_009735.3 | ACCGTCTACTGGGATCGAGA* | TGCTATTTCTTTCTGCGTGCAT* | 126 |
| *Gapdh* ^[10]^ | NM_008084 | TCCATGACAACTTTGGCATTG* | CAGTCTTCTGGGTGGCAGTGA* | 72 |
| *Mult1* | NM_029975.2 | AGTCACCTGTGTTTATGCAGATTG* | TGCTGTCGGAAATTCATCTTCTT* | 116 |
| *Pan-Raet1* ^[10]^ | - | AACGGGCTGGATGATGCAC* | TGGGGTAGGATCCTTGATGGT* | 60 |
| *Raet1a* | NM_009016.1 | CCCGAATGCAGACAGGAAGT | TGGAAGCGGGGAAGTTGATG | 111 |
| *Raet1b* | NM_009017.1 | CAGCAAATGCCACTGAAGTGAAG | TGGGTAACCATTGGTCTTGTGAG | 119 |
| *Raet1c* | NM_009018.1 | TCCTCTCTGGTATGAAGCGAAG | ATCGTTGCACAAGGTCCCC | 404 |
| *Raet1d* | NM_020030.2 | CAACTTGACCATCAAGGCTCCTA | GATAAGTATTTCACCCACGAAGCA | 67 |
| *Raet1e* ^[36]^ | NM_198193.2 | CAGGTGACCCAGGGAAGATG | CTCAACTCCTGGCACAAATCG | 79 |
| *Qa1b* | NM_010398.3 | TATTTCACCACCGCCGTGTC | GTCCACGTAGCCGACAATGA | 69 |
| Asterisk (*) indicates primers which cross an exon-exon boundary. | | | | |

## Supplementary Table 7. Primer sequences for single cell nested PCR

| Target gene | GenBank reference | Primer pair | Forward sequence (5’ – 3’) | Reverse sequence (5’ – 3’) | Product size (bp) |
| --- | --- | --- | --- | --- | --- |
| *Advillin* | NM_009635.3 | Outer | GCTACATCGTCCTCTCGACC* | CATTTCCACCTCCGTGGCTT* | 332 |
|  |  | Inner | CCACTTTTGGATCGGGAAGGA | TTGGTCTCCACGTGCTTCAT | 210 |
| *Gapdh* | NM_001289726.1;  NM_008084.3 | Outer | AACAGCAACTCCCACTCTTC* | TGGGTGCAGCGAACTTTAT* | 329 |
|  |  | Inner | ACTCCCACTCTTCCACCTTC* | TGAGGGAGATGCTCAGTGTT* | 230 |
| *Mult1* | NM_029975.2 | Outer | GTGCCTACTTCACCACAATCC* | TCGTCTGAAGTCAACAGCAC* | 215 |
|  |  | Inner | AGTCACCTGTGTTTATGCAGATTG* | TGCTGTCGGAAATTCATCTTCTT* | 116 |
| *Pan-Raet1* | - | Outer | GCTGTTGCCACAGTCACATC* | CCTGGGTCACCTGAAGTCAT | 270 |
|  |  | Inner | GCTGTTGCCACAGTCACATC* | TGGGGTAGGATCCTTGATGGT* | 172 |
| *Runx1* | NM_001111021-3;  NM_009821.3 | Outer | CATCCCCACCGTGGTCCTAT* | AGGTAGGTGTGGTAGCGAGA* | 316 |
|  |  | Inner | TGACCAGTCCTACCAGTACCT* | GAGCCGTTGAGAGTCGACTG* | 131 |
| *Scn10a* | NM_001205321.1;  NM_009134.3 | Outer | TCCCACGGCCTTTGAATAAG | CCAGCCGTTGGTGAAGTAATA | 245 |
|  |  | Inner | CTTTGAATAAGTACCAGGGCTTC | GAACATCTTCATCACACACTCG | 203 |
| *Trka* | NM_001033124.1 | Outer | CTTCTCGCCAGTGGACGGTA* | GAGCCTTTGCCCTCAGTAGG | 270 |
|  |  | Inner | ACAGCACATCAAGAGACCCA | TAATACAGCAGGGCGGTTGA | 167 |
| *Trpv1* | NM_001001445.2 | Outer | CATGCTCATTGCTCTCATGG | AACCAGGGCAAAGTTCTTCC | 352 |
|  |  | Inner | CATGGGCGAGACTGTCAAC | CTGGGTCCTCGTTGATGATG | 248 |
| Asterisk (*) indicates primers which cross an exon-exon boundary. | | | | | |

# Supplementary References

[1]. Nassar MA, Stirling LC, Forlani G, Baker MD, Matthews EA, Dickenson AH, et al. Nociceptor-specific gene deletion reveals a major role for Nav1.7 (PN1) in acute and inflammatory pain. Proceedings of the National Academy of Sciences of the United States of America. 2004;101(34):12706-11.

[2]. Song H, Yao E, Lin C, Gacayan R, Chen MH, Chuang PT. Functional characterization of pulmonary neuroendocrine cells in lung development, injury, and tumorigenesis. Proceedings of the National Academy of Sciences of the United States of America. 2012;109(43):17531-6.

[3]. Olson W, Abdus-Saboor I, Cui L, Burdge J, Raabe T, Ma M, et al. Sparse genetic tracing reveals regionally specific functional organization of mammalian nociceptors. Elife. 2017;6:e29507.

[4]. Abraira VE, Kuehn ED, Chirila AM, Springel MW, Toliver AA, Zimmerman AL, et al. The Cellular and Synaptic Architecture of the Mechanosensory Dorsal Horn. Cell. 2017;168(1-2):295-310 e19.

[5]. Feng G, Mellor RH, Bernstein M, Keller-Peck C, Nguyen QT, Wallace M, et al. Imaging neuronal subsets in transgenic mice expressing multiple spectral variants of GFP. Neuron. 2000;28(1):41-51.

[6]. Cavanaugh DJ, Chesler AT, Jackson AC, Sigal YM, Yamanaka H, Grant R, et al. Trpv1 reporter mice reveal highly restricted brain distribution and functional expression in arteriolar smooth muscle cells. The Journal of neuroscience : the official journal of the Society for Neuroscience. 2011;31(13):5067-77.

[7]. Wu S, Wu Y, Capecchi MR. Motoneurons and oligodendrocytes are sequentially generated from neural stem cells but do not appear to share common lineage-restricted progenitors in vivo. Development. 2006;133(4):581-90.

[8]. Madisen L, Zwingman TA, Sunkin SM, Oh SW, Zariwala HA, Gu H, et al. A robust and high-throughput Cre reporting and characterization system for the whole mouse brain. Nat Neurosci. 2010;13(1):133-40.

[9]. Barry AM, Zhao N, Yang X, Bennett DL, Baskozos G. Deep RNA-seq of male and female murine sensory neuron subtypes after nerve injury. Pain. 2023;164(10):2196-215.

[10]. Davies AJ, Kim HW, Gonzalez-Cano R, Choi J, Back SK, Roh SE, et al. Natural Killer Cells Degenerate Intact Sensory Afferents following Nerve Injury. Cell. 2019;176(4):716-28 e18.

[11]. Cobos EJ, Nickerson CA, Gao F, Chandran V, Bravo-Caparros I, Gonzalez-Cano R, et al. Mechanistic Differences in Neuropathic Pain Modalities Revealed by Correlating Behavior with Global Expression Profiling. Cell Rep. 2018;22(5):1301-12.

[12]. Kim YH, Back SK, Davies AJ, Jeong H, Jo HJ, Chung G, et al. TRPV1 in GABAergic interneurons mediates neuropathic mechanical allodynia and disinhibition of the nociceptive circuitry in the spinal cord. Neuron. 2012;74(4):640-7.

[13]. Kim HW, Davies AJ, Oh SB. In Vitro Visualization of Cell-to-Cell Interactions Between Natural Killer Cells and Sensory Neurons. Methods Mol Biol. 2022;2463:251-68.

[14]. Clark AJ, Kaller MS, Galino J, Willison HJ, Rinaldi S, Bennett DLH. Co-cultures with stem cell-derived human sensory neurons reveal regulators of peripheral myelination. Brain. 2017;140(4):898-913.

[15]. Chambers SM, Qi Y, Mica Y, Lee G, Zhang XJ, Niu L, et al. Combined small-molecule inhibition accelerates developmental timing and converts human pluripotent stem cells into nociceptors. Nat Biotechnol. 2012;30(7):715-20.

[16]. Clark AJ. Establishing Myelinating Cocultures Using Human iPSC-Derived Sensory Neurons to Investigate Axonal Degeneration and Demyelination. Methods Mol Biol. 2020;2143:111-29.

[17]. Schindelin J, Arganda-Carreras I, Frise E, Kaynig V, Longair M, Pietzsch T, et al. Fiji: an open-source platform for biological-image analysis. Nat Methods. 2012;9(7):676-82.

[18]. Kayasandik CB, Labate D. Improved detection of soma location and morphology in fluorescence microscopy images of neurons. J Neurosci Methods. 2016;274:61-70.

[19]. DaviesLab-Oxford. (2025) NKG2D-binding-murine-axons-lineage-analysis-pipeline GitHub (Zenodo). <https://doi.org/10.5281/zenodo.15921009> (Accessed July 2025)

[20]. DaviesLab-Oxford. (2025) NKG2D-binding-hiPSCdSN-analysis-pipeline GitHub (Zenodo). <https://doi.org/10.5281/zenodo.15920869> (Accessed July 2025)

[21]. DaviesLab-Oxford. (2025) Fragmentation-axons-automated-analysis-Fiji GitHub (Zenodo). <https://doi.org/10.5281/zenodo.15920937> (Accessed July 2025)

[22]. Schmittgen TD, Livak KJ. Analyzing real-time PCR data by the comparative C(T) method. Nature protocols. 2008;3(6):1101-8.

[23]. Ye J, Coulouris G, Zaretskaya I, Cutcutache I, Rozen S, Madden TL. Primer-BLAST: a tool to design target-specific primers for polymerase chain reaction. BMC Bioinformatics. 2012;13:134.

[24]. Kibbe WA. OligoCalc: an online oligonucleotide properties calculator. Nucleic Acids Res. 2007;35(Web Server issue):W43-6.

[25]. Wangzhou A, McIlvried LA, Paige C, Barragan-Iglesias P, Shiers S, Ahmad A, et al. Pharmacological target-focused transcriptomic analysis of native vs cultured human and mouse dorsal root ganglia. Pain. 2020;161(7):1497-517.

[26]. Ray PR, Shiers S, Caruso JP, Tavares-Ferreira D, Sankaranarayanan I, Uhelski ML, et al. RNA profiling of human dorsal root ganglia reveals sex differences in mechanisms promoting neuropathic pain. Brain. 2023;146(2):749-66.

[27]. Tavares-Ferreira D, Shiers S, Ray PR, Wangzhou A, Jeevakumar V, Sankaranarayanan I, et al. Spatial transcriptomics of dorsal root ganglia identifies molecular signatures of human nociceptors. Sci Transl Med. 2022;14(632):eabj8186.

[28]. Yu H, Nagi SS, Usoskin D, Hu Y, Kupari J, Bouchatta O, et al. Leveraging deep single-soma RNA sequencing to explore the neural basis of human somatosensation. Nat Neurosci. 2024.

[29]. Clark AJ, Kugathasan U, Baskozos G, Priestman DA, Fugger N, Lone MA, et al. An iPSC model of hereditary sensory neuropathy-1 reveals L-serine-responsive deficits in neuronal ganglioside composition and axoglial interactions. Cell Rep Med. 2021;2(7):100345.

[30]. Bhuiyan SA, Renthal W. (2024) Harmonized DRG and TG reference atlas. Painseq (Shinyapp). Available from: <https://painseq.shinyapps.io/harmonized_painseq_v1/> (Accessed June 2025)

[31]. Heming M, Hörste GMz. (2024) Single nuclei sural nerve atlas. (Cerebro). Available from: <https://osmzhlab.uni-muenster.de/shiny/cerebro_pns_atlas/> (Accessed June 2025)

[32]. Krauter D, Ernfors P. (2025) Mouse DRG Integrated Atlas. Ernforslab (Shinyapp). Available from: <https://ernforslab.shinyapps.io/integratedDRGatlas/> (Accessed June 2025)

[33]. Krauter D, Kupari J, Usoskin D, Su J, Hu Y, Zhang MD, et al. Spatial organization, chromatin accessibility and gene-regulatory programs defining mouse sensory neurons. Commun Biol. 2025;8(1):908.

[34]. Bhuiyan SA, Xu M, Yang L, Semizoglou E, Bhatia P, Pantaleo KI, et al. Harmonized cross-species cell atlases of trigeminal and dorsal root ganglia. Sci Adv. 2024;10(25):eadj9173.

[35]. Dawes JM, Weir GA, Middleton SJ, Patel R, Chisholm KI, Pettingill P, et al. Immune or Genetic-Mediated Disruption of CASPR2 Causes Pain Hypersensitivity Due to Enhanced Primary Afferent Excitability. Neuron. 2018;97(4):806-22 e10.

[36]. Ogasawara K, Hamerman JA, Hsin H, Chikuma S, Bour-Jordan H, Chen T, et al. Impairment of NK cell function by NKG2D modulation in NOD mice. Immunity. 2003;18(1):41-51.
